# A kinetics-based model of haematopoiesis reveals extrinsic regulation of skewed lineage output from stem cells

**DOI:** 10.1038/s41556-026-01958-0

**Published:** 2026-05-25

**Authors:** Esther Rodríguez-Correa, Florian Grünschläger, Tamar Nizharadze, Natasha Anstee, Jude Al-Sabah, Vojtech Kumpost, Anastasia Sedlmeier, Congxin Li, Melanie Ball, Foteini Fotopoulou, Jeyan Jayarajan, Ian Ghezzi, Julia Knoch, Megan Druce, Kleo Aurich, Marleen Büchler-Schäff, Susanne Lux, Pablo Hernández-Malmierca, Julius Gräsel, Dominik Vonficht, Marta López-Osias, Elvira González-Saiz, Daniel Fernández-Pérez, Anna Mathioudaki, Judith Zaugg, Alejo Rodríguez-Fraticelli, Ralf Mikut, Andreas Trumpp, Thomas Höfer, Daniel Hübschmann, Simon Haas, Michael D. Milsom

**Affiliations:** 1 Division of Experimental Hematology, German Cancer Research Center (DKFZ), Heidelberg, Germany; 2 Heidelberg Institute for Stem Cell Technology and Experimental Medicine (HI-STEM gGmbH), Heidelberg, Germany; 3 Faculty of Biosciences, Heidelberg University, Heidelberg, Germany; 4 Division of Stem Cells and Cancer, German Cancer Research Center (DKFZ), Heidelberg, Germany; 5 Division of Theoretical Systems Biology, German Cancer Research Center (DKFZ), Heidelberg, Germany; 6 Institute for Automation and Applied Informatics, Karlsruhe Institute of Technology, Karlsruhe, Germany; 7 Computational Oncology, Molecular Precision Oncology Program, National Center for Tumor Diseases Heidelberg and German Cancer Research Center (DKFZ), Heidelberg, Germany; 8 Institute for Research in Biomedicine, Barcelona, The Barcelona Institute of Science and Technology, Barcelona, Spain; 9 Molecular Systems Biology Unit, European Molecular Biology Laboratory, Heidelberg, Germany; 10 Division of AI in Oncology, German Cancer Research Centre (DKFZ), Heidelberg, Germany; 11 Department of Biomedicine, University Hospital Basel, University of Basel, Basel, Switzerland; 12 ICREA, Catalan Institution for Research and Advanced Studies Barcelona, Barcelona, Spain; 13 DKFZ–ZMBH Alliance, Heidelberg, Germany; 14 German Cancer Consortium (DKTK), Heidelberg, Germany; 15 Innovation and Service Unit for Bioinformatics and Precision Medicine, German Cancer Research Center (DKFZ), Heidelberg, Germany; 16 Division of Translational Precision Medicine, Institute of Human Genetics, Heidelberg University, Heidelberg, Germany; 17 Berlin Institute of Health at Charité Universitätsmedizin, Berlin, Germany; 18 Berlin Institute for Medical Systems Biology, Max Delbrück Center for Molecular Medicine in the Helmholtz Association, Berlin, Germany; 19 Precision Healthcare University Research Institute, Queen Mary University of London, London, UK

**Keywords:** Haematopoietic stem cells, Regeneration, Stem-cell differentiation, Computer modelling, Self-renewal

## Abstract

Haematopoietic stem cells (HSCs) display extensive molecular and functional heterogeneity. However, a cohesive model that explains the relationship and biological relevance of these diverse HSC states remains elusive. Here, by performing single-cell transplantations of over 1,000 highly purified murine long-term HSCs combined with in-depth phenotyping of their clonal progeny, we define kinetics-based reconstitution parameters which aligned HSCs into a single hierarchical trajectory reflective of functional potency. This approach revealed that previously identified lineage biases are actually transitory states along this linear trajectory, not a discrete stable condition. Single-cell secondary transplantations validated hierarchical ordering based on reconstitution kinetics, whereas mathematical modelling combined with experimental modulation of lineage-biased blood production revealed that apparent lineage-biased outputs actually arise from cell-extrinsic feedback regulation and clonal competition between slow- and fast-engrafting clones to fill mature lineages to their compartment size limit. This study reconciles multiple layers of HSC heterogeneity into a unifying framework.

## Abstract (web summary)

Single-cell transplantations of long-term haematopoietic stem cells (HSCs), in-depth phenotyping and mathematical modelling identify reconstitution kinetics as a unifying metric of HSC potency and extrinsic regulation as a driver of HSC lineage bias.

## Main

Haematopoietic stem cells (HSC) have canonically been perceived as a uniform entity with consistent self-renewal and multipotent characteristics^1^. However, over the course of the last two decades, numerous studies have characterized an ever-increasing spectrum of functional and molecular heterogeneity within this compartment^2,3^. Transplantation studies of variable immunophenotypically defined populations demonstrated profound differences in their capacity to sustain hematopoiesis over time, identifying HSCs with long- or short-term repopulation capacity, as well as multipotent progenitors with transient engraftment and limited self-renewal^4^. Single-cell transplantation and barcoding experiments have shown that, even within the immunophenotypically homogenous long-term (LT)-HSC compartment, the quantitative and qualitative output of individual HSCs is highly heterogeneous^5–8^. In this context, multiple studies have reported pronounced biases of HSCs regarding the generation of distinct lineages of the haematopoietic system, including myeloid and lymphoid-biased output, as well as individual HSCs that are capable of generating a more balanced multilineage reconstitution pattern^6,7,9,10^. More recent studies also identified platelet-biased HSCs, which appear to reside at the apex of the haematopoietic hierarchy^8,11–13^. Several physiological roles of lineage biases have been suggested^12,14^, and although some insights have been made into the underlying mechanisms that might mediate this phenomenon^15–21^, a comprehensive understanding of the causes and consequences of lineage biases in the HSC compartment remains to be elucidated. In line with the observed functional heterogeneity within the HSC pool, single-cell multi-omic profiling has also revealed extensive molecular heterogeneity of HSCs, correlating with distinct stemness and lineage bias patterns^8,13,22–27^. Together, these functional and molecular studies have challenged the classical model of hematopoiesis, which assumes HSCs are multipotent and homogeneous in lineage contribution^2,3,28^. However, a unifying model that explains the origins and interrelationships of these multiple HSC subtypes remains elusive. HSC functional potency and lineage bias have been typically defined using strict thresholds applied to the proportions of mature cell progeny measured at arbitrary post-transplantation timepoints, capturing only a limited snapshot of their output. This approach has led to the accepted concept of discrete HSC subtypes, while neglecting the continuous nature of the data and wide spectrum of HSC behaviours. To overcome these limitations, we performed an in-depth analysis of HSC clonal reconstitution through extensive primary and serial secondary single-cell transplantations, combined with single-cell molecular profiling of clonal systems and mathematical modelling, to establish a unifying framework that clarifies the interrelationships among these distinct layers of molecular and functional heterogeneity.

## Results

### Comprehensive characterization of clonally derived haematopoietic systems links HSC heterogeneity to reconstitution kinetics

**Fig. 1:**
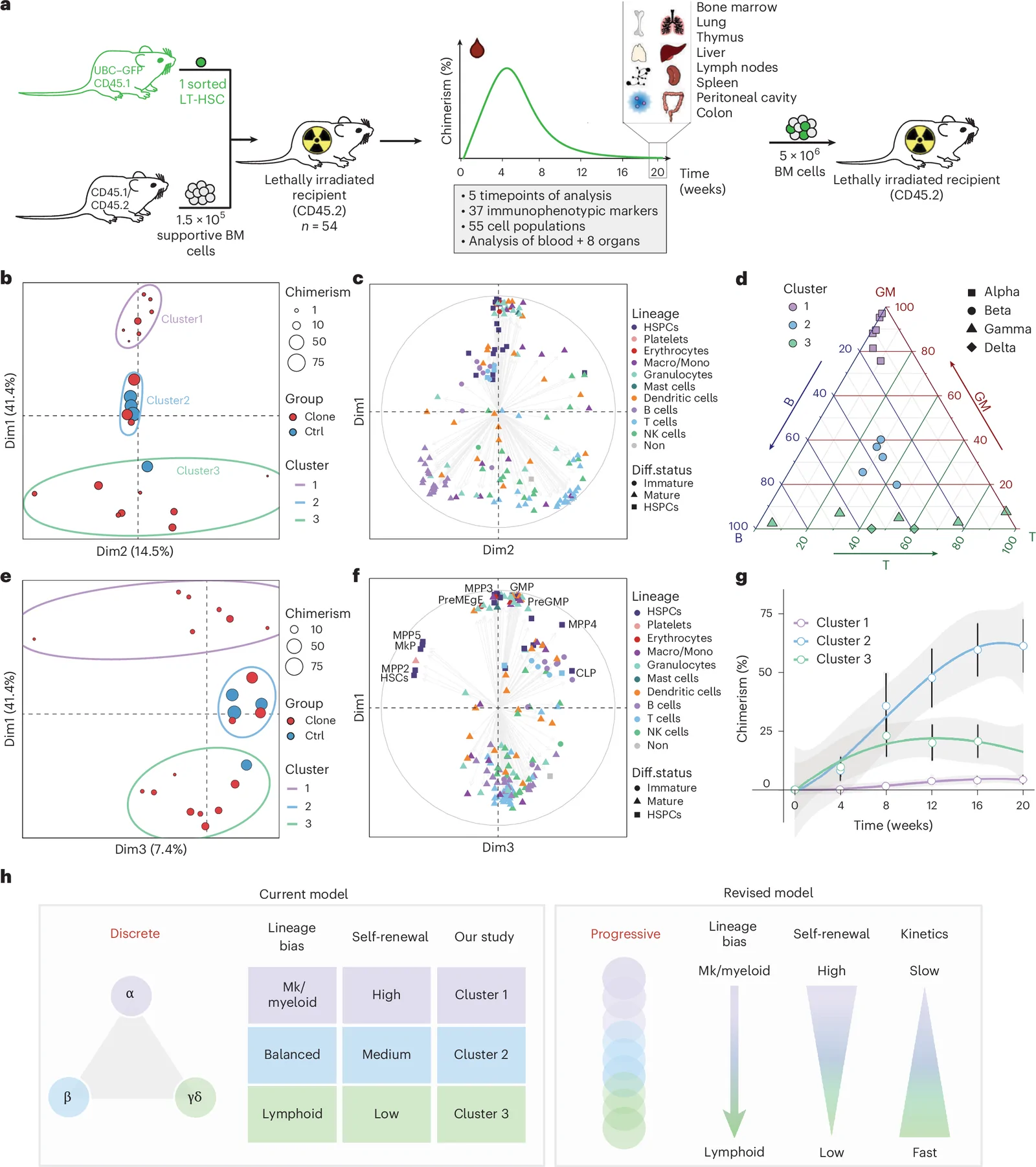
Comprehensive characterization of clonally derived haematopoietic systems links HSC heterogeneity to reconstitution kinetics. **a**, A schematic overview of experimental design. **b**, PCA representing donor chimerism across all cell types at 20 weeks post transplantation. The average percentage donor chimerism across all 55 cell types is represented by dot size. Polyclonal controls receiving donor HSCs (Ctrl) are highlighted in blue, and recipients of single HSCs in red. Haematopoietic systems were crudely clustered into three groups based on hierarchical clustering of the top three PCA dimensions. **c**, Variable contribution map highlighting the loadings by differentiation status (symbols) and lineage (colours) contributing to PCA dimensions 1 (Dim 1) and 2 (Dim 2). **d**, Ternary plot comparing previously defined alpha, beta, gamma and delta clonal reconstitution patterns with our three clusters. Alpha through delta classification was determined by ratio of myeloid to lymphoid cells in PB at 20 weeks post transplantation, as previously performed in ref. ^6^. **e**, Projection of Dim 1 and dimension 3 (Dim3) of PCA described in **b**. **f**, Variable contribution map of Dim 1 and Dim3 from PCA. **g**, Time course of average donor chimerism (±s.d.) across all blood cells, subsegregated according to the three clusters. Mean chimerism was fitted using a third-degree polynomial function with 95% confidence intervals (CI) highlighted in grey. **h**, A schematic comparison of the current cluster-based model of HSC subtypes versus our revised model based on a linear continuum of HSC states. *n* = 18 clonal systems in **b**–**g** and *n* = 4 polyclonal controls in **b** and **e**. UBC–GFP, ubiquitin C–green fluorescent protein; NK cell, natural killer cell; GM, granulocyte–monocyte; MPP, multipotent progenitor; PreMegE, pre-megakaryocyte-erythrocyte; GMP, granulocyte–monocyte progenitor; CLP, common lymphoid progenitor. Diagram in **h** created in BioRender; Milsom, M. https://biorender.com/gwm5s48 (2026). Source data

**Extended Data Fig. 1:**
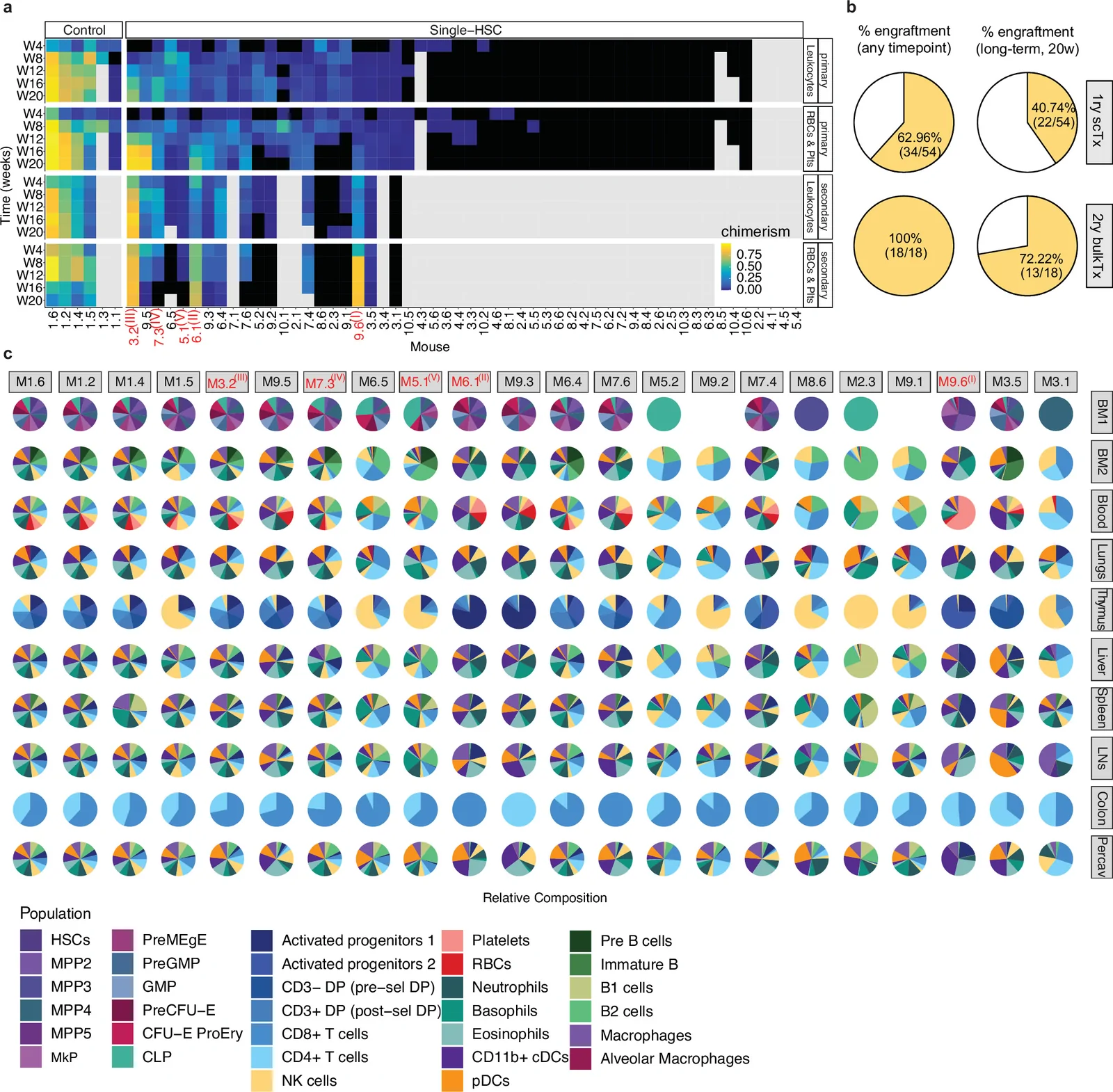
Overview of single-HSC transplantation experiment. **a**) Heatmap of peripheral blood donor chimerism over time split between average leukocyte or erythrocyte and platelet chimerism of transplanted mice within primary and secondary transplantation ordered by their average chimerism. Black color indicates chimerism <0.1%, gray color indicates missing value. **b**) Pie charts displaying the percentage of transplanted clones with successful engraftment (>0.1% chimerism in peripheral blood) at any time point post-transplantation (left), and with positive long-term chimerism (at 20 weeks post-transplantation) (right), for primary (top) and secondary (bottom) transplants. 1ry scTx: primary single-cell transplantation; 2ry bulkTx: secondary bulk bone marrow transplantation. **c**) Clonal hematopoietic compositions at 20 weeks post-transplant split by organ and cell populations are highlighted by color. BM1 and BM2 correspond to two different antibody stainings of the bone marrow. Abbreviations: HSC: hematopoietic stem cell; RBCs: red blood cells; Plts: platelets; W: week; BM: bone marrow; ScTx: single-cell transplantation; LNs: lymph nodes, MPP: multipotent progenitor; MkP: megakaryocyte progenitor; PreMegE: pre-megakaryocyte-erythrocyte; GMP: granulocyte-monocyte progenitor; PreCFU-E: pre-colony-forming-unit-erythrocyte; CFU-E-ProEry: colony-forming-unit-erythrocyte-proerythroblast; CLP: common lymphoid progenitor; DP: double positive; cDC: conventional dendritic cell; pDC: plasmacytoid dendritic cell; NK: natural killer cell. Representative clones I-V from Fig. 3 are highlighted in red text and can be identified by annotation in superscript. Source data

**Extended Data Fig. 2:**
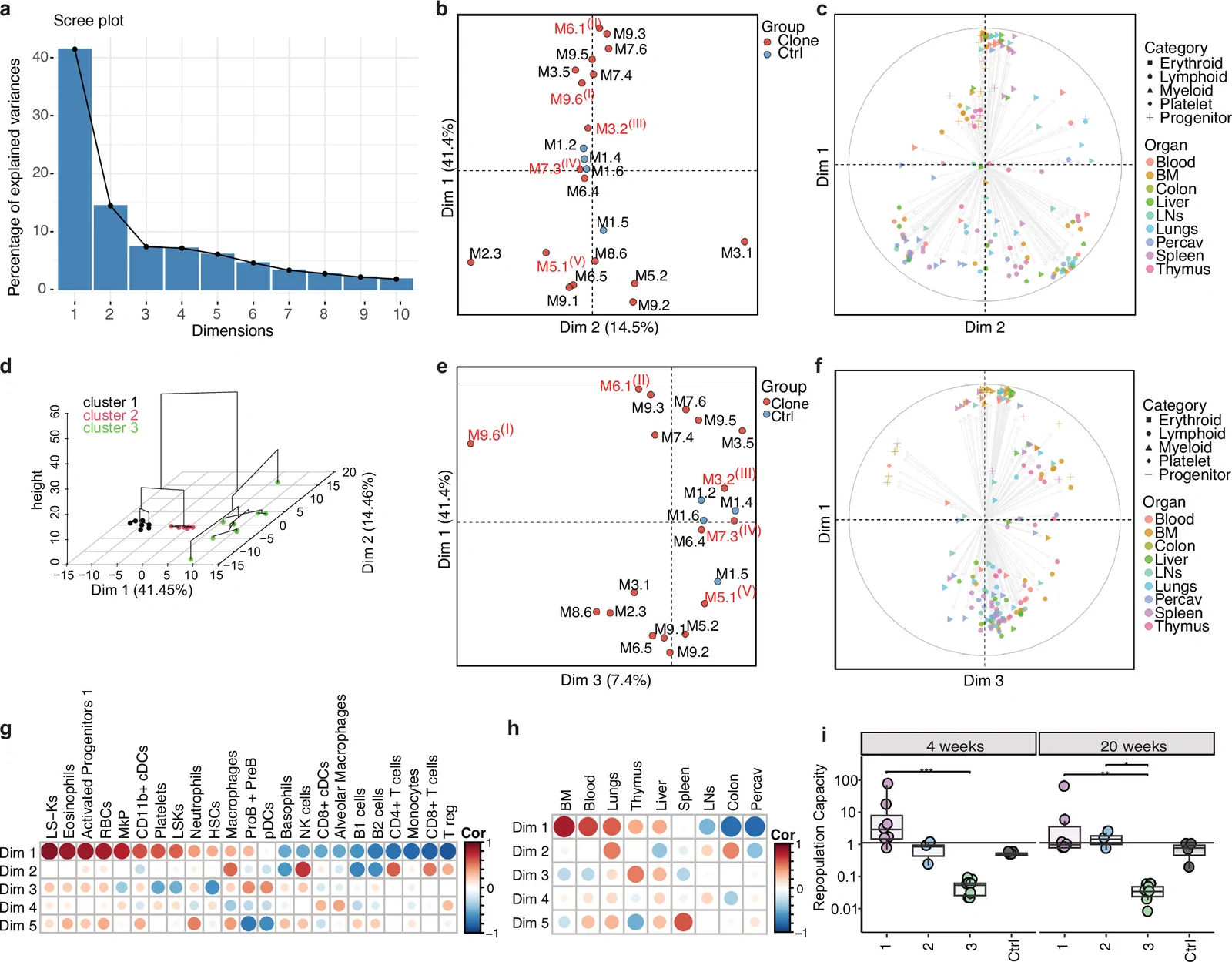
Principal component analysis of clonally-derived hematopoietic systems. **a**) Scree plot showing the percentage of explained variance by each principal component (dimensions 1 to 10). **b**) First and second principal component projections. Individual hematopoietic systems are labeled by experiment ID and colored by group (single HSC-derived: red, polyclonal control: blue). **c**) Variable contribution map highlighting the distribution of clonal systems between the first two dimensions based on organ tropism and lineage contribution. **d**) Hierarchical clustering on principal components based on the first three principal components. **e**) First and third principal component projections, see (**b**). **f**) Variable contribution map of first and third principal component projections, see (**c**). **g-h**) Spearman correlation coefficients between active and supplementary variables and dimensions, highlighting associations between principal components and cell types (**g**) as well as organs (**h**). **i**) Relative repopulation capacity (defined by the ratio of chimerism in secondary and primary transplantations) across the three clusters defined in Fig. 1, and shown by the polyclonal controls (Ctrl). If not stated otherwise, significant differences between groups were tested globally by Kruskal Wallis test and post hoc by two-sided Wilcoxon rank-sum test. For multiple comparisons, p values were corrected according to Benjamini-Hochberg. Significance is indicated by: * for p < 0.05, ** for p < 0.01, *** for p < 0.001. The standard deviation is indicated by error bars. Box plots: center line, median; box limits, first and third quartile; whiskers, smallest/largest value no further than 1.5*IQR from corresponding hinge. Abbreviations: Ctrl: control; HSPC: hematopoietic stem and progenitor cell; cDC: conventional dendritic cell; pDC, plasmacytoid dendritic cell; NK cell: natural killer cell; RBC: red blood cell; BM: bone marrow; LN: lymph node; PerCav: peritoneal cavity; ctrl: control; dim: dimension; LS-K: Lineage-Sca1-cKit + ; MkP: megakaryocyte progenitor; LSK: Lineage-Sca1+cKit + . Dim = PCA dimension. Representative clones I-V from Fig. 3 are highlighted in red text and can be identified by annotation in superscript. Source data

**Extended Data Fig. 3:**
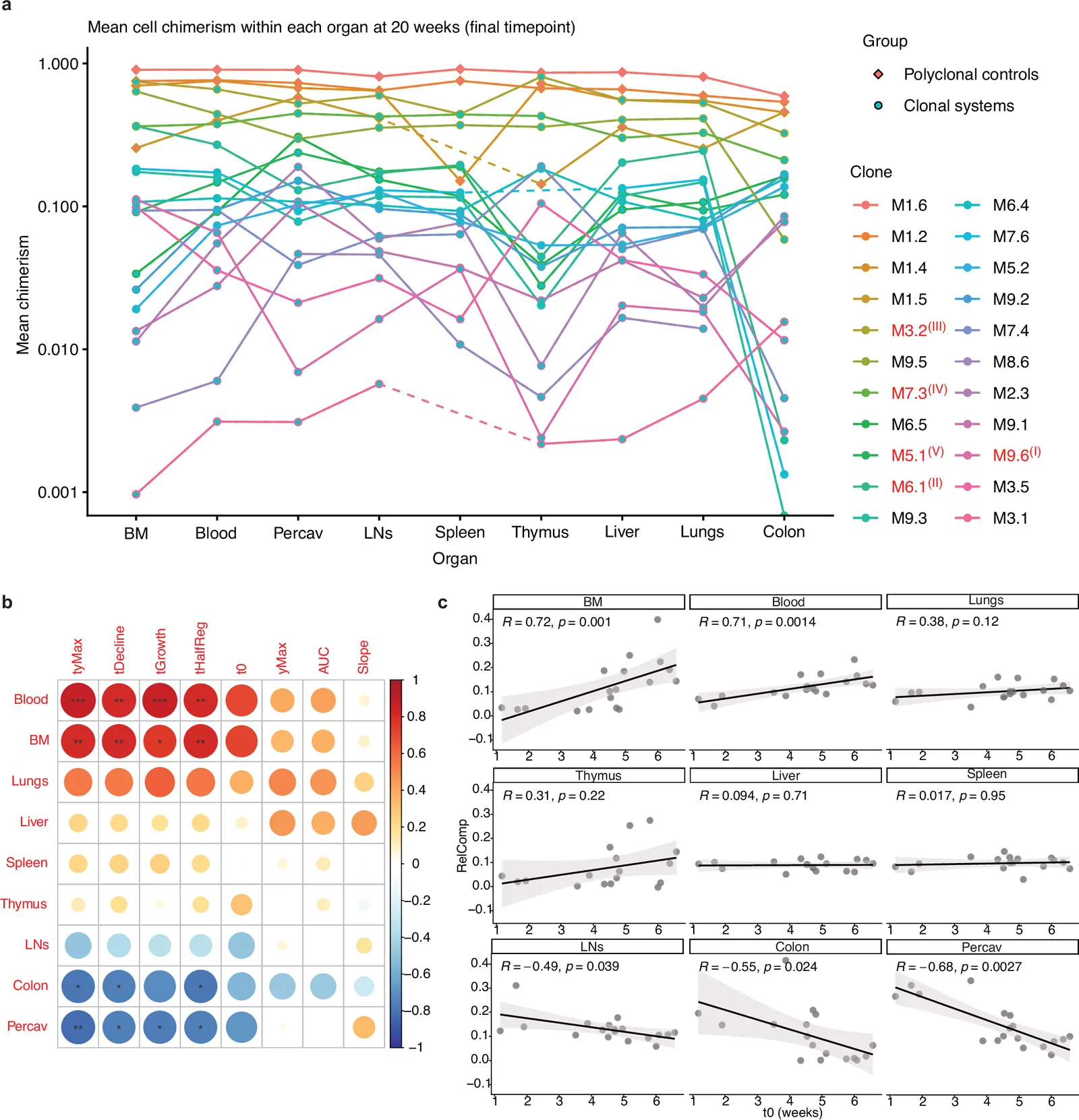
Organ tropism of clonally-derived hematopoietic cells post-transplantation and correlation with engraftment kinetics. **a**) Line plot of mean donor chimerism within each organ of 22 long-term engrafting HSC-transplanted mice 20 weeks post transplantation (4 polyclonal controls and 18 single-HSC clones). Polyclonal controls are represented as diamonds, clonally-derived systems are represented as circles. Dotted lines indicate a missing value between 2 datapoints. **b-c**) Spearman correlation between clonal contribution to each organ (that is “organ-tropism”) and its mean kinetic parameter of organ-specific cell types. **b**) Correlation matrix highlighting the correlation coefficient by size and color and the significance (adjusted p value, Holm method) by as follows: * for p < 0.05, ** for p < 0.01, *** for p < 0.001. **c**) Exemplary independent correlation plots for each organ between relative chimerism and kinetic parameter t0 of individual clonal systems for each organ. Each dot represents a single HSC-derived hematopoietic system. Spearman’s Rho and individual p values are shown per comparison, and 95% CI are highlighted in grey. BM: bone marrow; LN: lymph node; PerCav: peritoneal cavity. Representative clones I-V from Fig. 3 are highlighted in red text and can be identified by annotation in superscript. Source data

To generate high-resolution maps of HSC clonal heterogeneity, we transplanted single LT-HSCs (phenotypically defined as Lineage^−^, Kit^+^, Sca-1^+^, EPCRhi, CD34^−^, CD150^+^ and CD48^−^) from GFP-expressing mice^29^, along with supportive bone marrow (BM), into 54 lethally irradiated congenic mice (Fig. 1a). 6 mice each received 30 LT-HSCs to act as polyclonal controls. We subsequently performed a detailed kinetics-based analysis of clonal progeny output in recipient mice by interrogating the composition of peripheral blood (PB) every 4 weeks, as well as BM, spleen, lymph nodes, liver, lung, thymus, colon and peritoneal cavity at the 20-week post-transplantation endpoint, utilizing a total of 37 immunophenotypic markers to characterize 55 distinct HSC-derived cell populations. Overall, 34 mice (62.96%) showed donor chimerism >0.1% in any PB cell type in at least one timepoint, with 22 (40.74%) demonstrating sustained chimerism above this level at 20 weeks post transplant (Extended Data Fig. 1). Following bulk secondary transplantation of BM from recipients with sustained engraftment, all re-transplanted mice exhibited transient donor chimerism, and 13 out of 18 mice (72.22%) displayed detectable donor-derived cells at the 20-week endpoint. To gain a comprehensive overview of the spectrum of outcomes in recipient mice, we performed principal component analysis (PCA) using the clonal contributions of each transplanted HSC to all measured HSC-derived cell types across all assessed organs at the endpoint (Fig. 1b–f and Extended Data Figs. 2 and 3). The first dimension of the PCA distinguished clonal systems with enriched engraftment in haematopoietic stem and progenitor cells (HSPCs), erythroid, megakaryocyte and myeloid lineages from those which predominantly produced lymphoid cell types (Fig. 1b,c and Extended Data Fig. 2b,c). The second PCA dimension broadly segregated clonal systems with high contributions to B versus T cells, whereas the third PCA dimension separated clonal systems based on their specific chimerism in HSCs, multipotent progenitors, megakaryocyte progenitors (MkP) and platelets (Fig. 1e,f and Extended Data Fig. 2e,f). Finally, PCA dimension 3 identified previously described platelet-biased HSCs residing at the top of the haematopoietic hierarchy^12^. Notably, separating clonal systems by PCA dimension 1 aligned with a previously proposed classification of LT repopulating HSCs based on lineage biases^6^. That is, so-called myeloid-biased ‘α-HSCs’, balanced multilineage ‘β-HSCs’ and lymphoid-biased ‘γδ-HSCs’ (Fig. 1b,d). Based on this analysis, we crudely subsegregated these outcomes into three main clusters. Cluster 1 was characterized by high HSPC chimerism in the BM but relatively low mature haematopoietic cell chimerism and a strong bias towards platelet and myeloid output; cluster 2 exhibited intermediate HSPC chimerism and a balanced multilineage output; and cluster 3 was characterized by strong lymphoid bias and reduced levels of HSPCs in the BM (Fig. 1b,d,e and Extended Data Fig. 2h). Clonal systems from cluster 1 and 2 showed superior secondary transplantation capacities compared with those of cluster 3, consistent with previous findings^6^ (Extended Data Fig. 2i). Importantly, we additionally observed that the heterogeneity between the three clusters correlated with distinct reconstitution kinetics in the PB (Fig. 1g). Thus, transplanted HSCs within cluster 1 replenished blood cells very slowly with an overall steady increase in chimerism across the window of observation. HSCs within cluster 2 harboured strong engraftment potential and demonstrated more rapid reconstitution kinetics, repopulating up to 75% of all blood cell types after 16 weeks and plateauing around 20 weeks post transplantation. By contrast, HSCs within cluster 3 engrafted the fastest but then declined in their blood chimerism from 12 weeks post transplantation onwards. Collectively, these data recapitulate key findings of previous single cell HSC transplantation studies but additionally link patterns of functional heterogeneity with clonal reconstitution kinetics^6,8,30^. Although we applied clustering to delineate HSCs with differing self-renewal potential, lineage biases and reconstitution kinetics, it is important to note that this approach provides a very crude representation of the full spectrum of functional heterogeneity demonstrated across the different HSCs. We therefore wished to explore whether aligning individual HSC clones according to their reconstitution kinetics might provide a more accurate representation of their potency and hierarchical relationship (Fig. 1h).

### A quantitative framework for haematopoietic reconstitution kinetics identifies time-dependent parameters associated with stem cell self-renewal

**Fig. 2:**
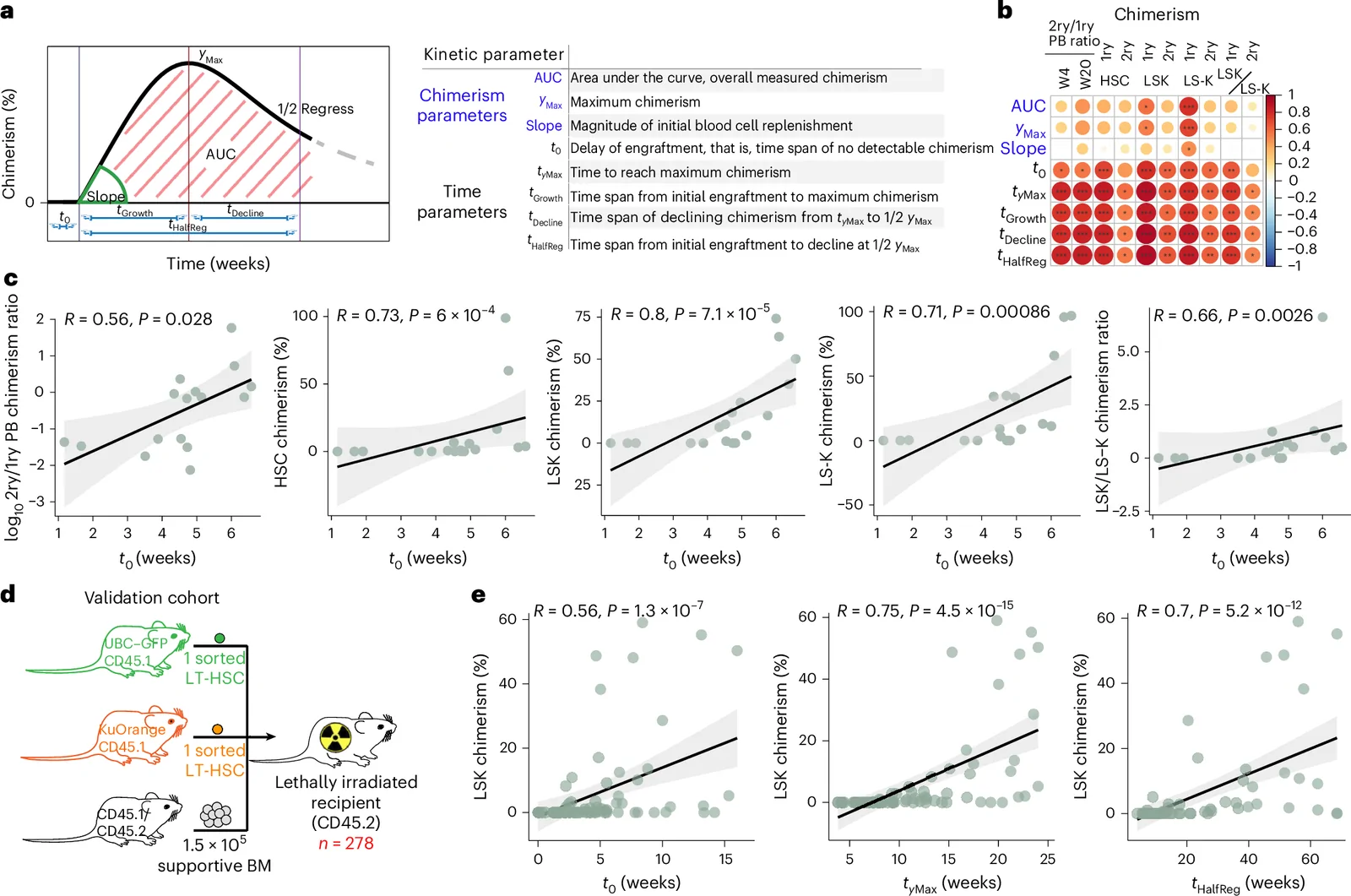
Haematopoietic reconstitution kinetics are linked to HSC functional potency. **a**, A schematic representation of kinetic and chimerism-based parameters calculated as a function of PB chimerism versus time. **b**, Two-sided Spearman correlation analysis between kinetic and chimerism-based parameters and metrics indicative of HSPC functionality and regeneration in primary (1ry) and secondary (2ry) recipients within the discovery cohort. These include: ratio of mean PB chimerism between 2ry and 1ry recipients at 4 (W4) and 20 weeks (W20) post transplantation; percentage donor chimerism in LT-HSC, lin^−^Sca-1^+^c-Kit^+^ HSPC (LSK) and more committed Lin^−^Sca-1^−^c-Kit^+^ progenitor (LS-K) compartments at W20 in 1ry and 2ry recipient BM and ratio of LSK to LS-K chimerism at W20 in 1ry and 2ry recipients. Spearman correlation coefficients are represented by dot size and colour. *P < 0.05, **P < 0.01, ***P < 0.001. **c**, Exemplary analysis from **b** showing correlation between the time delay (*t*_0_) of reconstitution and several metrics of HSC functional potency. Dots represent individual recipients. Spearman’s rho and significance are indicated. 95% confidence intervals (CI) of the fitted regression line are highlighted in grey. **d**, Experimental scheme of validation cohort, showing co-transplantation of two single LT-HSCs derived from either UBC–GFP or KuOrange donor mice. **e**, Two-sided Spearman correlation analysis between donor chimerism in LSK and the kinetic parameters *t*_0_, *t*_*y*__Max_ and *t*_HalfReg_ across the validation cohort. Each dot represents a single HSC-derived haematopoietic system. Spearman’s rho and significance are shown. 95% CI of the fitted regression line are highlighted in grey. Source data

**Extended Data Fig. 4:**
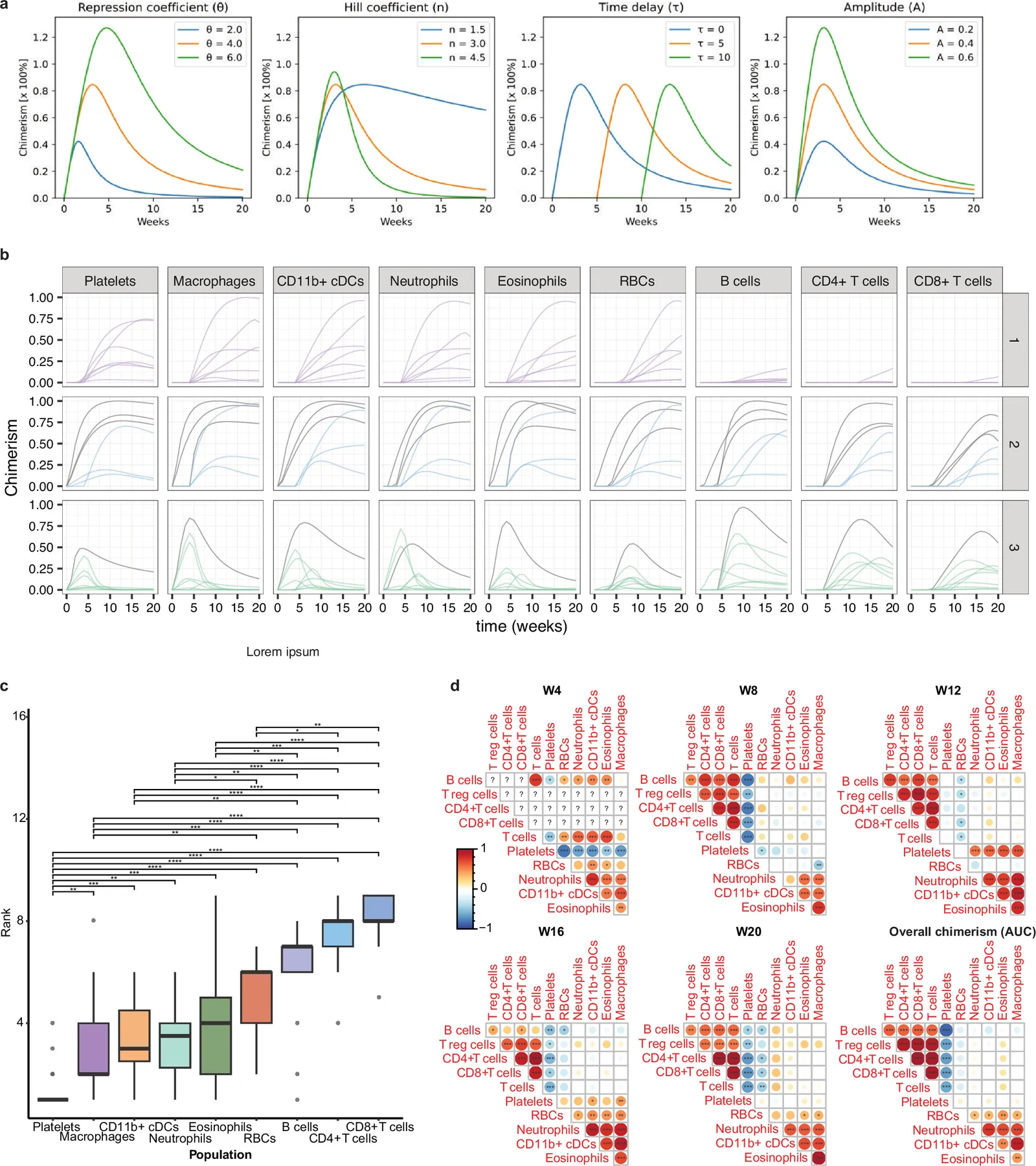
Kinetic curve fitting parameters. **a**) Exemplary behavior of single humped function fits with variation in its four coefficients. Repression coefficient and hill coefficient describe how stretched, or compressed the curve declines, the time delay marks the initial time point of growth and the amplitude controls the initial growth of the curve. **b**) Reconstitution kinetics fits of peripheral blood cells from transplanted HSCs separated by hierarchical clusters and cell type. Each coloured line corresponds to a single clonally-derived system. Polyclonal controls are colored in gray. **c**) Blood populations ordered by their ranked engraftment delay t0 within clonally-derived hematopoietic systems. Significant differences between cell types were tested globally by Kruskal Wallis test and post hoc by Dunn’s test. For multiple comparisons, p values were corrected according to Benjamini-Hochberg. **d**) Spearman correlation matrices of donor chimerism among blood populations at all bleeding time points, as well as for overall chimerism (AUC). Correlations of single HSC-derived blood cell compositions were calculated for each measured time point, or using the average overall chimerism values derived from AUC of each fitted curve, respectively. Cell types are ordered by angular order of eigenvectors from the correlation of AUC compositions. Significance is indicated by: * for p < 0.05, ** for p < 0.01, *** for p < 0.001, **** for p < 0.0001. Box plots: center line, median; box limits, first and third quartile; whiskers, smallest/largest value no further than 1.5*IQR from corresponding hinge. Dots indicate outliers. Abbreviations: cDC: conventional dendritic cell; RBC: red blood cell; W: week; AUC: area under the curve. Source data

To establish metrics that capture the continuum of functional heterogeneity within the HSC compartment, we next derived a quantitative framework describing blood reconstitution from LT-HSCs. Rather than using the previous clustering-based approach, which imposes arbitrary thresholds on cell outputs to categorize transplantation outcomes, we instead assessed reconstitution on a continuous scale and evaluated clonal contributions across the entire time course. This avoids over-reliance on a single, potentially misleading endpoint. To this end, we took the approach of modelling the reconstitution kinetics of PB production by fitting engraftment data across the entire time course, for every lineage, to a single-humped function (Fig. 2a). This function was chosen as it accurately mirrors blood cell reconstitution kinetics, including an initial delay phase, a growth phase, a plateau phase, followed ultimately by a decline phase following HSC exhaustion. Importantly, this approach allowed us to extract a range of kinetic parameters from the fitted curves, providing quantitative insights into the kinetics of blood cell repopulation (Fig. 2a and Extended Data Fig. 4). These included: the time delay between transplantation and the first production of cells into the PB (*t*_0_); the time taken to reach maximum chimerism following transplantation (*t*_*y*Max_); the time difference between *t*_0_ and *t*_*y*__Max_ (*t*_Growth_); the time taken to decline from maximum chimerism to half of the maximum value (*t*_Decline_, also referred to as half-life); and the time taken to transition from initial blood cell production all the way through to half of the maximum chimerism (*t*_HalfReg_). By introducing a quantitative framework that characterizes each clonal system through these kinetic parameters, broader associations could be objectively interrogated using standard statistical methodologies, independent of the more subjective HSC subtype clusters used in Fig. 1. In addition, we extracted more conventional chimerism-dependent parameters, including maximum chimerism (*y*_Max_) and overall cellular output into PB (area under the curve, AUC).

To systematically assess associations between HSC functional potency and reconstitution kinetics, we conducted correlation analyses comparing chimerism and kinetic-based parameters with reconstitution of the PB and the primitive HSPC compartment in the BM as an indicator of stem cell self-renewal (Fig. 2b,c). Surprisingly, conventional chimerism-dependent parameters showed only limited correlation with the degree of regeneration of the HSC and progenitor compartments in the BM. By contrast, all time-dependent kinetic parameters demonstrated a strong correlation with stemness-associated HSPC regeneration (Fig. 2b,c). This positive correlation between kinetic parameters and reconstitution was unique to the BM and PB, whereas the other organs analysed showed no or negative associations (Extended Data Fig. 3).

**Extended Data Fig. 5:**
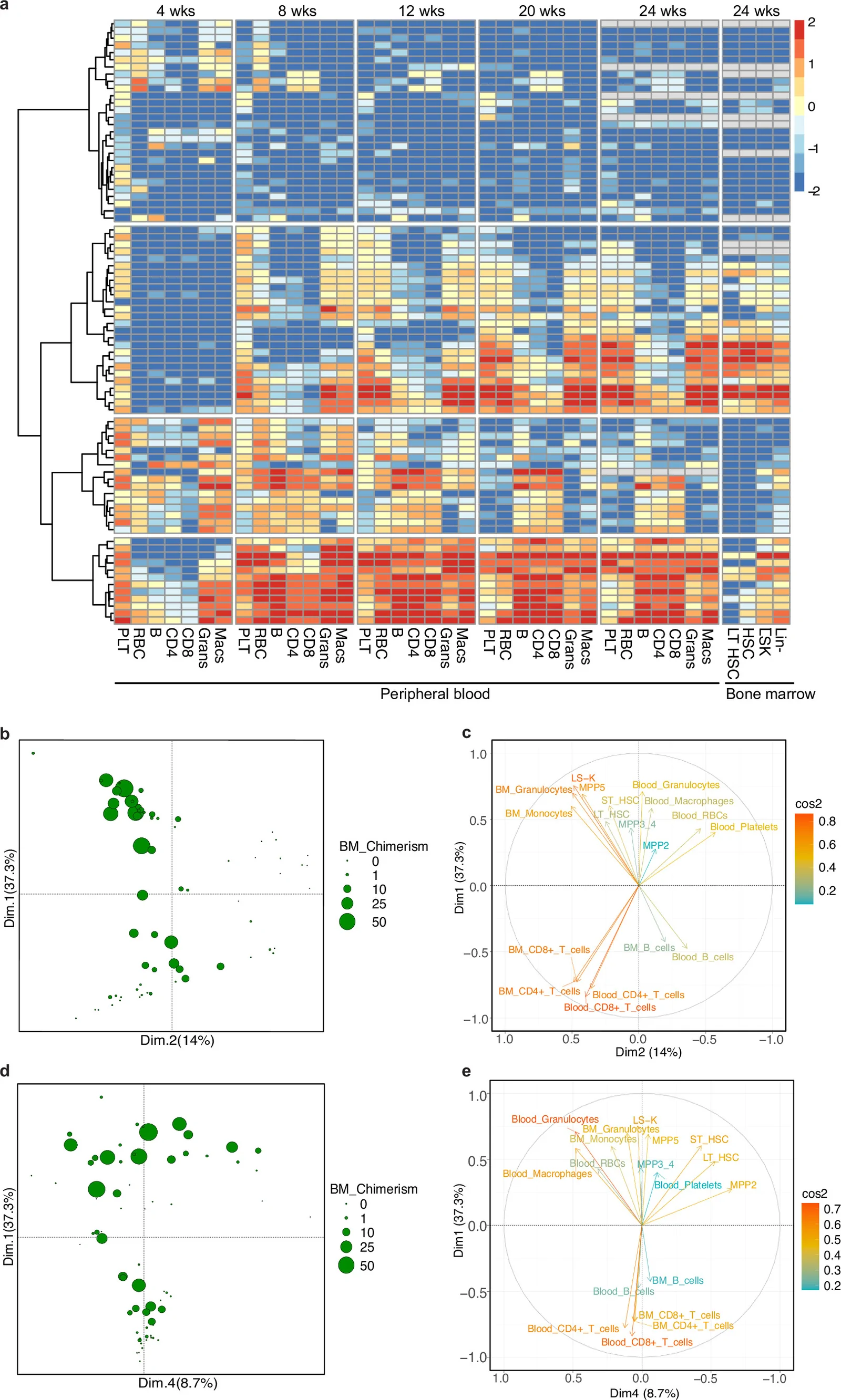
Validation cohort confirms reconstitution kinetics as a central feature associated with HSC potency and lineage biases. **a**) Heatmap displaying the percentage of chimerism in peripheral blood at different time points post-transplantation and in the bone marrow at the endpoint (24 weeks). Chimerism values are presented as log_10_-transformed percentages. Each row represents a single HSC transplantation that displayed positive chimerism (>0.1% in peripheral blood) at any time point. Agglomerative clustering, named AGNES (AGglomerative NESting) was used. **b**) Principal component analysis (PCA) considering the cellular composition of all HSC-derived cell types at the endpoint of the primary transplant (week 24). The first two components are displayed and the average chimerism is highlighted by dot size. **c**) Variable contribution map of (**b**) highlighting the loadings by differentiation status and lineage. **d**) First and fourth principal component projections of PCA from (**b**). Overall chimerism is highlighted by dot size. **e**) Variable contribution map of (**b**) highlighting the loadings by differentiation status and lineage from (**d**). Abbreviations: wks: weeks; PLT: platelet; RBC: red blood cell; B: B cell; CD4: CD4 + T cell; CD8: CD8 + T cell; Grans: granulocytes; Macs: macrophages; LT-HSC: long-term hematopoietic stem cell; LSK: Lineage-Sca1+cKit + ; Lin-: lineage negative; BM: bone marrow; Dim: dimension; MPP: multipotent progenitor; ST-HSC: short-term hematopoietic stem cell. Source data

**Extended Data Fig. 6:**
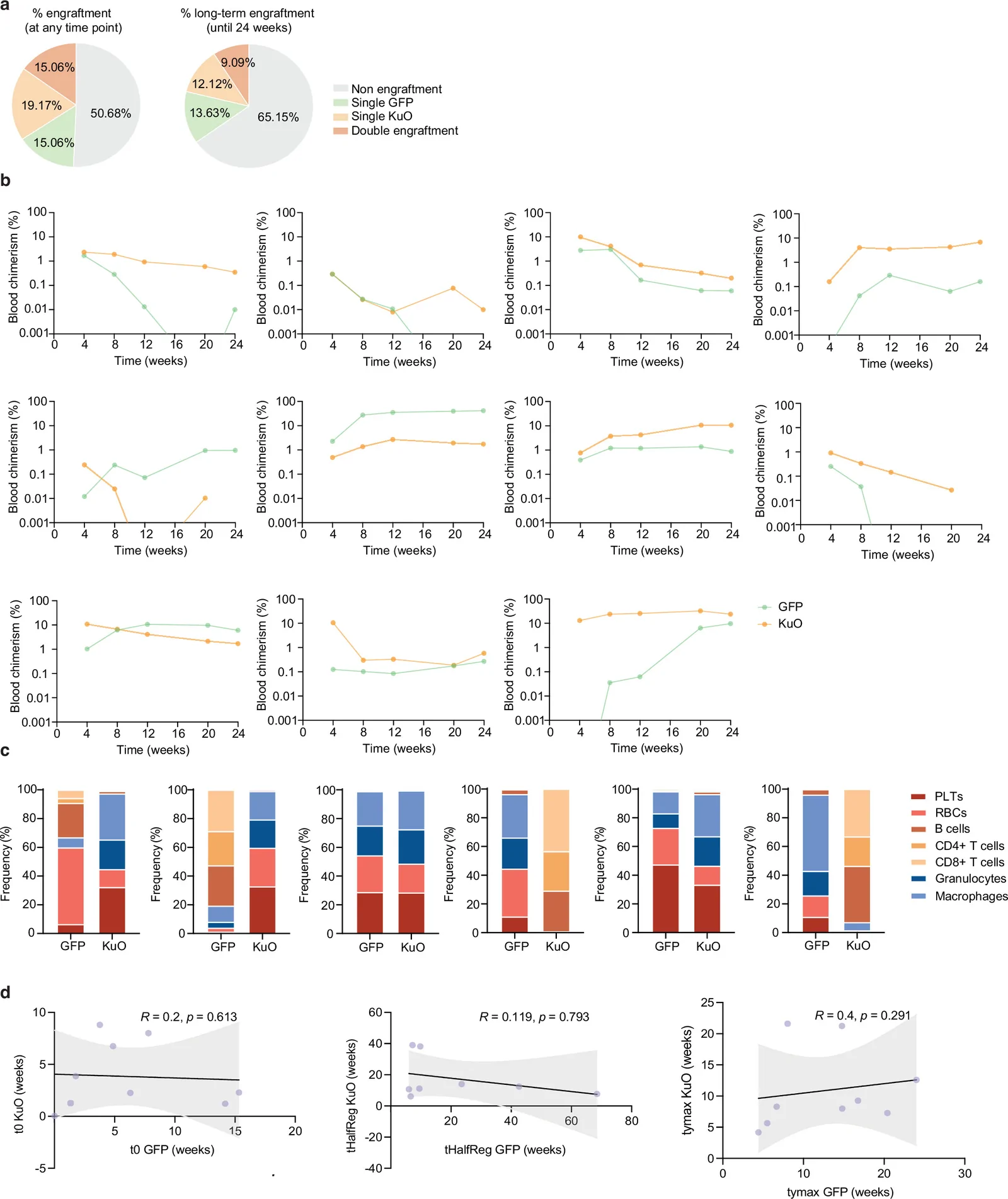
Functional outcomes from co-transplantation of two single LT-HSCs. **a**) Pie charts depicting the proportion of single and double engraftment following co-transplantation of individual UBC-GFP and KuO HSC clones. **b**) Peripheral blood donor chimerism over time post-transplantation in mice exhibiting double engraftment from both GFP+ and KuO+ clones. **c**) Lineage contribution in peripheral blood at endpoint analysis in mice with sustained double engraftment from both GFP^+^ and KuO^+^ clones. Only mice maintaining stable engraftment of both clones until the endpoint are shown. **d**) Spearman correlation analysis of reconstitution kinetics parameters (t0, tHalfReg and tymax) between co-engrafted GFP^+^ and KuO^+^ HSC clones. Source data

To corroborate the findings from this initial discovery cohort, we generated a larger independent validation cohort consisting of an additional 278 single LT-HSC transplants, in which single HSCs isolated from UBC–GFP mice and Kusabira Orange (KuO) mice^31^ were co-transplanted as a pair into recipient mice to reduce the total number of required recipient mice (Fig. 2d). Consistent with our initial dataset, we could readily observe heterogeneous clonal transplantation outcomes in the validation cohort with regards to the output of mature cells in the periphery and immature cells in the BM, as well as differential temporal patterns of blood cell production (Extended Data Figs. 5a–e and 6). After fitting all these clonal reconstitution patterns to a single humped function and applying our quantitative framework, we could interrogate the previously identified link between engraftment kinetics and HSC potency across this larger experimental cohort. This analysis unequivocally demonstrated the association between time-dependent reconstitution parameters and stemness features (Fig. 2e), supporting the hypothesis that blood reconstitution kinetics are a unifying metric closely tied to the apparently heterogeneous functional outputs observed following transplantation of highly purified single LT-HSCs.

### Lineage-biased HSC output correlates with reconstitution kinetics

**Fig. 3:**
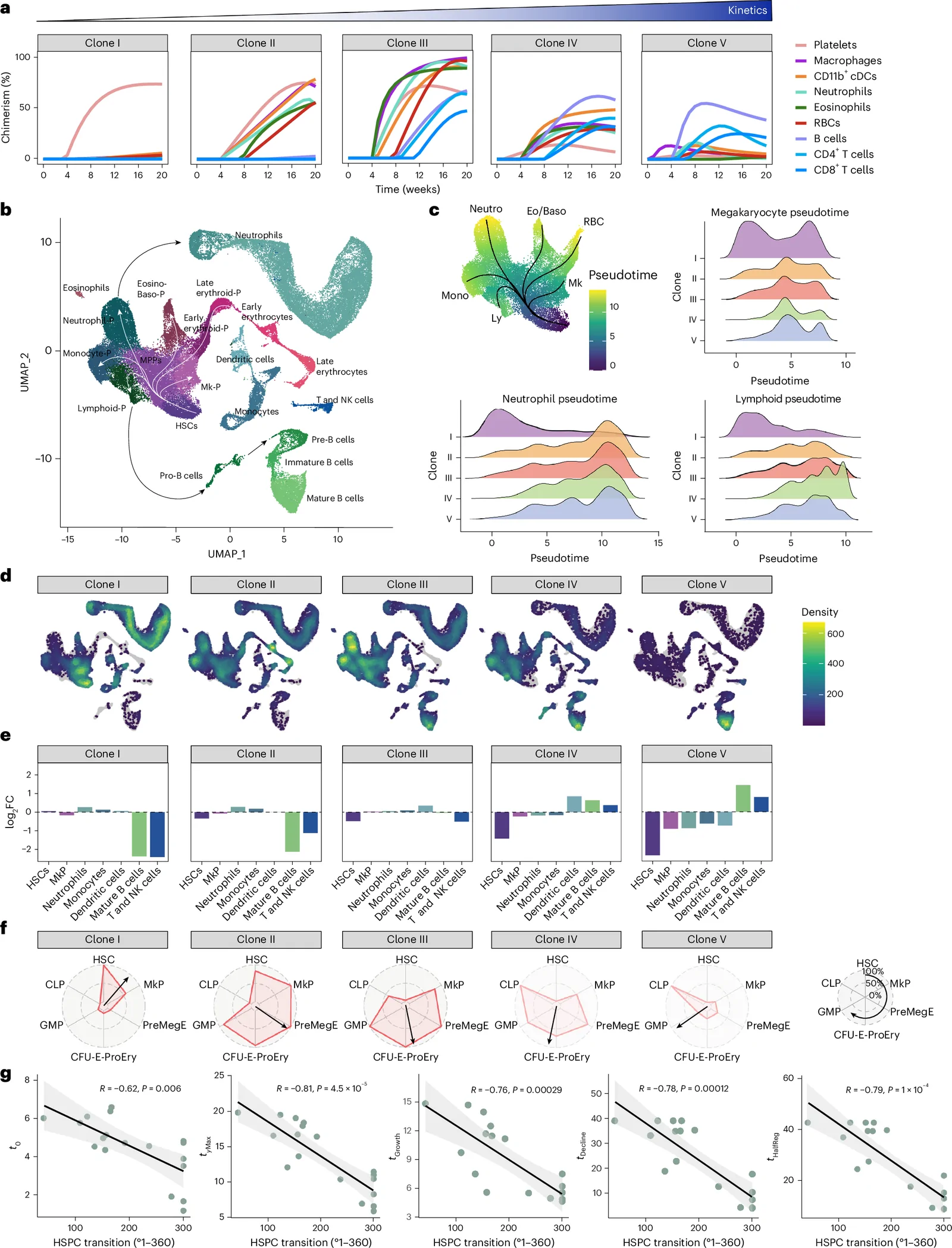
Kinetics of clonal reconstitution is associated with HSC lineage biases. **a**, PB donor chimerism for major blood cell lineages versus time in five exemplary clonal systems, ordered from slow (clone I) to fast (clone V), based on progressively increasing time delay *t*_0_. **b**, UMAP representation of scRNA-seq data from 20-week BM of clonal systems I–V, as well as two polyclonal controls which received 30 LT-HSCs. Inferred differentiation trajectories are illustrated by arrows. **c**, Pseudotime inference and density plots visualizing the distribution of cells for each clonally derived HSPC compartment over pseudotime. Top left: trajectories are indicated by arrows. Distribution of HSPCs at each stage of megakaryocyte, neutrophil and lymphoid lineage pseudotime development for each clonal system is shown in histograms. **d**, Relative cell-type density for each clonal system projected onto the UMAP from **b**. **e**, log_2_-fold change (log_2_FC) in cell type abundances from each clonally derived haematopoietic system relative to the mean of the polyclonal controls. **f**, HSPC transition clocks for systems I–V, derived from flow cytometric analysis of BM at 20 weeks post transplantation. Committed progenitors (MkP, PreMegE, CFU-E-ProEry, GMP and CLP) are ordered clockwise by Pearson correlation distance from HSCs based on their chimerism levels. The arrow indicates transition time around the clock, corresponding to the mean composition of the HSPC compartment. **g**, Spearman correlation of HSPC transition time versus kinetic parameters of all clonal systems from discovery cohort with detectable PB engraftment at time of BM collection. Spearman’s rho and significance are shown. 95% confidence intervals (CI) of the fitted regression line are highlighted in grey. cDC, conventional dendritic cell; RBC, red blood cell; P, progenitor; Mk, megakaryocyte; Eo, eosinophil; Baso, basophil; CFU-E-ProEry, colony-forming-unit erythroid proerythroblast; Mono, monocyte. Source data

Lineage-biased output from HSCs has been characterized by the disproportionate production of specific mature cell types and has been linked to stemness characteristics. Specifically, platelet- and myeloid-biased HSCs have been associated with high self-renewal capacities, whereas lymphoid-biased HSCs are linked to a decline in functional potency^6,7,11^. However, these lineage biases are typically defined by the cellular composition at a single arbitrary endpoint and do not account for the differing half-lives of mature blood cell types nor the wide spectrum in reconstitution kinetics of clonal systems that we have characterized. To explore the relationship between reconstitution kinetics and lineage-biased blood cell production, we first ranked all mature blood cell types by their first appearance in the PB of the discovery cohort, as defined by their mean delay parameter: *t*_0_ (Extended Data Fig. 4c). In line with previous reports, platelets were generated first, followed by myeloid cells and then lymphoid cells^6,7,11^. Although this sequence was consistent across most clonal systems, the delay between the onset of platelet generation and the subsequent generation of other lineages progressively increased from fast to slow reconstituting clonal systems. Fast clonal systems, with a low *t*_0_, showed an early burst in PB cell output across all lineages, followed by a rapid decline in chimerism (Fig. 3a and Extended Data Fig. 4b,d). As lymphoid cells exhibit longer half-lives and therefore persist longer after the exhaustion of their parental HSC compartment compared with myeloid, erythroid and platelet progeny, these fast clonal systems appear progressively more lymphoid-biased with the passage of time. By contrast, systems with higher *t*_0_ appeared myeloid-biased at earlier timepoints, then progressed to a more balanced mature cell output, sometimes with evidence of an eventual decay of the myeloid lineages at very late timepoints. Notably, systems with the highest *t*_0_ values did not generate myeloid or lymphoid cells during primary transplantation but demonstrated multipotency in secondary transplants. These data link accelerated HSC reconstitution kinetics with a shift from platelet to myeloid and lymphoid output and suggest that apparent lineage biases may be a function of the timepoint of analysis post transplantation rather than representing distinct hard-wired states. This is best exemplified by the observation that many HSC clones dynamically alter their lineage output across the experimental time course, meaning that they would fall into different lineage-skewed classification categories at different timepoints. A fact that is often overlooked when interpretation of transplantation outcome is predominantly focused on the very last timepoint analysed, since this is commonly perceived to have the greatest value with regards to durability of HSC output.

**Extended Data Fig. 7:**
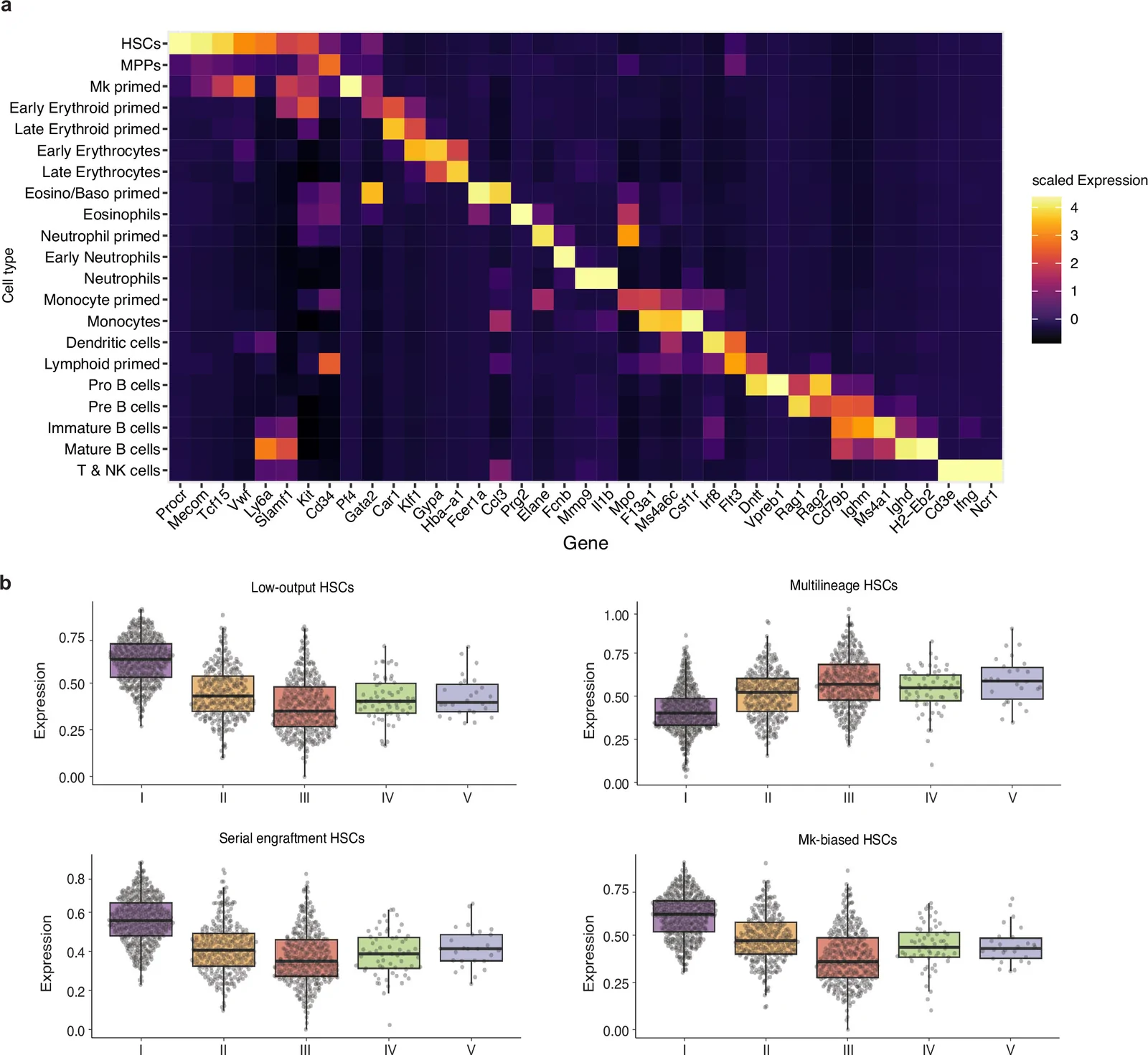
Single-cell transcriptomics analysis of clonally-derived hematopoietic systems. **a**) Gene expression heatmap illustrating major marker genes for each cell type. The color scale highlights the scaled average expression per gene in each cell type. **b**) Boxplots highlighting module scores of gene sets characteristic for low-output HSCs, multilineage HSCs, serial engrafting HSCs and Mk-biased HSCs in each HSC per clonally-derived system ordered by increasing blood cell repopulation and decreasing self-renewal. Gene sets are derived from8. Box plots: center line, median; box limits, first and third quartile; whiskers, smallest/largest value no further than 1.5*IQR from corresponding hinge. Abbreviations: HSC: hematopoietic stem cell; MPP: multipotent progenitor; Mk: megakaryocyte; Eosino: eosinophil; Baso: basophil; NK: natural killer cell. Source data

To gain a deeper understanding of how timepoint-resolved lineage-skewed output in the periphery relates to BM hematopoiesis, we performed droplet-based single-cell RNA sequencing (scRNA-seq) on BM progeny from five representative clonal systems (clones I–V) at week 20 post transplant (Fig. 3a,b), which encompassed the spectrum of slow to fast reconstitution that we observed in our discovery cohort (Fig. 1), as well as two polyclonal controls. This resulted in a clonally resolved map of 76,863 high-quality cells, covering all major haematopoietic cell types, encompassing different stages of differentiation: from the most immature HSCs, through lineage-committed progenitors, to more mature blood and immune cells (Fig. 3b and Extended Data Fig. 7a). Compositional and trajectory analyses of clonally derived cell states revealed that lineage-skewed blood production and variable reconstitution kinetics are reflected in the HSPC compartment at the time of collection (Fig. 3c–e). For instance, the slowest reconstituting clone (clone I), which predominantly produced platelets and only began generating myeloid cells at week 20, retained a high number of progeny in the most primitive HSC and MkP compartments, with modest occupancy of myeloid progenitor and mature cells. Lymphoid progenitors and maturing lymphoid cells were highly under-represented, reflecting the clonal system’s blood production at that moment in time (Fig. 3d,e). In line with this, daughter HSCs derived from slow reconstituting clones displayed gene expression signatures associated with low lineage output, high serial engraftment and megakaryocyte bias^8^, whereas daughter HSCs in faster clones showed a progressive increase in transcriptomic signatures of active multilineage HSCs (Extended Data Fig. 7b). Clonal systems with faster reconstitution kinetics demonstrated a progressive shift in relative abundance of transcriptomically defined cells. Thus, more rapid reconstitution kinetics correlated with a progressive decrease in the HSC and MkP compartments, accompanied by a transition from systems where the erythro-myeloid lineages dominated at the progenitor and mature cell level, to those where the lymphoid lineages were in the majority (Fig. 3d,e). These findings are consistent with a model where slowly differentiating HSC clones better regenerate the HSPC compartment, initially produce platelet- and myeloid-skewed progeny, and progressively transition to balanced and lymphoid-biased outcomes as their reconstitution kinetics increase and self-renewal capacity declines. However, these scRNA-seq data capture a snapshot of the lineage output of five representative clones at a single timepoint and does not cover the full spectrum of behaviour demonstrated across the entire cohort.

**Extended Data Fig. 8:**
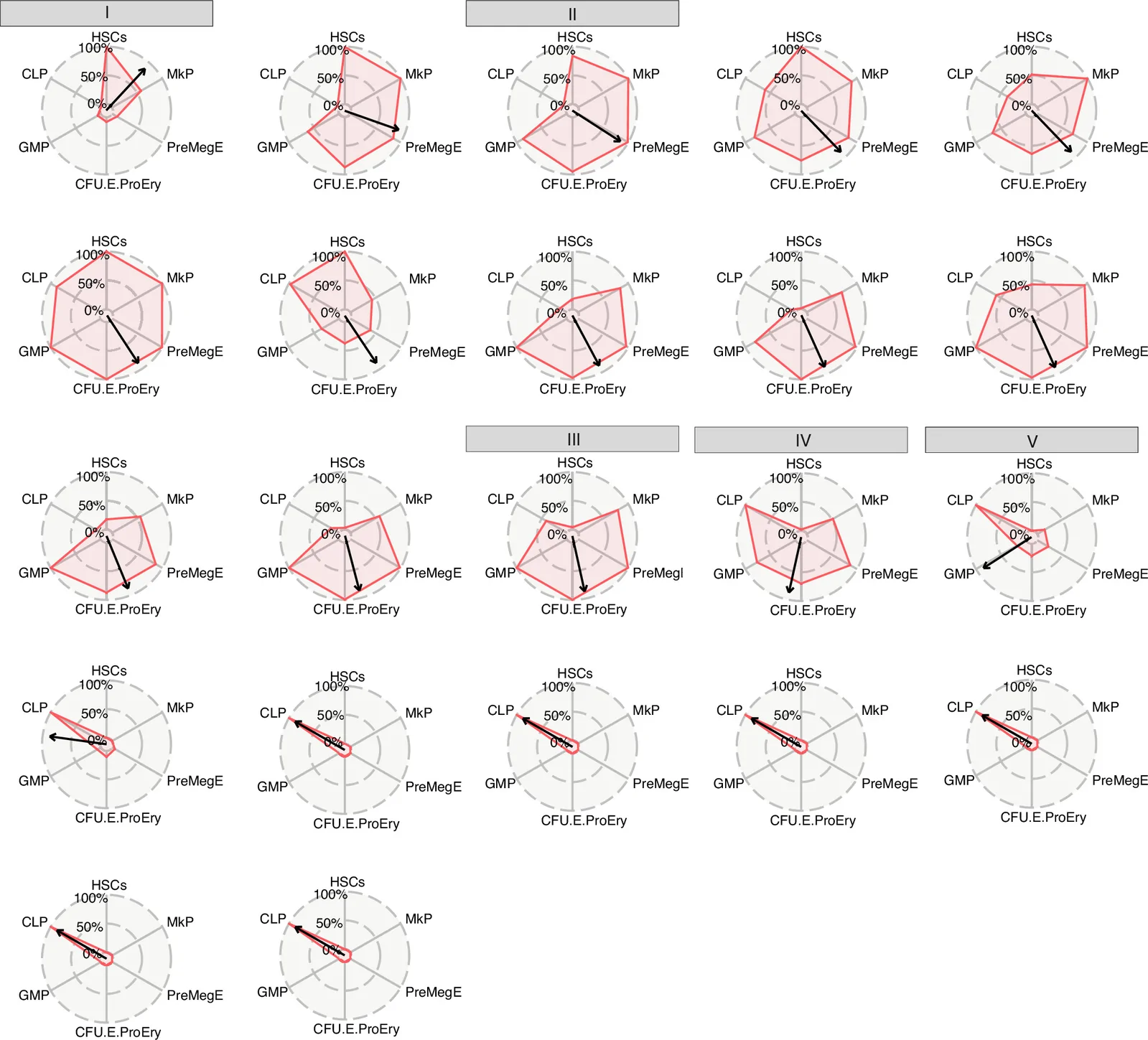
HSPC transition clocks for clonally-derived hematopoietic systems. Composition of HSPC compartments of clonally-derived systems from Fig. 1 that displayed sustained engraftment. Committed progenitors (MkP, PreMegE, CFU-E-ProEry, GMP and CLP) are ordered in a clockwise manner by Pearson correlation distance from HSCs based on donor chimerism of the progenitor compartments 20 weeks post-transplant (see illustration Fig. 3f). The arrow indicates the mean composition of the respective clonally-derived HSPC compartment and illustrates the current state of “HSPC transition”. Clonally-derived systems marked as I, II, III, IV and V correspond to the exemplary plots shown in Fig. 3f. Abbreviations: HSC: hematopoietic stem cell; MkP: megakaryocyte progenitor; PreMegE: pre-megakaryocyte-erythrocyte; CFU-E-ProEry: colony-forming-unit-erythrocyte-proerythroblast; GMP: granulocyte-monocyte progenitor; CLP: common lymphoid progenitor. Source data

To validate this putative link between reconstitution kinetics and lineage biases, we interrogated the clonally derived HSPC compartments from the deeply immunophenotyped discovery cohort. By ordering lineage-committed progenitors clockwise based on their Pearson correlation distance to HSCs, we generated clock-like representations of the HSPC compartment (Fig. 3f, right). Upon arranging all 22 clonal systems from the discovery cohort with sustained engraftment along this ‘clock’ framework, based on their mean HSPC composition—termed ‘HSPC transition time’—we were able to characterize systems ranging from those predominantly retaining HSCs (positioned closer to 12:00) to those with progressively more differentiated HSPC phenotypes (Extended Data Fig. 8). Importantly, we observed a strong anti-correlation between the kinetic parameters of individual clonal systems and the HSPC transition time (Fig. 3g), demonstrating that fast-reconstituting systems had transitioned further round this pseudotemporal clock than slow reconstituting systems at the timepoint of analysis. That is, slowly reconstituting systems retained a highly immature and megakaryocyte-primed HSPC compartment correlating with a restriction to platelet and myeloid generation, whereas faster systems showed a progressive shift to myeloid and lymphoid-primed HSPCs as production of mature cells skewed to balanced and then lymphoid outcomes. Through this transition, the primitive HSC compartment is progressively exhausted. Overall, our findings suggest that conventional categorical definitions of lineage biases and stemness are highly dependent on the timepoint of investigation and the underlying kinetics of the clonal system. By contrast, kinetics-based parameters provide an alternative approach for classifying clonal haematopoietic systems in a manner that is not subject to artefacts arising from the somewhat arbitrary timing of when post-transplantation analysis will be performed, relative to where on the reconstitution curve a given clone will be at that particular moment in time.

### Single-cell re-transplantation of clonally derived daughter HSCs demonstrate a unidirectional transition from slow to fast-reconstitution kinetics

**Fig. 4:**
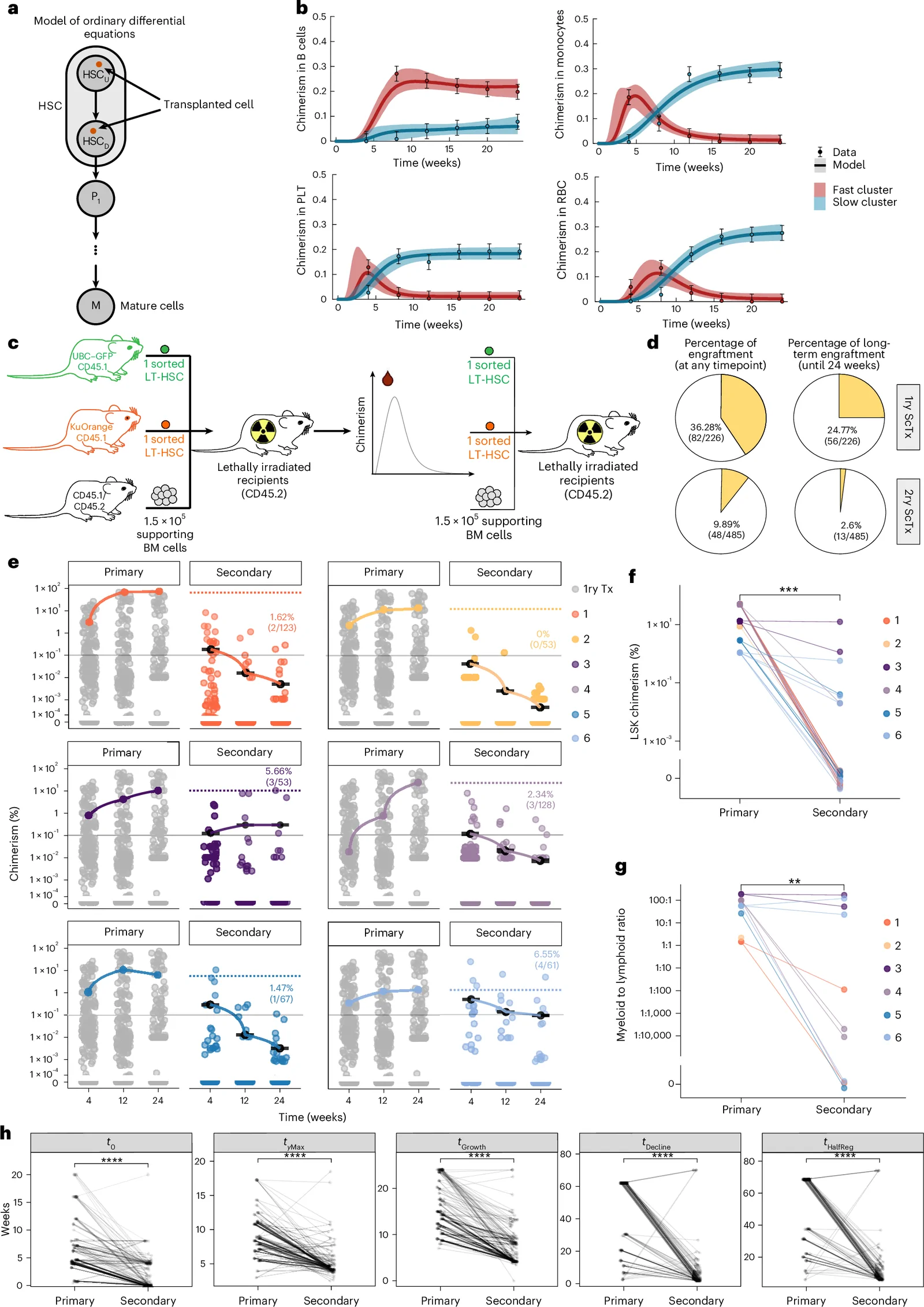
A shift from slow to fast kinetics in daughter HSCs is associated with a decline in functional potency. **a**, A schematic illustration of mathematical model in which HSCs can initiate differentiation from either an upstream (HSC_U_) or a downstream HSC subpopulation (HSC_D_). Differentiating HSCs go through a series of progenitor stages to produce mature cells (P_1_ to M). **b**, A comparison of experimental data to the fits derived from mathematical model for donor chimerism dynamics across B cell, platelet (PLT), monocyte and red blood cell (RBC) lineages in fast (red) and slow (blue) engrafting systems. Average values with pooled SEM and 90% confidence intervals (CI) for fits are shown. *n* = 8 clonal systems per group. **c**, A schematic representation of serial single cell transplantation experiments. A total of 485 LT-HSCs were purified from six separate primary recipients. Pairs of single GFP^+^ and KuOrange^+^ cells were co-transplanted into secondary recipients along with supporting BM cells. **d**, Percentage of all primary (1ry) and secondary (2ry) transplantations with successful engraftment (>0.1% donor chimerism in PB) at any timepoint post transplantation (left) or at 24 weeks post transplantation (right). **e**, A comparison of PB donor chimerism of the six original LT-HSC clones in primary transplantations versus all their respective daughter LT-HSCs following secondary transplantations, at the three indicated timepoints. Left: the primary clone (in colour) versus output of all other single LT-HSCs across the entire primary transplantation validation cohort (in grey) for comparison. Right: all daughter cell outcomes, in addition to the mean donor chimerism at each timepoint (black bar). Percentage and frequency of secondary clones giving rise to >0.1% donor chimerism in PB at each timepoint is indicated. Dashed lines indicate maximum level of PB chimerism achieved by corresponding primary clone. Primary clones are numbered 1–6, and secondary clones share the same colour code as indicated in the key. Primary transplants (grey dots) *n* = 221 from the validation cohort. Secondary transplants clone 1 *n* = 141, clone 2 *n* = 64, clone 3 *n* = 50, clone 4 *n* = 137, clone 5 *n* = 68 and clone 6 *n* = 64. The total number of clones that remained until the endpoint is shown in the graphs. **f**, Pairwise comparisons of donor HSPC (LSK) chimerism in BM at 24 weeks post transplantation in primary and secondary transplantations. Lines connect primary LT-HSC output to that of daughter LT-HSCs. *n* = 51 secondary transplantation clonal systems. ***P < 0.001, two-sided paired Wilcoxon signed-rank test. **g**, Pairwise comparison of ratio of myeloid to lymphoid PB chimerism at 24 weeks post transplantation in primary and secondary recipients. Lines connect primary LT-HSC output to that of daughter LT-HSCs. **P < 0.01, two-sided paired Wilcoxon signed-rank test. *n* = 13 clonal systems. **h**, Pairwise comparison of kinetics parameters (*t*_0_, *t*_*y*__Max_, *t*_Growth_, *t*_Decline_, *t*_HalfReg_) for each measured PB lineage in primary and secondary transplantations. Lines connect primary LT-HSC parameter for a given lineage to that of daughter LT-HSCs. **P < 0.01, ***P < 0.001, ****P < 0.0001, two-sided paired Wilcoxon test. ScTx, single-cell transplantation. Source data

Our data infer a hierarchical relationship between slow and fast-engrafting HSC clones, where slow-engrafting clones would be more primitive and therefore the potential precursor of clones with faster reconstitution kinetics. To investigate this hypothesis, we developed a mathematical model and tested it using our time-resolved chimerism data from the validation cohort. Initially, we used hierarchical clustering to categorize clonal systems as either fast- or slow-engrafting based on their blood reconstitution kinetics (Supplementary Fig. 1). We then assessed whether this dichotomy could be explained by a linear hierarchy within the HSC compartment, consisting of an upstream (slow) and downstream (fast) subcompartment (Fig. 4a). The model successfully reproduced the sequential production order of blood cell types post transplantation and captured the lineage biases associated with the distinct kinetics of blood production (Fig. 4b). Specifically, the slow system resulted from transplanted HSCs populating the upstream compartment, whereas the fast system was driven by HSCs populating predominantly the downstream compartment. These findings suggest that the experimental data fit a model describing transitions from slow- to fast-reconstituting clones in a linear hierarchy, associated with distinct kinetics of lineage contributions and declining functional potency. To validate this model in an experimental setting, we performed laborious serial single-cell transplantations, so that the post-engraftment output of individual daughter HSCs could be directly compared with that of the parent HSC. Such comparisons cannot be drawn by HSC barcoding approaches, because all daughter HSCs of a barcoded HSC will share the exact same barcode and will therefore be indistinguishable from each other. We selected six primary recipients of single HSCs from the validation cohort which showed robust chimerism in the HSPC compartment at the 24-week post-transplantation experimental endpoint and which demonstrated slow- to intermediate-reconstitution kinetics. We collected single HSCs from these donors and re-transplanted a total of 525 single daughter cells into secondary recipients, which represented the majority of the HSC reserve that we could isolate from the primary recipient mice (Fig. 4c). When considering the entire secondary transplantation cohort, the overall percentage of daughter HSCs with detectable engraftment in PB at any timepoint declined from 36.3% in primary recipients to 9.9%, in secondary recipients, whereas the percentage of clones with LT engraftment capacity dropped even more dramatically, from 24.8% to 2.6%, (Fig. 4d). This generational decline in functional potency was even more pronounced when performing a pairwise analysis between individual HSCs and their daughter HSCs, where almost all progeny demonstrated a steep decline in reconstitution of both mature cells and the HSPC compartment compared with the parental clone (Fig. 4e–g). Remarkably, all but one of the more than 500 re-transplanted HSCs (99.8%) exhibited decreased chimerism levels compared with their parent HSC at week 24 post transplant (Fig. 4e), representing a decline in functional potency in virtually all secondary HSC clones and suggesting that full self-renewal is a very rare event in the context of transplantation. Although most daughter HSCs demonstrated reduced potency compared with their precursor clone, it is conceivable that the sum of the functional potency of all daughter HSCs may equal or exceed that of the parent.

**Extended Data Fig. 9:**
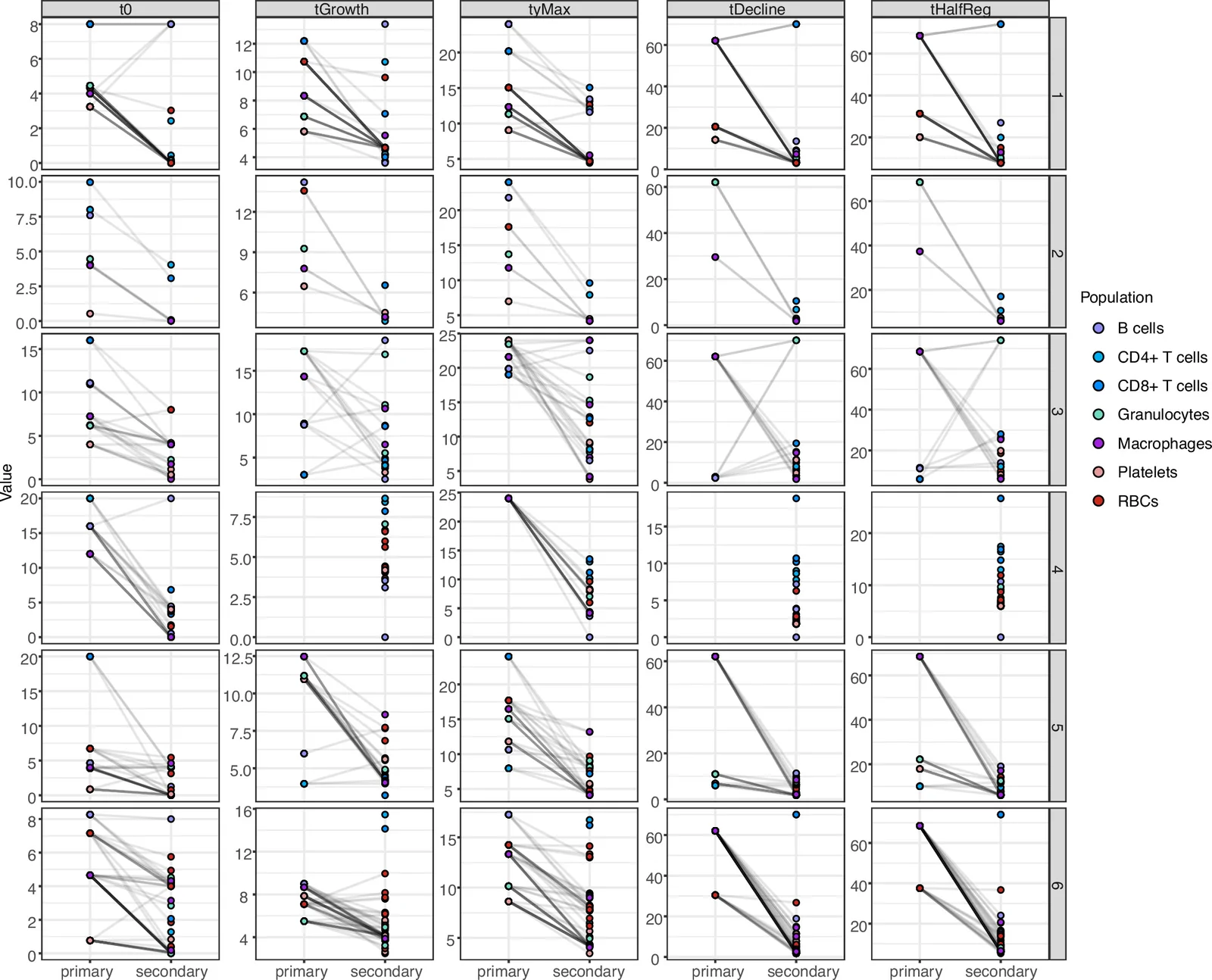
HSCs transition from slow to fast reconstitution kinetics upon secondary transplantation. Paired dot-plots of time-dependent kinetic parameters highlighting changes in blood cell replenishment between transplanted parent and daughter HSCs per clone. Cell populations are color-coded. Each row represents a paired analysis between parent (primary) and corresponding daughter (secondary) HSCs. Abbreviations: RBC: red blood cell. Source data

To investigate whether these serial transplantation data conformed to our kinetics-based hierarchy model (Fig. 4a,b), we again fitted our data to a single humped function, derived kinetic parameters for both parent- and daughter-derived clonal systems and subsequently performed a pairwise analysis between related clones. Importantly, daughter clonal systems exhibited significantly accelerated kinetics across all parameters compared to their respective parents (Fig. 4h and Extended Data Fig. 9). Notably, almost all daughter stem cells also unidirectionally shifted from myeloid-biased to more lymphoid-biased blood production, consistent with our previous data linking faster reconstitution kinetics to this shift in lineage output (Fig. 4g). In very rare cases, daughter clones maintained reconstitution kinetics-based parameters compared with their parents, which was linked to high self-renewal of the HSPC compartment and the maintenance of myeloid-to-lymphoid ratio in secondary transplantation endpoint analyses (Fig. 4f–h and Extended Data Fig. 9). These findings validate the prediction that slow-engrafting clones unidirectionally give rise to faster engrafting HSC clones, associated with a progressive change in lineage output and loss of functional potency.

### Cellular competition between progeny of slow and fast-engrafting clones contributes to extrinsic regulation of lineage-biased HSC output

**Fig. 5:**
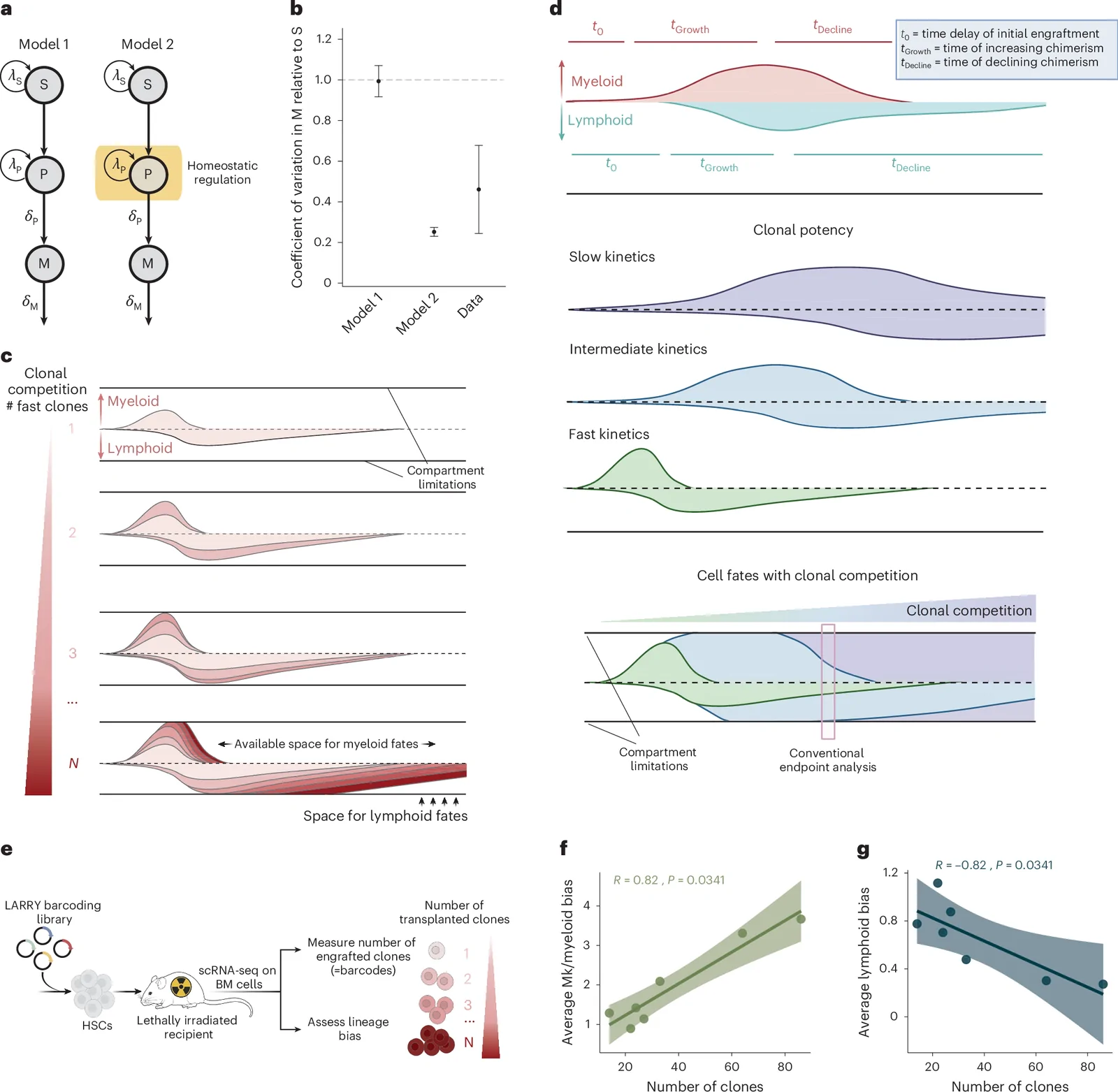
Increased cellular competition modulates HSC lineage biases. **a**, An illustration of mathematical models describing the process of HSC haematopoietic differentiation in the absence (model 1) or presence of homeostatic regulation (model 2). Curved arrows depict proliferation within stem (S) and progenitor (P) populations, whereas straight arrows indicate differentiation towards mature cells (M). *λ* = proliferation rate; *δ* = differentiation rate. **b**, Coefficient of variation in mature cells (M) relative to stem cells (S) predicted by model 1 and 2, shown in comparison with experimental data from validation cohort. Error bars indicated s.d. *n* = 129 clones. **c**, A schematic illustration of how increasing the number of competing fast clones would hypothetically result in a higher proportion of myeloid-skewed outputs as a result of the difference in generation and decay times between myeloid and lymphoid cells. **d**, A schematic illustration of how competition between HSC clones with different engraftment kinetics would amplify myeloid-skewed output in slow clones. Top: no competition, where clones are free to realize myeloid and lymphoid fates. Bottom: illustrations of inter-clonal competition. **e**, A schematic representation of LARRY barcoding experiments. Barcoded HSCs were transplanted into lethally irradiated recipient mice. A total of 16 weeks post transplant, BM cells were isolated and subjected to scRNA-seq analysis. Lineage biases and the number of transplanted clones (barcodes) were extracted from the scRNA-seq data. **f–g**, Two-sided Spearman correlation analysis between the number of transplanted clones and the average of Mk/myeloid bias (**f**) or the average of lymphoid bias (**g**). Spearman’s rho and significance are indicated. Error bands represent the 95% confidence intervals (CI) of the fitted regression line. Schematics in **c**–**e** created in BioRender; Milsom, M. http://biorender.com/gwm5s48 (2026). Source data

**Extended Data Fig. 10:**
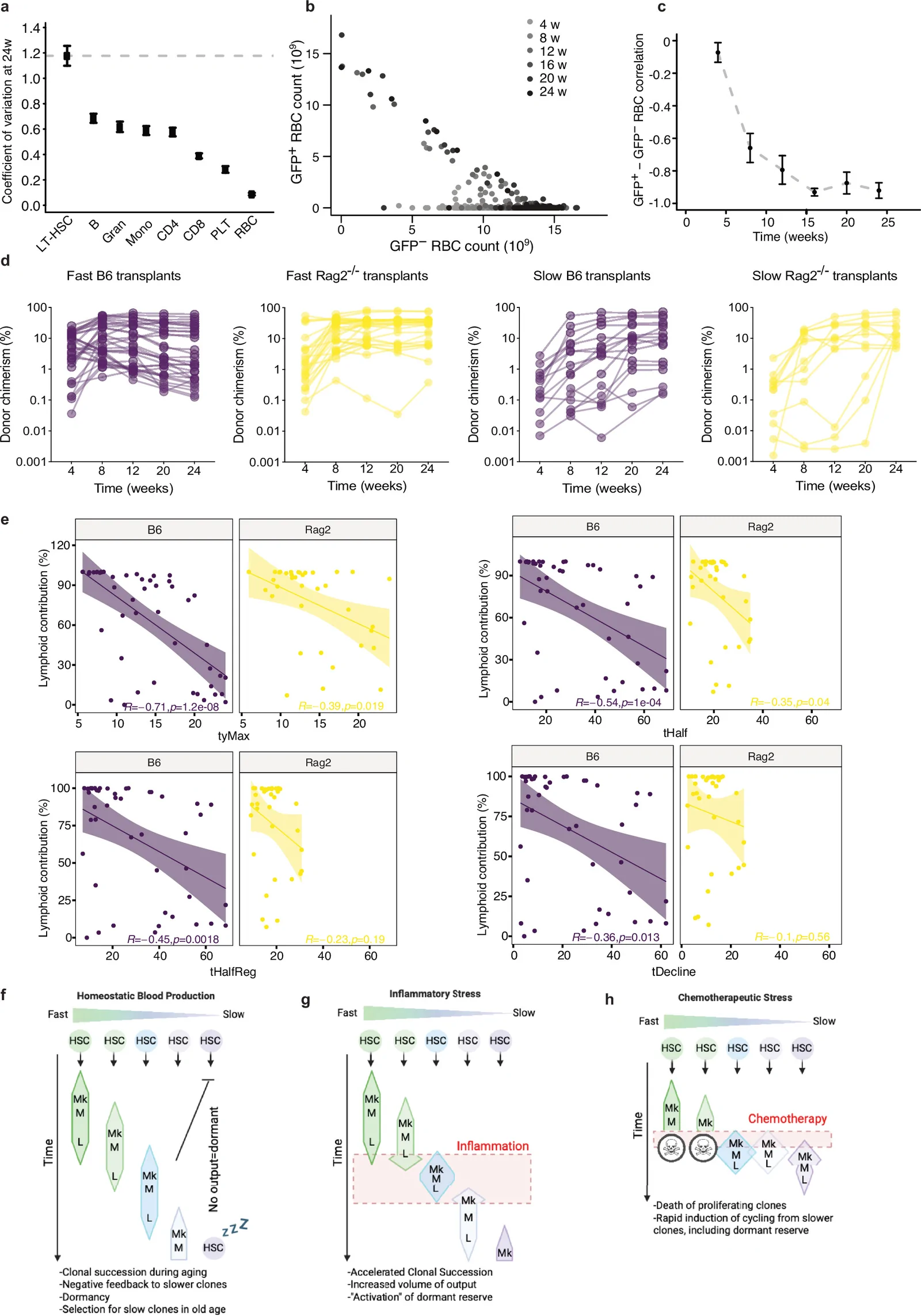
Cell-extrinsic mechanisms modulate HSC lineage biases. **a**) Coefficient of variation of bone marrow LT-HSCs and peripheral blood B cells (B), granulocytes (Gran), monocytes (Mono), CD4 + T cells (CD4), CD8 + T cells (CD8), platelets (PLT) and red blood cells (RBC) at 24 weeks post-transplantation. **b**) Correlation analysis between the absolute counts of single HSC-derived GFP+ and non-clonal GFP- RBCs measured in the peripheral blood at 4, 8, 12, 16, 20 and 24 weeks post-transplantation. **c**) Correlation coefficient extracted from (**b**) at different time points post-transplantation. **d**) Peripheral blood donor chimerism over time post-transplantation comparing fast (t0 < 6)- and slow (t0 > 6)-reconstituting clones transplanted in the B6 or Rag2-/- systems. **e**) Spearman correlation between the average kinetics parameters tyMax, tHalf, tHalfReg and tDecline of a single HSC and its percentage of lymphoid contribution in peripheral blood at 24 weeks post-transplantation, in B6 versus Rag2-/- hosts. Spearman’s Rho and significance are indicated. Abbreviations: LT-HSC: long-term hematopoietic stem cell; B: B cells; Gran: granulocyte; Mono: monocyte; CD4: CD4 + T cell; CD8: CD8 + T cell; PLT: platelet; RBC: red blood cell; w: week; B6: C57BL/6 J mouse model; Rag2: homozygous knock-out of the Recombination Activating Gene 2. **f-g**) Potential impact of new model on various hematopoietic phenotypes. The passage of time is indicated from top to bottom of the figure sub-panels and the horizontal width of the bars under each clone indicates the volume of mature blood cell production from that clone at that moment in time. Mk: Megakaryocytic output, M: Myeloid output, L: Lymphoid output. **f**) Under homoeostatic conditions, we would hypothesize that sequential clonal succession is a consequence of the differential kinetics of production of mature blood cells from fast and slow HSC clones, in response to moderate demand produced by the turnover of mature blood cells with the passage of time. The mature cell output of the preceding clones would restrict output from slower clones as predicted by our compartment size restriction model. Overall, this would result in a gradual clonal contraction during aging, since fast clones would be exhausted earlier in life and intermediate clones would progress into become fast clones. This clonal collapse may be an underlying driver of age-associated clonal hematopoiesis. HSCs with very slow kinetics of output may remain quiescent (dormant) for long periods of time and would possess the highest functional potency in transplantation assays. **g**) In response to intermediate stress, such as infection or inflammation, increased demand for mature blood cell production would be hypothesized to accelerate the kinetics of clonal succession, increasing the rate of contraction of the HSC pool with the passage of time. This would result in earlier demand for mature cell output from slower (dormant) clones. Repeated exposure to such challenge would be predicted to gradually “activate” an increasing proportion of slow, dormant HSCs into active proliferation and differentiation. Clones which are unresponsive to such stimulus (for example through acquisition of mutations that compromise the clone’s ability to produce mature blood cells, such as DNMT3A loss of function) may have a strong selective advantage under such conditions. **h**) We would hypothesize that chemotherapeutic treatment would result in the preferential induction of cell death in clones that are actively dividing at the time of treatment, which should correspond to faster clones. The depletion of the HSPC compartment from faster clones would result in a massively increased demand for mature cell blood production from the remaining slower clones. This will dramatically accelerate the process of clonal succession and contraction. Repeated exposure to such challenge would potentially exhaust the dormant reserve of HSCs, leading to bone marrow failure. Clones which have acquired resistance to such chemotherapeutic challenge (for example through the acquisition of loss of function mutations in TP53 or PPM1D) would have a strong selection advantage under such conditions. Diagrams in **f**–**h** created in BioRender; Milsom, M. https://biorender.com/gwm5s48 (2026). Source data

Our paired daughter cell experiments support the concept of cell intrinsic inheritance of functional properties, as we observe a generational acceleration in engraftment kinetics alongside decreased self-renewal. However, in our studies, single-purified HSCs are not transplanted in isolation; rather, they are co-transplanted with additional supportive BM. Thus, overall blood cell production in an individual recipient arises from the combined output of several clones encompassing a spectrum of differing reconstitution kinetics. Given the fact that transplanted mice reconstitute the various mature blood lineages within a specific range of values, it follows that these co-existing clonal systems may interact to regulate their overall output at a lineage-specific level. To explore this concept, we exploited the fact that both the total number of BM LT-HSCs (at 24 weeks) and the mature progeny generated from each clonal system vary from mouse to mouse. We used mathematical modelling to simulate the process via which LT-HSC output might be regulated by the levels of more mature cells, devising two mutually exclusive models: model 1 lacks feedback regulation, whereas model 2 incorporates feedback regulation where the sizes for mature cell compartments are set by negative feedback (Fig. 5a). In model 1, the variation in total HSC numbers, as measured by the coefficient of variation, was passed on to the variation in mature cell numbers, whereas in model 2, feedback regulation strongly reduced the variation in the cellularity of mature blood populations (Fig. 5b). We found that model 2 aligned much better with our experimental data compared with model 1, suggesting that, despite variability in contributions from individual HSC-derived haematopoietic systems, the production of mature haematopoietic cells remains tightly constrained by homeostatic feedback mechanisms that enforce strict compartment size limits (Extended Data Fig. 10a). This observation is in line with the requirement to sustain mature blood cell levels within certain constraints to facilitate survival of the organism. Interestingly, HSCs exhibited a significantly higher coefficient of variation than mature cells, suggesting that the compartment size limit, if present at all, is much weaker for HSCs or has not been reached in the setting where only a small number of input stem cells have been transplanted. Given the compartment size restriction in mature cell populations, we reasoned that the replenishment of mature blood populations by single HSC clones might be influenced by competing HSC clones. If correct, this hypothesis would predict that fast-engrafting clones would rapidly fill up cellular compartments to their limit, whereas slowly engrafting clones would only be able to contribute to mature cell production once the levels of mature blood cells had declined below this limit, due to exhaustion of fast-engrafting HSCs and their progeny. To investigate this hypothesis, we quantified the absolute levels of mature blood cells produced by the transplanted single HSCs versus those derived from the co-transplanted supportive BM in the same mice. Consistent with our hypothesis, we observed a strict inverse correlation between absolute numbers of mature cells generated by the single LT-HSCs and the co-transplanted supporting BM (Extended Data Fig. 10b). Notably, the competition between clonal offspring and supportive BM was low at the beginning of transplantation and gradually increased over time, eventually plateauing as the maximum compartment size limit was reached (Extended Data Fig. 10c). Collectively, these experimental data support the model that feedback regulation restricts the compartment size of mature blood populations and raise the possibility that clonal competition between slow- and fast-reconstituting clones might contribute to the establishment of lineage biases in a cell-extrinsic manner.

Based on this model, we hypothesized that in the absence of competition, HSCs would be able to realize both myeloid and lymphoid potentials, with the output of these cell types only being separated by the different time interval it takes to generate a mature myeloid cell, versus a mature lymphoid cell (Fig. 5c). Conversely, if one were to increase the number of clones which simultaneously produce mature progeny, then one would introduce competition to fill mature cells to their compartment size limits. Because myeloid cells generally have a much shorter half-life than lymphoid cells, then myeloid cells would decline below the compartment size limit ahead of the lymphoid cells, once the parent HSC had exhausted, meaning that there would be ‘space’ for other clones to produce myeloid progeny ahead of such space becoming available for lymphoid output (Fig. 5c). This would have the effect of increasing the number of clones with myeloid-skewed outputs with increasing competition. We would predict that this effect would become amplified further if one were to consider competing clones that engraft with different kinetics, where slow-engrafting clones would additionally have to wait for the progeny of more rapidly engrafting clones to be turned over before they would have space to generate differentiated cells (Fig. 5d). To test this hypothetical link between clonal competition and lineage output, we first assessed whether increasing clonal competition between HSCs would result in the predicted increase in the proportion of clones with a myeloid-skewed output. To these ends, pools of purified HSCs were transduced with the LARRY library of lentiviral-encoded barcodes^8^, before bulk transplantation into recipient mice (Fig. 5e). Using the unique clonal barcodes, the number of engrafting HSC clones was then calculated for each recipient and was compared with the average level of megakaryocyte/myeloid or lymphoid bias across all clones. As predicted by our competition model, we saw a strong positive correlation between the number of engrafting HSC clones and the proportional realization of megakaryocytic/myeloid fates (Fig. 5f) and an anti-correlation with the proportion of lymphoid-skewed outputs (Fig. 5g). This provides one line of evidence that cell extrinsic competition can modulate lineage-skewed output from multipotent HSCs.

**Fig. 6:**
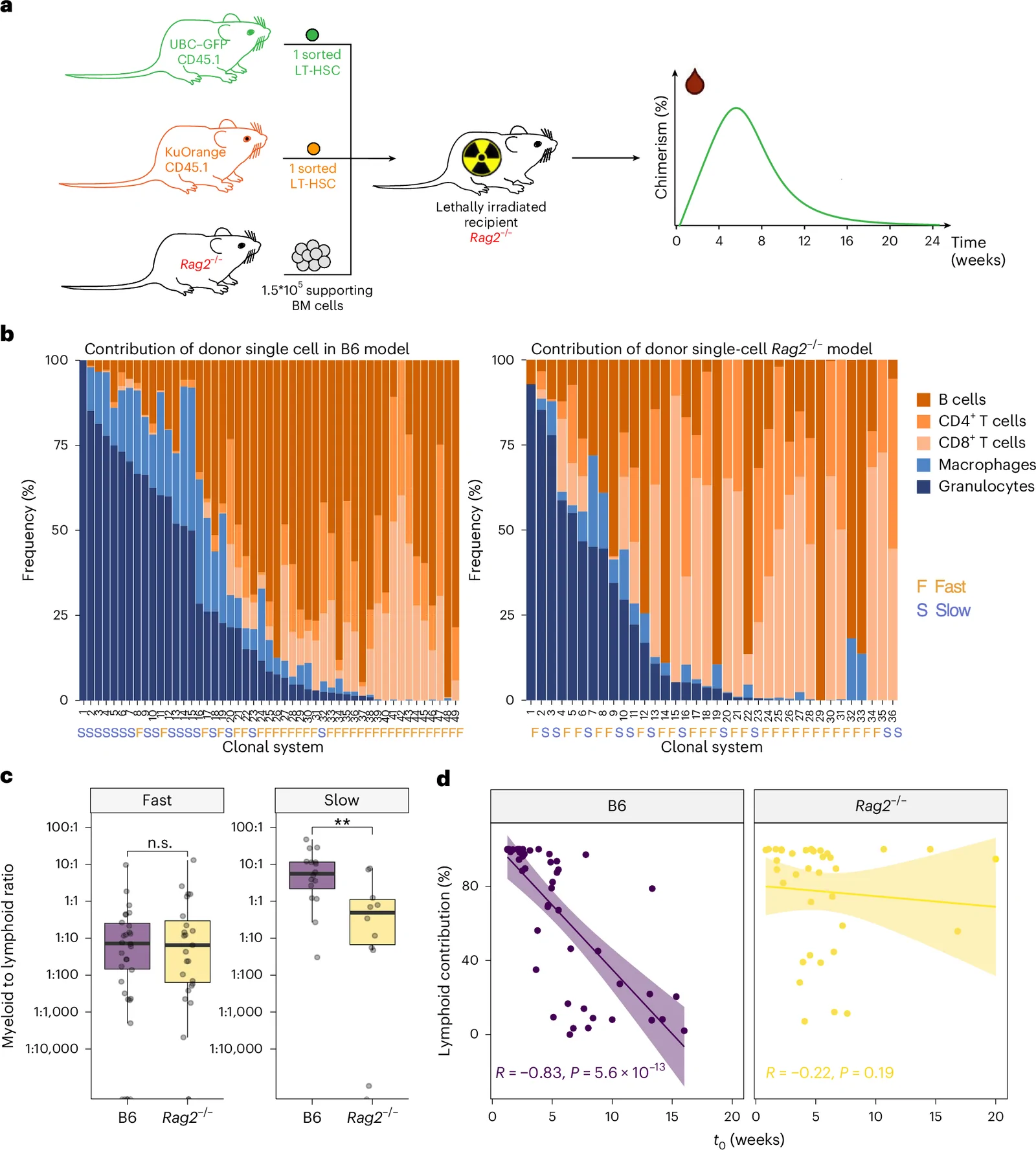
Cell-extrinsic feedback from lymphoid progeny of competing clones promotes myeloid-skewed output from slow-engrafting HSCs. **a**, A schematic illustration of single-cell transplantations into *Rag2*^−/−^recipients using *Rag2*^−/−^-supportive BM cells. **b**, Frequency of PB populations produced by the single transplanted HSC at 24 weeks post transplantation using regular C57BL/6 (B6)-supportive BM and recipients (left) or using *Rag2*^−/−^-supportive BM and recipients (right). Cell types are colour coded. Slow (S) and fast (F) clones are indicated. **c**, The ratio of myeloid-to-lymphoid cell frequency in PB at 24 weeks post transplantation, comparing fast (*t*_0_ < 6)- and slow (*t*_0_ > 6)-reconstituting clones transplanted in the B6 or *Rag2*^−/−^ systems. Not significant (n.s.) = *P* > 0.05, **P < 0.01, two-sided Wilcoxon rank-sum test. Box plots show the centre line, median; box limits, first and third quartiles; whiskers, smallest and largest values within 1.5× interquartile range from the hinges. B6 transplants *n* = 48 from co-transplanted clones in the validation cohort (*n* = 16 slow, *n* = 32 fast), *Rag2*^−/−^ transplants *n* = 36 (*n* = 11 slow, *n* = 25 fast). **d**, Two-sided Spearman correlation analysis between the average time delay (*t*_0_) in reconstitution of a single HSC and its percentage of lymphoid contribution in PB at 24 weeks post transplantation, in B6 versus *Rag2*^−/−^ hosts. Each dot represents a single HSC transplantation. Spearman’s rho and significance are indicated. The error bands represent the 95% confidence intervals (CI) of the fitted regression line. *n* = 36 *Rag2*^−/−^ or *n* = 48 B6 clonal systems. Source data

To further interrogate the prediction that competition between fast and slow clones drives lineage-skewed output, we made use of the congenic *Rag2* knockout mouse (*Rag2*^−/−^) model^32^, whose HSCs are capable of erythro-myeloid reconstitution but lack the ability to produce mature lymphoid cells (Fig. 6a). We transplanted a total of 154 single *Rag2*-wild-type UBC–GFP or KuO LT-HSCs, together with *Rag2*^−/−^ supporting BM cells, into *Rag2*^−/−^ recipient mice. In this setting, neither the supporting BM nor the residual recipient hematopoiesis could contribute towards filling the mature lymphoid compartment size limit. We then measured the clonal lineage output and reconstitution kinetics of the wild-type HSCs and compared this with wild-type LT-HSCs transplanted into wild-type recipients along with wild-type supporting BM (Fig. 6a). The level of donor chimerism was generally equivalent across the two different recipient groups, suggesting that the LT reconstitution potential of the individual transplanted HSCs was not radically altered in the new host model (Extended Data Fig. 10d). To investigate potential extrinsic effects on the relationship between engraftment kinetics and lineage output, we broadly subsegregated clonal transplantation outcomes into slow and fast engrafting based on the *t*_0_ value for each system. Interestingly, we observed both fast and slow-engrafting clones in both experimental arms, suggesting that kinetic parameters are independent of the lineage output of the co-transplanted competitor cells. However, slow-engrafting clones demonstrated an altered lineage output dependent on the cell extrinsic environment (Fig. 6b,c). Thus, slow clones co-transplanted with lymphoid-proficient competitors displayed a pronounced myeloid bias as observed in the preceding experiments, whereas those transplanted into a *Rag2*^−/−^ haematopoietic system did not exhibit the same lineage skewing, breaking the inverse correlation between the degree of lymphoid output and kinetic-based parameters, such as *t*_0_ (Fig. 6d). In contrast to the differential output of slow clones dependent on which recipient was used, fast clones were not altered in their lineage bias, in line with the notion that these clones fill up compartment sizes first and are therefore less affected by competition feedback from already established mature cells (Fig. 6c). These findings demonstrate that the link between kinetic-based reconstitution parameters and apparent myeloid bias can be uncoupled by modulating the capacity of competitor HSPCs to contribute towards filling mature lymphoid lineages (Fig. 6d and Extended Data Fig. 10e). Taken together with the results from the barcoding experiments (Fig. 5e–g), these data provide compelling evidence that apparent intrinsic lineage biases are in fact highly dependent on cell extrinsic regulation, resulting from a competition between slow and fast-engrafting HSC clones to saturate the production of mature blood cells until lineage-specific compartment sizes are filled (Supplementary Video 1).

## Discussion

Numerous studies have used single-cell transplantations to describe functional heterogeneity within the primitive HSC pool^5–7,10,33–35^, including at the level of reconstitution kinetics^30^. However, there has been a lack of clarity regarding whether these diverse phenotypic outcomes represent discrete intermediates in a branched hierarchy of the most primitive HSCs or rather cell states aligned along a linear trajectory. This has been particularly confusing with regards to the relationship between HSC lineage bias and multipotency, as the concept of progressive lineage commitment does not seem to be compatible with a model where the most primitive HSCs demonstrate an intrinsic lineage bias, yet generate HSC progeny which are more permissive in the spectrum of cell types they can produce. By dispensing with the classical subclassification of HSCs according to somewhat arbitrary thresholds and timepoints, our alignment of functionally heterogeneous outcomes along a continuous kinetics-based scale provides a framework to assign potency based on self-renewal capacity. This allowed us to synthesize a rationale to explain why the most potent HSCs predominantly produce platelet and myeloid progeny once they first contribute to mature blood cell production, despite being multipotent^11–13^. Indeed, the revelation that lineage-skewed output from slower engrafting clones is driven by extrinsic feedback from the mature progeny of more rapidly engrafting competitor clones, links variability in clonal output to systemic demand for mature blood cells. This insight has important implications for our understanding of both normal and diseased hematopoiesis and also helps explain why data demonstrating a concrete molecular basis for such intrinsic biases in the HSC compartment have not yet emerged.

A kinetics-based functional hierarchy aligns well with other transplantation-based studies that clearly support successive waves of HSC clones contributing to mature blood cell production where sustained engraftment and regeneration of the HSC pool was supported by slow or low-output clones, including barcoding approaches in murine and primate HSCs^8,36^ and analyses of human engraftment based on retroviral integration sites^37,38^. However, it remains unclear how this relates to the setting of native hematopoiesis, where the transition time from primitive HSCs through to mature blood cells is longer and challenging to measure in an experimental setting in the absence of stimuli that provoke emergency hematopoiesis^39,40^. Nonetheless, it is tempting to speculate that slow-engrafting HSC clones may equate to so-called dormant HSCs, which maintain a state of LT quiescence during native unperturbed hematopoiesis^35^. Certainly, both cell types appear to represent a subset of highly potent HSCs, which have an inherent capacity to restrict their output of progeny, either in the face of pro-proliferative stimuli acting over the course of long time periods in the native niche or in a myeloablated niche. A direct comparative analysis is restricted by the fact that both cell types can only be identified retrospectively, but it would be interesting to understand the underlying molecular basis for this restricted output, as well as how and why such HSCs eventually overcome this restriction following a temporal delay.

One setting of native hematopoiesis where our findings may be of immediate relevance is the accumulation of myeloid-biased HSCs during ageing, which has been attributed as the root cause of a number of age-associated pathological processes ranging from the evolution of myeloid malignancies to immune dysfunction^41–43^. One could extrapolate from our data that ageing may result in a progressive accumulation of multipotent HSCs with delayed kinetics and therefore appear myeloid-biased following transplantation. Perhaps such a phenotype might even be selected for during ageing, because clones that actively contribute to blood formation will be preferentially lost from the HSC pool^44,45^. This hypothesis aligns with the enrichment of so-called latent HSCs within aged murine BM, which demonstrate low-output myeloid-skewed production in primary recipients, but give rise to robust multilineage reconstitution upon secondary transplantation^34^ (Extended Data Fig. 10f–h).

Collectively, our study identifies reconstitution kinetics as a unifying and continuous metric for classifying primitive HSCs according to their functional potential and provides an underlying rationale for lineage-skewed output from these multipotent cells. Furthermore, the kinetics-based principles outlined in this manuscript may have broad relevance for understanding the establishment and remodelling of clonal mosaicism during the development and ageing of other regenerating tissues throughout the body.

## Methods

### Animal experiments

All animal experiments were approved by the Animal Care and Use Committees of the German Regierungspräsidium Karlsruhe für Tierschutz und Arzneimittelüberwachung (Karlsruhe, Germany) under TVAs G-41/19 and G-50/17. Mice were maintained in individually ventilated cages under specific pathogen-free conditions at the German Cancer Research Center (DKFZ, Heidelberg), with ad libitum access to water and food (22 ± 2 °C, 45–65% humidity, 12 h light–dark cycle). Wild-type mice (C57BL/6J) were obtained from Janvier Laboratories. Female C67BL/6J recipient mice were 8–12 weeks old when experiments were initiated. UBC–GFP and KuOrange (KuO) male or female mice were used as donors for transplantation experiments. Both male and female *Rag2*^−/−^ were used as donors and recipients and recipients were 8–12-weeks-old when transplanted. All three mouse lines were bred in-house.

### Single-cell transplantations

CD45.2^+^ C57BL/6J mice were lethally irradiated with two rounds of 500 Rad. A total of 24 h later, the mice were transplanted via intravenous injection with a single CD45.1^+^ LT-HSC (EPCR^hi^, CD34^−^, CD150^+^, CD48^−^ and LSK) derived from a transgenic CD45.1^+^ UBC–GFP donor mouse, together with 1.5 × 10^5^ WT CD45.1^+^/CD45.2^+^-supportive whole BM cells. In a second group of experiments, co-transplantations of single CD45.1^+^ UBC–GFP LT-HSC plus single CD45.1^+^ KuO LT-HSC together with 1.5 × 10^5^ WT CD45.1^+^/CD45.2^+^-supportive whole BM cells were performed. Co-transplantation were also performed in combination with *Rag2*^−/−^-supportive BM into *Rag2*^−/−^-recipient mice. Engraftment potential was assessed at 4, 8, 12, 16 and 20 weeks and, in some cases, at 24 weeks post transplantation in PB cells, and at 20 or 24 weeks in the BM. The discovery cohort also included chimerism analysis in spleen, lymph nodes, liver, lung, thymus, colon and peritoneal cavity at 20 weeks post transplant. Secondary engraftment potential was evaluated by retransplanting 5 × 10^6^ total BM (tBM) cells or by single-cell transplantations of donor-derived HSCs (GFP^+^ or KuO^+^) from primary recipient mice.

### Bleeding and haematopoietic cell isolation

PB was withdrawn from the vena facialis and collected into EDTA-coated tubes. Blood cell counts were analysed using a Hemavet 950 FS (Drew Scientific) or ScilVet abc-Plus+ veterinary blood cell counting machine (Scil GmbH). For the comprehensive immunophenotypic characterization, haematopoietic cells were collected from the peritoneal cavity (PerCav) in 2 ml PBS, and haematopoietic organs and tissues were dissected, including bones, spleen, lymph nodes, thymus, lung and liver. BM was collected by isolating, cleaning and crushing the vertebral column, tibia, femur, limbs and sternum of killed mice in RPMI + 2% FCS. Cell suspensions were filtered through a 40-μm cell strainer, centrifuged and resuspended in ACK buffer for red blood cell lysis for 3 min at room temperature. After washing, 5 × 10^6^ BM cells were used for secondary transplantation, 3 × 10^7^ cells were kept for subsequent flow cytometric analysis and the remaining BM was used for scRNA-sequencing and stored in liquid nitrogen until further use. Lungs and liver were minced into small pieces. Lungs were further filtered initially through a 100-μm and, subsequently, through a 70-μm cell strainer. Liver, lymph nodes, spleen and thymus were filtered through a 40-μm cell strainer. Cell suspensions were spun down, resuspended in RPMI + 2% FCS and split for multiple flow cytometric analysis. Colons were turned inside out, cleaned and incubated in 25 ml extraction medium (RPMI 1640 + 2% FCS + 1 mM DTT + 0,5 mM EDTA) for 20 min at 37 °C to digest the intraepithelial layer. A total of 1 ml FCS was then added to block the digestion, and samples were filtered through a 40-μm cell strainer, centrifuged and resuspended in RPMI + 2% FCS for staining. If not stated otherwise, each step was performed on ice, RPMI or PBS supplemented with 2% FCS was used for washing and resuspending and centrifugation was done at 600*g*, 4 °C for 5 min. For the large-scale validation cohort, the same experimental protocol was followed for the isolation of BM haematopoietic cells.

### Flow cytometry analysis

#### Isolation of murine EPCR^hi^ LT-HSCs cells via FACS

BM cell suspension was subjected to depletion of mature blood cell lineages incubating with a mix of rat anti-mouse biotin-conjugated lineage markers (4.2 µg ml^−1^ CD5, 4.2 µg ml^−1^ CD8a, 2.4 µg ml^−1^ CD11b, 2.8 µg ml^−1^ B220, 2.4 µg ml^−1^ Gr-1, 2.6 µg ml^−1^ Ter-119) for 40 min at 4 °C. After incubation, cells were washed once with PBS + 2% FBS, span down at 350*g* at 4 °C for 5 min, resuspended in 800 µl PBS + 2% FBS and mixed with 800 µl Biotin Binder Dynabeads (Thermo Fisher), which were previously washed (two washes with PBS + 2% FBS). Beads were added at a concentration of 1 ml beads /1 × 10^8^ cells. Cells-beads mix was incubated for 45 min at 4 °C with constant rotation. Subsequently, lineage-positive cells were depleted using a magnetic particle concentrator (Dynal MPC-6, Invitrogen), and the resulting LSK-enriched fraction was washed once with PBS + 2% FBS and stained with the panel of antibodies indicated in Supplementary Tables 1 and 2 for 30 min at 4 °C. After the incubation, the stained cells were washed once with PBS + 2% FBS, resuspended in a final concentration of 2 ml PBS + 2% FBS and filtered through a 40-µm cell strainer FACS tube before the sort. All sorting experiments were performed using a BD FacsAria I or II flow cytometer (BD Bioscience) with a 100-μm nozzle and single-cell purity. Single EPCR^hi^ LT-HSCs (Supplementary Fig. 2) were sorted into round-bottom 96-well plates with 100 μl RPMI + 2% FBS with a cooling system. After the sort, 100 µl of supportive tBM at a concentration of 1.5 × 10^6^ cells ml^−1^ was added in each well on top of the sorted single HSC using a multichannel pipette, reaching a final volume of 200 µl per well.

#### Antibody-based staining of haematopoietic cells

PB, BM, spleen, lymph nodes, liver, lung, thymus, colon and peritoneal cavity cell suspensions were stained using monoclonal antibodies recognizing cell-specific surface proteins. Cells were incubated with an antibody mix prepared in PBS + 2% FBS. For organ-derived haematopoietic staining, cell suspensions had a concentration of 1 × 10^5^ cells µl^−1^ antibody mix. For white blood cell staining, 50 µl PB was incubated with 100 µl antibody mix. Blood platelet and erythrocyte staining involved 3 µl PB and 27 µl antibody mix. Cells were incubated for 30 min at 4 °C in the dark. All samples stained with antibodies against white blood cell epitopes were subjected to an erythrocyte lysis step using an ACK lysis buffer. Blood cells were incubated with ACK lysis buffer for 10 min, and remaining organ-derived haematopoietic cells were incubated with ACK lysis buffer for 2 min at room temperature. In case of the platelet and erythrocyte staining, this lysis step was not performed. After the lysis, cells were washed once with PBS + 2% FCS and resuspended in a final volume of PBS + 2% FCS. All samples were filtered before flow cytometry analysis.

#### Flow cytometry analysis

Cells were analysed by flow cytometry using a LSRFortessa or a LSRII cytometer (BD Biosciences), both equipped with 350-nm, 405-nm, 488-nm, 561-nm and 641-nm excitation lasers. Each antibody panel was manually compensated using OneComp eBeads (eBioscience) stained with single antibodies. Exemplar flow cytometry gating schemes can be found in Supplementary Fig. 3–13.

### Characterization of clonally derived haematopoietic systems by flow cytometry

#### Data preprocessing

Flow cytometry data were initially analysed in FlowJo (v10.6.1, BD). Each defined cell population was divided into their parental congenic origin GFP^+^CD45.1^+^ (donor), CD45.1/2^+^ (supportive BM) and CD45.2^+^ (recipient) and the cell count, or frequency of parent (FoP) was imported into R (v4.1). For count data, percent relative donor engraftment (DE) per cell population was calculated as follows: DE = #donor/(#donor + #supportive + #recipient). For frequencies, the FoP of donor-derived cells corresponded to DE. To account for technical noise, the lower bound detection limit was adjusted for by setting the cell populations’ DE with less than 20 detected events to NA and the DE of less than 0.1% to 0%. Further, cell populations that did not reach the detection threshold in at least ten analysed samples were excluded from downstream analysis. For PB reconstitution analysis of the discovery cohort, mice were excluded if they experienced graft failure post transplantation or did not reach an overall DE (that is, donor chimerism) of greater than 0.1% at any timepoint. For final timepoint analysis of the discovery cohort, mice were excluded if they did not reach sustained DE of at least 0.1% in any PBMC sample at week 20 post transplant.

#### Dimensionality reduction and clustering

For each clonally derived system, filtered DE levels of each organ-specific cell type were transformed into compositions. Before regression analysis, missing values were imputed by their mean. Dimensionality reduction was performed by PCA, and the top three dimensions were chosen for hierarchical clustering on principal components using the FactoMineR (v2.6) package. For comparison of the generated clusters with previously defined HSC subtypes, haematopoietic systems were classified as described in Dykstra et al.^6^ and visualized using ggtern (v3.4.2).

#### Relative repopulation capacity

The relative repopulation capacity of each haematopoietic system was calculated by dividing the overall PB chimerism levels (filtered DE levels of all blood cell types per system) from the secondary transplantation by its corresponding chimerism levels from the primary transplantation per week.

#### Model fitting

Haematopoietic reconstitution kinetics were modelled by fitting the filtered DE levels of blood cells per haematopoietic system for each available timepoint using the ‘single humped function’ that is described as: *x*(*t*) = 0, if *t* < *τ*; *x*(*t*) = *A* × (*t* − *τ*)/(1 + ((*t* − *τ*)/*θ*)^*n*^), if *t* ≥ *τ*, where *τ* is the delay, *A* the amplitude, *θ* the repression coefficient and *n* is the Hill coefficient. Parameter fitting was performed in Julia (v1.6) using the ModelFitter package (https://github.com/vkumpost/ModelFitter). Curve-specific characteristics (kinetic parameters) for each fitted curve were calculated as follows:

$${t}_{0}=\left\{\begin{array}{cc}\tau & \mathrm{if}\,{t}_{y\mathrm{Max}}\le {t}_{\mathrm{Max}}\\ 0 & \mathrm{otherwise}\end{array}\right.$$

$${y}_{\mathrm{Max}}=\left\{\begin{array}{cc}\frac{A\times \theta }{n}{\left(n-1\right)}^{(1-1/n)} & \mathrm{if}\,{t}_{y\mathrm{Max}}\le {t}_{\mathrm{Max}}\\ 0 & \mathrm{otherwise}\end{array}\right.$$

$$\mathrm{Slope}=\left\{\begin{array}{cc}\frac{x(t+{t}_{{n}})-x(t)}{{t}_{{n}}} & \mathrm{if}\,\tau < 20\\ 0 & \mathrm{otherwise}\end{array}\right.$$

$${t}_{y\mathrm{Max}}=\left\{\begin{array}{cc}{t}_{0}+\frac{\theta }{{\left(n-1\right)}^{(1/n)}} & \mathrm{if}\,{y}_{\mathrm{Max}} > 0\\ 0 & \mathrm{otherwise}\end{array}\right.$$

$${t}_{\mathrm{Growth}}=\left\{\begin{array}{cc}\frac{\theta }{{\left(n-1\right)}^{\left(1/n\right)}} & \mathrm{if}\,{y}_{\mathrm{Max}} > 0\\ 0 & \mathrm{otherwise}\end{array}\right.$$

$${t}_{\mathrm{HalfReg}}=\left\{\begin{array}{cc}{t}_{\mathrm{Half}}-\tau & \mathrm{if}\,{y}_{\mathrm{Max}} > 0\\ 0 & \mathrm{otherwise}\end{array}\right.$$

$${t}_{\mathrm{Decline}}=\left\{\begin{array}{cc}{t}_{\mathrm{Half}}-{t}_{y\mathrm{Max}} & \mathrm{if}\,{y}_{\mathrm{Max}} > 0\\ 0 & \mathrm{otherwise}\end{array}\right..$$

*t*_Half_ needed to be estimated using the Gauss–Newton method for nonlinear systems. This was done by newtonsys(Ffun = x(t) – 0.5*yMax, x0 = tyMax + x0) from pracma (v2.3.8). The AUC for each fitted curve was calculated using the auc function from flux (v.0.3). Fits were excluded if the RMSE was >0.08. All kinetic parameters except *y*_Max_, *t*_*y*__Max_ and AUC were set to NA if no decline was observed at the end of the study (*t*_Max_). Parameters *y*_Max_, *t*_*y*Max_ and AUC were set to its value at *t*_Max_. All parameters were set to NA, if no chimerism was observed (*t*_0_ = *t*_Max_).

#### Correlation analysis

Correlation analysis was performed using the rcorr() function from the Hmisc (v4.7-1) package. If not stated otherwise, Spearman rank correlation was used as a method. Polyclonal controls were excluded for these analyses. For visualization, either ComplexHeatmap (v2.10.0) or corrplot (v0.92) was used.

#### HSPC transition

For each clonally derived system, filtered DE levels of each HSPC were transformed into compositions. The compositions were ordered clockwise by their Pearson correlation distance to HSCs. The HSPC transition for each system was defined as the radian from HSC to median composition.

#### Hierarchical clustering

Haematopoietic systems were clustered using hierarchical clustering on parameters *t*_0_ and AUC with Euclidean distance, ward.D2 as algorithm and *k* = 4 clusters (stats::hclust(), (v4.1.0)). Entanglement with clusters from PCA analysis was visualized and calculated using dendextend (v1.15.2). A Kruskal–Wallis test was used to assess significant differences between the kinetic parameters and the three groups for each blood cell type.

### Characterization of clonally derived haematopoietic systems by scRNA-seq data

#### scRNA-seq and data preprocessing

For scRNA-seq, the Chromium Single Cell 3′ kit (v3.1) was used according to the manufacturer’s instructions. Libraries were sequenced on an Illumina HiSeq4000. FastQ files were processed and aligned using the Cell Ranger pipeline (v3.1) and the murine reference genome GRCm38 (mm10).

#### Quality control and batch integration

Each individual sample was loaded into a SeuratObject (v4.0.4) using the Seurat framework (v4.1.0) for downstream analysis. cKit+ and tBM cells were filtered separately. cKit+ cells were kept if they had 700–6,000 features, 1,400–45,000 counts and less then 10% mitochondrial reads. tBM cells were retained if they had 300–5,500 features, 1,000–40,000 counts and less than 8% mitochondrial reads. The data were log-normalized, and the top 3,000 variable features were scaled according to Seurat defaults. For data integration, LIGER was used via SeuratWrappers (v0.3.0) with default parameters, besides *k* = 50. Samples were treated as independent batches.

#### Dimensionality reduction and clustering

The 50 factors generated from the data integration via LIGER were used for further dimensionality reduction into two-dimensional space using uniform manifold approximation and projection (UMAP), as well as for Louvain clustering with a final resolution of 0.9. Final annotation was performed based on known marker genes for each population.

#### Differential abundance analysis

For differential abundance analysis, cell counts were transformed to compositions for each sample. Changes in abundance were assessed by calculating the log_2_-fold change difference between each clonally derived cell type fraction and the corresponding polyclonal control fraction that was summarized as mean.

#### Pseudotime analysis

Slingshot (v2.2.1) was used to calculate pseudotime trajectories for the progenitor compartment. The HSPC compartment was subset from the global dataset. The HSC cluster was chosen as the starting point and the distinct progenitors as endpoints. The UMAP was used as dimensionality input, on which the minimum spanning tree was calculated with default parameters. The curves were fitted using getCurves(extend = “n”, stretch =0).

#### Modelling chimerism dynamics in mature blood populations

To investigate the differences in chimerism dynamics between fast and slow clonal systems in mature blood populations, an ordinary differential equation model was constructed. The model consists of three hierarchically arranged stem cell populations, subsequent progenitor populations and a final mature cell compartment. The number of populations downstream of stem cells was set to ten to account for progressive maturation of progenitor/precursor cells. Production of blood cells from the upstream compartments along the haematopoietic hierarchy is allowed by differentiation reactions. Chimerism dynamics were described by the following ordinary differential equation system

$$\frac{{\rm{d}}{f}_{{\mathrm{HSC}}_{{\rm{U}}}}^{* }\left(t\right)}{{\rm{d}}t}=0$$

$$\frac{{\rm{d}}{f}_{{\mathrm{HSC}}_{{\rm{M}}}}^{* }(t)}{{\rm{d}}t}={\alpha }^{* }(\,{f}_{{\mathrm{HSC}}_{{\rm{U}}}}^{* }(t)-{f}_{{\mathrm{HSC}}_{{\rm{M}}}}^{* }(t))$$

$$\frac{{\rm{d}}{f}_{{\mathrm{HSC}}_{{\rm{D}}}}^{* }(t)}{{\rm{d}}t}={\alpha }^{* }(\,{f}_{{\mathrm{HSC}}_{{\rm{M}}}}^{* }(t)-{f}_{{\mathrm{HSC}}_{{\rm{D}}}}^{* }(t))$$

$$\frac{{\rm{d}}{f}_{{P}_{1}}^{* }(t)}{{\rm{d}}t}={\beta }^{* }(\,{f}_{{\mathrm{HSC}}_{{\rm{D}}}}^{* }(t)-{f}_{{P}_{1}}^{* }(t))$$

$$\frac{{\rm{d}}{f}_{{P}_{i}}^{* }(t)}{{\rm{d}}t}={\beta }^{* }(\,{f}_{{P}_{i-1}}^{* }(t)-{f}_{{P}_{i}}^{* }(t))$$

$$\frac{{\rm{d}}{f}_{{\rm{M}}}^{* }(t)}{{\rm{d}}t}={\beta }^{* }(\,{f}_{{P}_{n}}^{* }(t)-{f}_{{\rm{M}}}^{* }(t)),$$

where *f* generally denotes chimerism, with the subscript indicating the corresponding cell population. The model contains three stem subpopulations: upstream (HSC_U_), intermediate (HSC_M_) and downstream (HSC_D_) HSCs. HSC_U_ represent the tip, most primitive, self-sustaining stem cells, which go through differentiation stages HSC_M_ and HSC_D_ before contributing to mature lineages. Because tip stem cells are self-sustaining and receive no input, we model a constant chimerism in HSC_U_ (*f*_HSCU_)^40^. The constant value of chimerism in HSC_U_ (*f*_HSCU_), as well as the initial value of chimerism in HSC_D_ (*f*_HSCD_), were estimated from the experimental data; this was possible because these values determine the measured chimerism in mature populations. *P*_*i*_ denotes the *i*th progenitor population along the progressive maturation, with *i* = 1,…,*n*, and M denotes a mature cell compartment. The number of maturation steps (*n*) for each lineage is not precisely known and was set to *n* = 10. Indeed, with *n* = 10 fit quality was substantially better than for smaller *n*, for all mature lineages (as determined by the Bayesian information criterion), whereas no further improvement was seen for increasing *n* beyond 10 (Supplementary Fig. 1).

The model was separately fitted to average chimerism dynamics of seven haematopoietic lineages denoted by the asterisk: platelets, red blood cells, monocytes, granulocytes, B cells, CD4^+^ T cells and CD8^+^ T cells. For each lineage, average chimerism dynamics in slow and fast clusters were fitted simultaneously. The clusters were identified by hierarchical clustering of chimerism values in stem cells and mature lineages (Supplementary Fig. 1a). For each differentiation step, involving a progenitor and a product population pair, *α* and *β* represent the product of the respective differentiation rate and compartment size ratio of the progenitor and product populations, as previously derived in ref. ^40^. Initial chimerism values were estimated for upstream and downstream stem cell populations in slow and fast clusters and set to zero for other populations. Thus, the fitted parameters of the model include two dynamical rates and four initial conditions:

(1) *α* [1/day]: effective differentiation rate from upstream HSCs (HSC_U_) to downstream HSCs (HSC_D_)

(2) *β* [1/day]: effective differentiation rate from HSC_D_ to mature cells (M)

(3) *f*_HSCU,fast_: initial chimerism in the most upstream HSC population (HSC_U_) in the fast cluster,

(4) *f*_HSCD,fast_: initial chimerism in the most downstream HSC population (HSC_D_) in the fast cluster,

(5) *f*_HSCU,slow_: initial chimerism in the most upstream HSC population (HSC_U_) in the slow cluster,

(6) *f*_HSCD,slow_: initial chimerism in the most downstream HSC population (HSC_D_) in the slow cluster.

Bayesian inference package Turing.jl (v0.24.0) was used to estimate the above model parameters in Julia (v1.8.5).

#### Mathematical modelling of the coefficient of variation in linear and feedback compartment models

To address our observation that cell counts in mature blood populations display significantly lower coefficients of variation than LT-HSCs, we simulated two compartment models. Both models consist of three populations: stem cells (*S*), progenitors (*P*) and mature cells (*M*). Stem cells proliferate with rate *λ*_*S*_ and differentiate into progenitor cells with rate *δ*_*S*_. Progenitor cells, in turn, proliferate with rate *λ*_*P*_ and differentiate into mature cells with rate *δ*_*P*_. Mature cells undergo cell death with rate *δ*_*M*_. In the linear model, all proliferation and differentiation fluxes are proportional to the respective population sizes. In the feedback model, progenitor cell proliferation is governed by negative feedback and is implemented using a carrying capacity for the progenitor population, *P*_C_; this ensures stable regulation of mature cell numbers the dynamics of the linear model are described by the following ordinary differential equation system

$$\frac{{\rm{d}}S(t)}{{\rm{d}}t}={\lambda }_{{\rm{S}}}S(t)-{\delta }_{{\rm{S}}}S(t)$$

$$\frac{{\rm{d}}P(t)}{{\rm{d}}t}={\lambda }_{{\rm{P}}}P(t)-{\delta }_{{\rm{P}}}P(t)+{\delta }_{{\rm{S}}}S(t)$$

$$\frac{{\rm{d}}M(t)}{{\rm{d}}t}={\delta }_{{\rm{P}}}P(t)-{\delta }_{{\rm{M}}}M(t).$$

Similarly, the feedback model is described by the following nonlinear ordinary differential equation system.

$$\frac{{\rm{d}}S(t)}{{\rm{d}}t}={\lambda }_{{\rm{S}}}S(t)-{\delta }_{{\rm{S}}}S(t)$$

$$\frac{{\rm{d}}P(t)}{{\rm{d}}t}={\lambda }_{{\rm{P}}}P(t)\left(1-\frac{P(t)}{{P}_{{\rm{C}}}}\right)-{\delta }_{{\rm{P}}}P(t)+{\delta }_{{\rm{S}}}S(t)$$

$$\frac{{\rm{d}}M(t)}{{\rm{d}}t}={\delta }_{{\rm{P}}}P(t)-{\delta }_{{\rm{M}}}M(t).$$

Simulations for both models were initiated with hundred cells in the stem cell compartment (*S*_0_ = 100, *P*_0_ = 0, *M*_0_ = 0) and propagated up to 300 days. Proliferation and differentiation rates of the linear compartment model were set to the following values: *λ*_S_ = 0.1, *δ*_S_ = 0.1, *λ*_P_ = 2.0, *δ*_P_ = 2.02, *δ*_M_ = 0.1. For the feedback model the following rates were used: *λ*_S_ = 0.1, *δ*_S_ = 0.1, *λ*_P_ = 2.1, *δ*_P_ = 2.02, *P*_C_ = 10500, *δ*_M_ = 0.1. Coefficients of variation for individual compartments were computed from hundred independent simulations and normalized to the stem cell compartment. Simulations were performed with CoRC (v0.11.0, COPASI v4.34)^46,47^ in R (v3.6.1).

#### Lineage bias analysis from pooled barcoded transplantation experiments

scRNA-seq data from LARRY-barcoded transplantations were re-analysed from publicly available datasets (GSE299000 and GSE134242). These datasets consist of LARRY–EGFP-labelled CD45.2 HSCs isolated from 4–8-week-old donor mice and transplanted into lethally irradiated CD45.1 recipient mice, with approximately 1,000 barcoded HSCs transplanted per recipient. Processed count matrices were re-analysed to quantify clonal lineage biases. Cells lacking detectable barcodes, clones represented by fewer than two cells, and clones without at least one barcoded HSC were excluded from downstream analyses. For the remaining clones, a clone-by-cell-type pivot table was generated based on cell type annotations. To account for uneven sampling and barcode silencing across cell types, clonal contributions were normalized within each annotation before bias calculation. Lineage bias metrics were then computed as previously described^8^. For the assessment of megakaryocyte/myeloid (Mk/My) bias, megakaryocyte and myeloid clonal fractions were averaged and treated as a single combined compartment.

### Statistics and reproducibility

Statistical analysis and visualization in this study were performed using ggplot2 (v3.4.2) or FlowJo (v10.6.1). ComplexHeatmap (v2.10.0), corrplot (v0.92), dendextend (v1.15.2), DESeq2 (v1.30.0), FactoMineR (v2.6), flux (v0.3), ggplot2 (v3.4.2), ggtern (v3.4.2), Hmisc (v4.7-1), pracma (v2.3.8), Seurat (v4.1.0), SeuratWrappers (v0.3.0), slingshot (v2.2.1), CoRC (v0.11.0), COPASI (v4.34), Julia (v1.11.7), DifferentialEquations (v7.16.1), PairPlots (v3.0.3) and Turing (v0.40.4) packages were used for data analysis. If not specifically stated otherwise, significance was tested using paired samples Wilcoxon test. Normality was tested using the Shapiro–Wilk test. For multiple comparisons, *P* values were adjusted according to Benjamini and Hochberg. The sample size for single cell transplantation experiments was decided based on the rate of positive engraftment of single HSCs seen in prior publications (Yamamoto et al., 2013)^7^. The sample size for secondary single-cell transplantation experiments, *M*_2_, was determined from following estimations: *M*_2_ ≥ *M*/(*p*_sur_*pα*), where *M*, the number of recipient mice with high stem cell potency we aimed to obtain after transplantation, was set to 5 for statistical significance. A recipient mouse was considered highly potent if proportion of GFP^+^ cells exceeded 1% across all cell types. *p*_sur_, survival probability after transplantation, and *p*, the probability of obtaining a recipient with high stem cell potency upon single cell primary transplantation were obtained from previous data and equalled 66.7% and 10.7%, respectively. Parameter *α* represents the decline in repopulation potential of donor stem cells during ageing in the primary recipient (0 ≤ *α* ≤ 1) and was estimated by using previous data on platelet reconstitution in PB as proxy for LT-HSC potency. Under the assumption of a linear potency loss over time, the observed reduction in platelet reconstitution to 90% from week 16 to week 20 in primary transplantation was extrapolated to *α* = 90%^6^ ≈ 53% for the total observation period of 24 weeks in the primary recipient. The above yields *M*_2_ ≥ 141. Analogous sample size estimation was performed for secondary bulk transplantation. *M*_2B_ ≥ *M*/(*p*_sur_*pαn*), where *M*_2B_ is the number of secondary recipients, and *n* is the number of transplanted stem cells. *n* = 10 yields *M*_2B_ ≥ 14. In the discovery cohort, mice were excluded for PB analysis if they experienced graft failure post transplantation or if they did not reach an overall donor chimerism of >0.1% at any timepoint. For final timepoint analysis, mice were excluded if they did not reach sustained chimerism of at least 0.1% in any PB cell population at week 20 post transplant. To account for technical noise in flow cytometry analysis, the lower bound detection limit was adjusted by setting cell populations with less than 20 events recorded to NA and chimerism of less than 0.1% to 0. In the validation cohort, these exclusion criteria were not applied. In the *Rag2*^−/−^ cohort, the exclusion criteria of populations with less than 20 events recorded was also applied. All single cell transplantation experiments (including primary, secondary and *Rag2*^−/−^ transplantations) were repeated at least three times. Allocation of mice to groups was not formally randomized. No experiments were blinded.

### Reporting summary

Further information on research design is available in the Nature Portfolio Reporting Summary linked to this article.

## Online content

Any methods, additional references, Nature Portfolio reporting summaries, source data, extended data, supplementary information, acknowledgements, peer review information; details of author contributions and competing interests; and statements of data and code availability are available at https://doi.org/10.1038/s41556-026-01958-0.

## Supplementary information

Supplementary Information Supplementary Figs. 1–13.

Reporting Summary

Peer Review File

Supplementary Video 1 Animated schematic with text narration to illustrate how different kinetics of HSC contribution to mature blood cell production can, in concert with inter-clonal competition, result in apparent lineage-skewed output.

Supplementary Data 1 Statistical source data.

Supplementary Tables Supplementary Table 1 Information of antibodies used for the isolation of GFP^+^ EPCRhi LT-HSCs. Supplementary Table 2 Information of antibodies used for the isolation of KuO^+^ EPCRhi LT-HSCs.

## Source data

Source Data Fig. 1 Statistical source data.

Source Data Fig. 2 Statistical source data.

Source Data Fig. 3 Statistical source data.

Source Data Fig. 4 Statistical source data.

Source Data Fig. 5 Statistical source data.

Source Data Fig. 6 Statistical source data.

Source Data Extended Data Fig. 1 Statistical source data.

Source Data Extended Data Fig. 2 Statistical source data.

Source Data Extended Data Fig. 3 Statistical source data.

Source Data Extended Data Fig. 4 Statistical source data.

Source Data Extended Data Fig. 5 Statistical source data.

Source Data Extended Data Fig. 6 Statistical source data.

Source Data Extended Data Fig. 7 Statistical source data.

Source Data Extended Data Fig. 8 Statistical source data.

Source Data Extended Data Fig. 9 Statistical source data.

Source Data Extended Data Fig. 10 Statistical source data.

## Data Availability

scRNA-seq data that support the findings of this study have been deposited via Zenodo at https://doi.org/10.5281/zenodo.19486868 (ref. ^48^). Previously published LARRY barcoding data that were re-analysed in this study are available under accession codes GSE299000 and GSE134242. All other data supporting the findings are available from the corresponding author on reasonable request. Source data are provided with this paper.

## Code availability

The code that was generated in this study is via Zenodo at https://doi.org/10.5281/zenodo.19486868 (ref. ^48^).
