# Supplementary material for: A kinetics-based model of haematopoiesis reveals extrinsic regulation of skewed lineage output from stem cells
Source: Nat Cell Biol. 2026 May 25;28(6):1127–40. doi: 10.1038/s41556-026-01958-0 (PMC13278965; doi:10.1038/s41556-026-01958-0)
Supplement: Supplementary file 1 — Supplementary Figs. 1–13. [file 41556_2026_1958_MOESM1_ESM.pdf]

nature cell biology

Supplementary information

https://doi.org/10.1038/s41556-026-01958-0

A kinetics-based model of haematopoiesis reveals extrinsic regulation of skewed lineage output from stem cells

In the format provided by the authors and unedited

Supplementary Figures

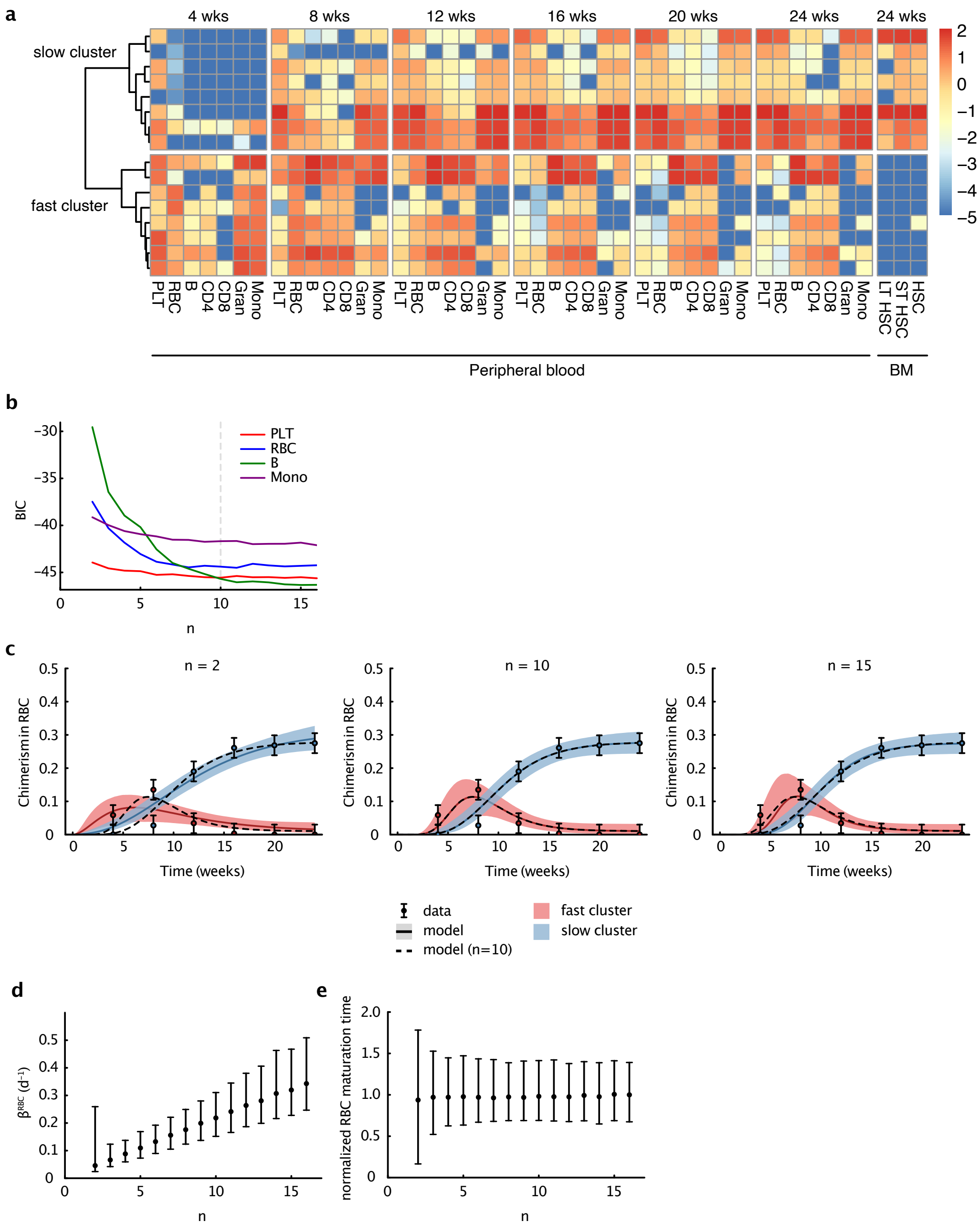

Supplementary Fig.1: Classification of clones into fast and slow engrafting and effects of maturation step number (n) on model behaviour. a) Hierarchical clustering of chimerism dynamics. Heatmap displaying the log_{10}-transformed percentage of chimerism in peripheral blood at different time points post-transplantation and in the bone marrow at the endpoint (24 weeks). Each row represents a single HSC transplantation. Slow (top) and fast (bottom) clusters were obtained by hierarchical clustering of time-resolved chimerism values. **b)** Models with various n were fitted to the experimental data and the Bayesian information criterion (BIC)

was calculated for each number of steps (n). Lines depict BIC for integer n values for platelets (PLT), red blood cells (RBC), B cells (B) and Monocytes (Mono). **c)** Exemplary model fits of chimerism dynamics for fast and slow clusters in RBCs with varied step numbers (n=2, n=10, n=15). Dashed lines in each plot depict the median dynamics with n=10, for comparison. **d)** Parameter estimates for model parameter β given models with different number of steps. Dots show median values. Error bars show 95% confidence intervals. **e)** Total normalized maturation time from HSCs to mature cells given models with different number of steps. Dots show median values. Error bars show 95% confidence intervals. Maturation time was calculated in arbitrary units as n/β and then normalized to the median value at maximum n.

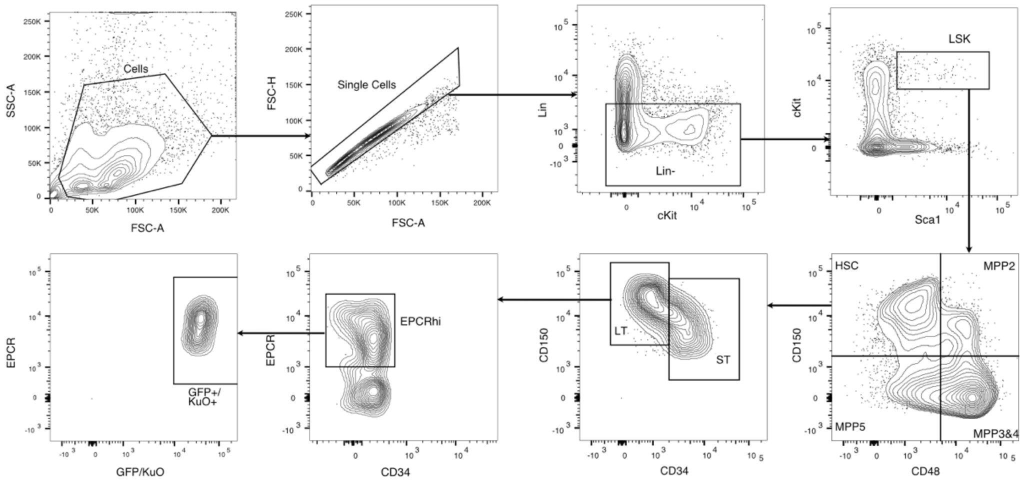

Supplementary Fig.2: Gating strategy to sort donor EPCRhi LT-HSCs for transplantations studies.

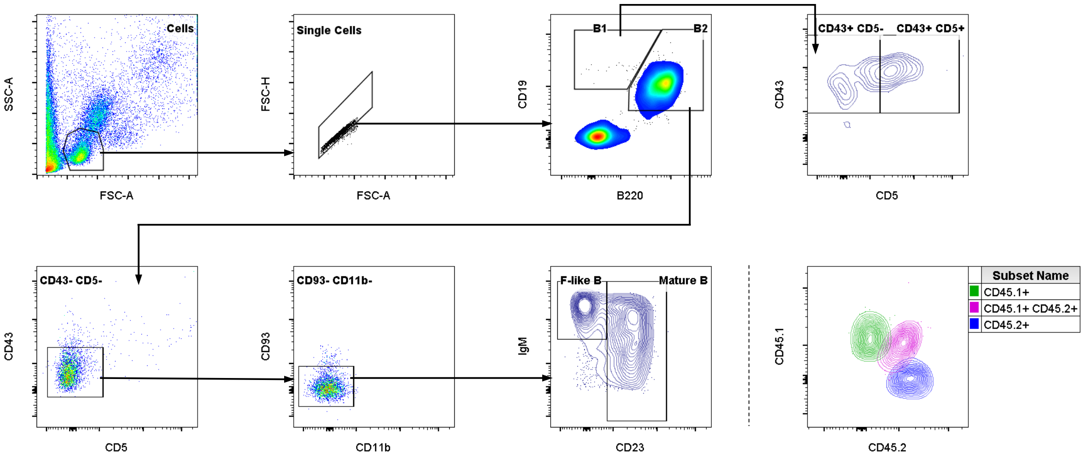

Supplementary Fig.3: Peripheral blood WBC gating strategy 1.

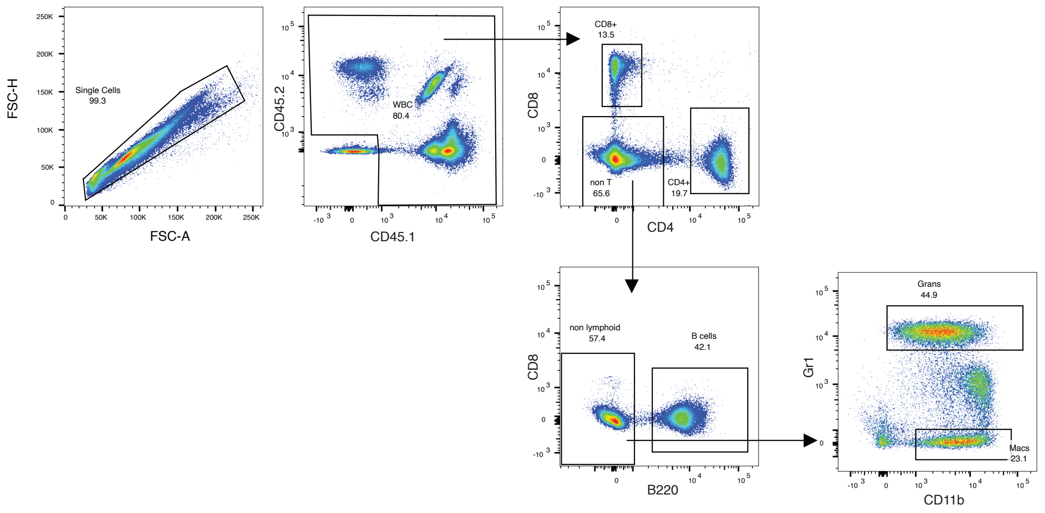

Supplementary Fig.4: Peripheral blood WBC gating strategy 2.

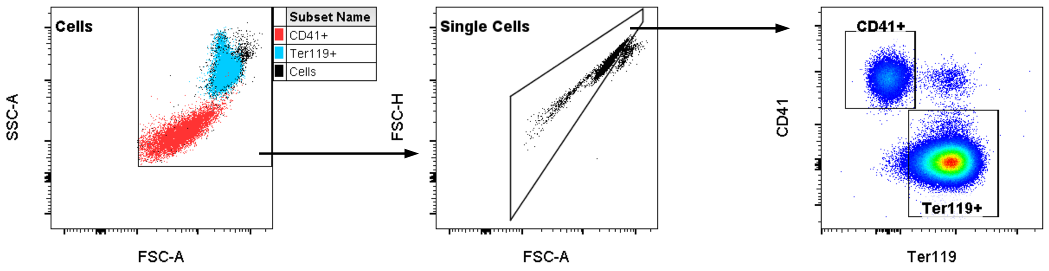

Supplementary Fig.5: Peripheral blood platelet and erythrocyte gating strategy.

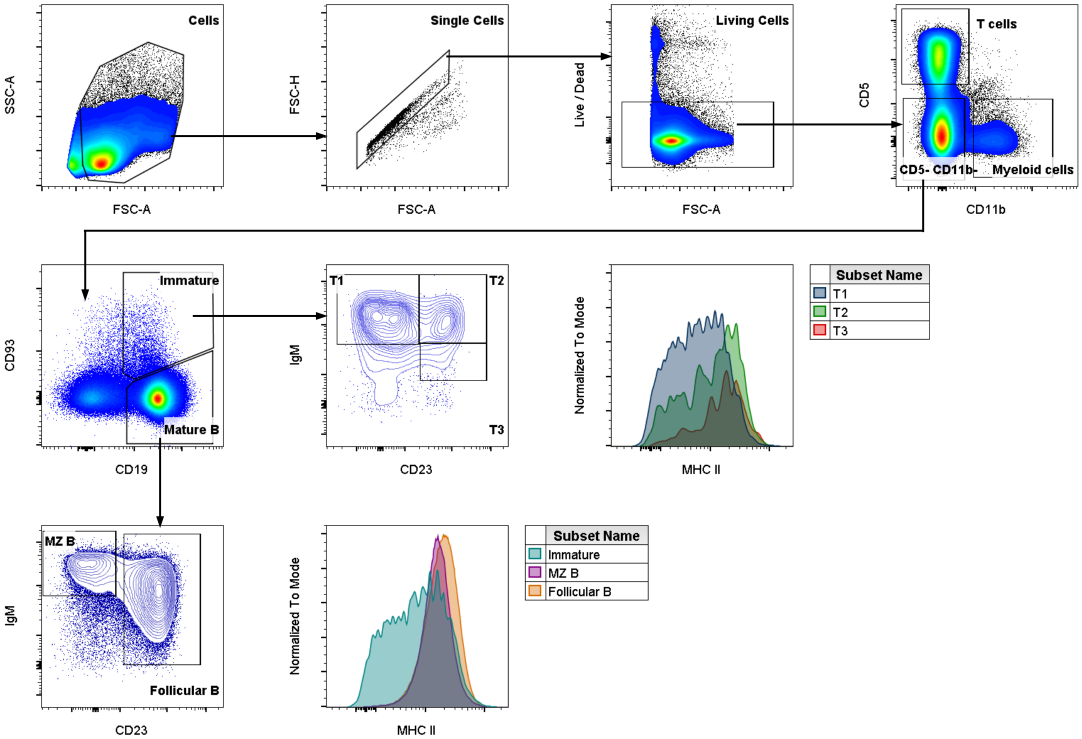

Supplementary Fig.6: Spleen gating strategy.

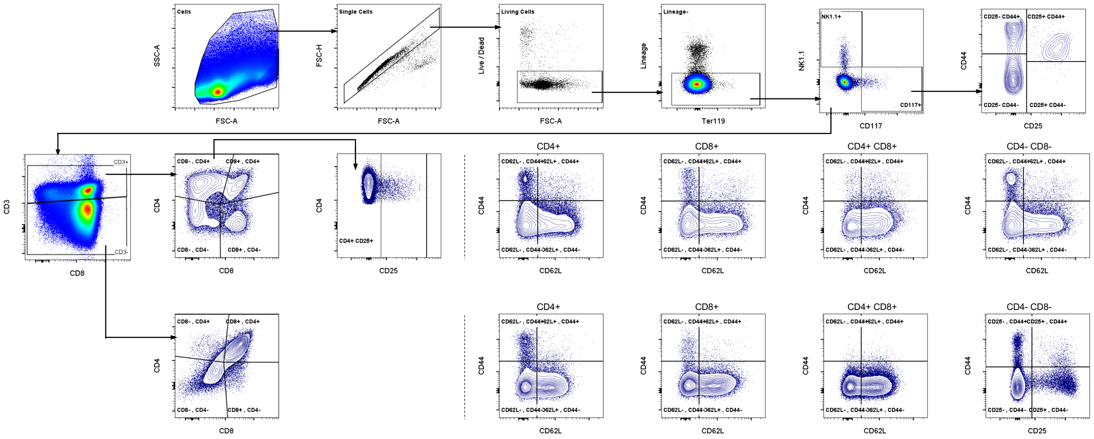

Supplementary Fig.7: Thymus gating strategy.

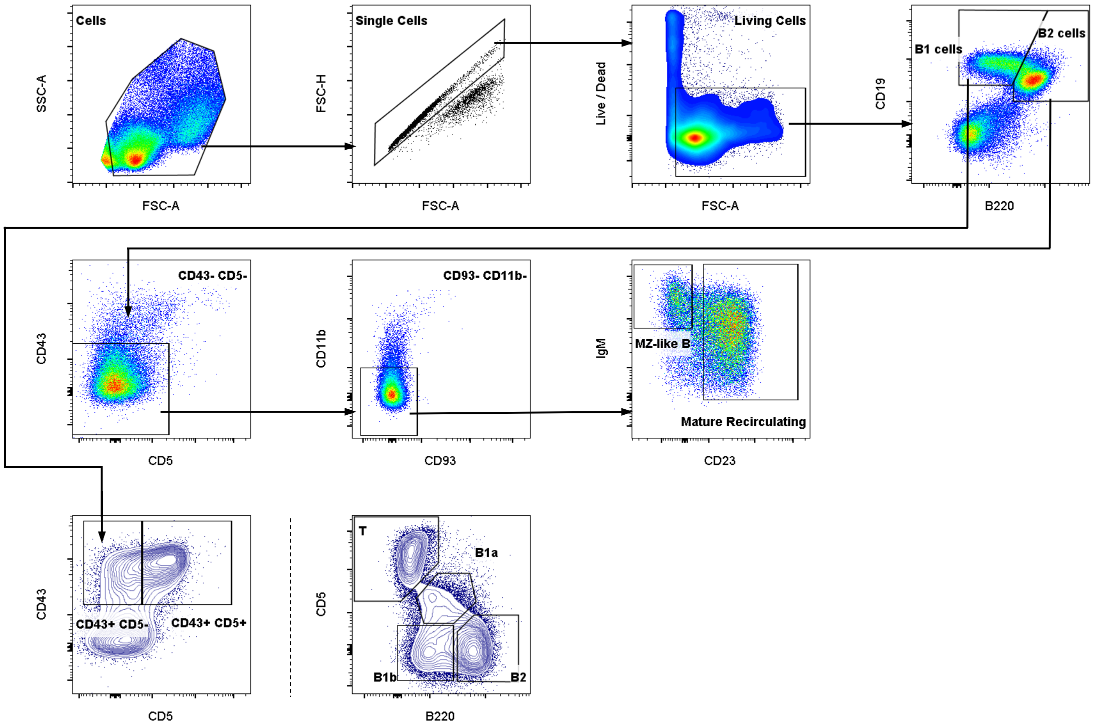

Supplementary Fig.8: Peritoneal cavity gating strategy.

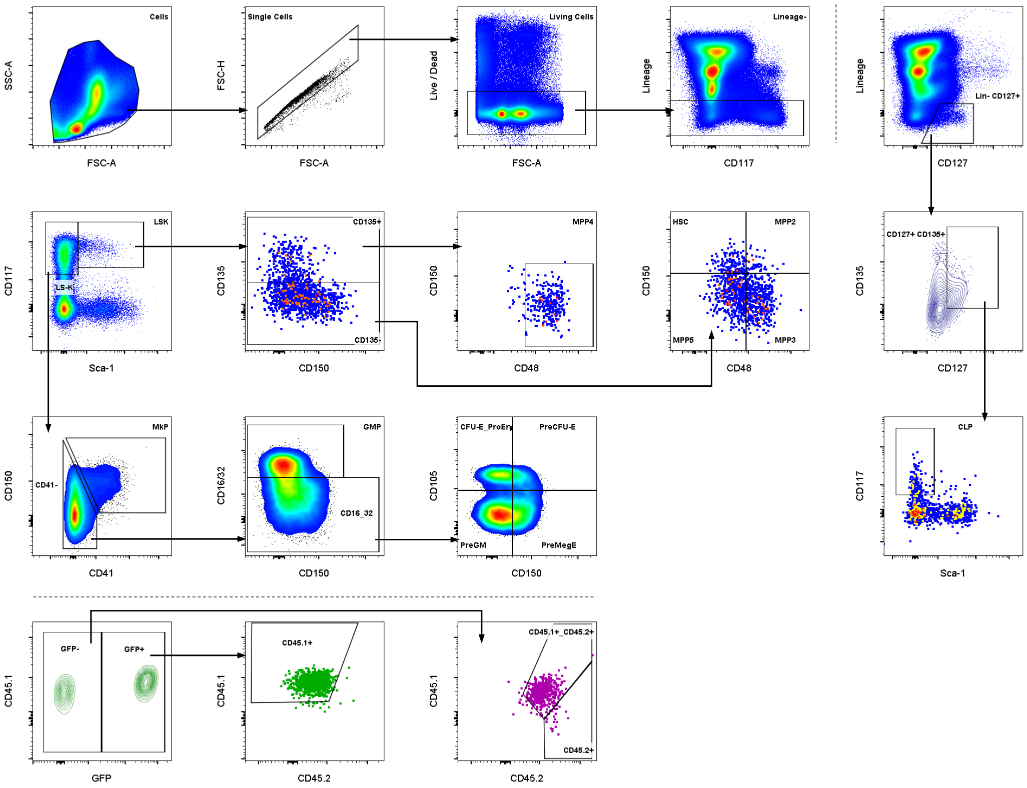

Supplementary Fig.9: Bone marrow gating strategy 1.

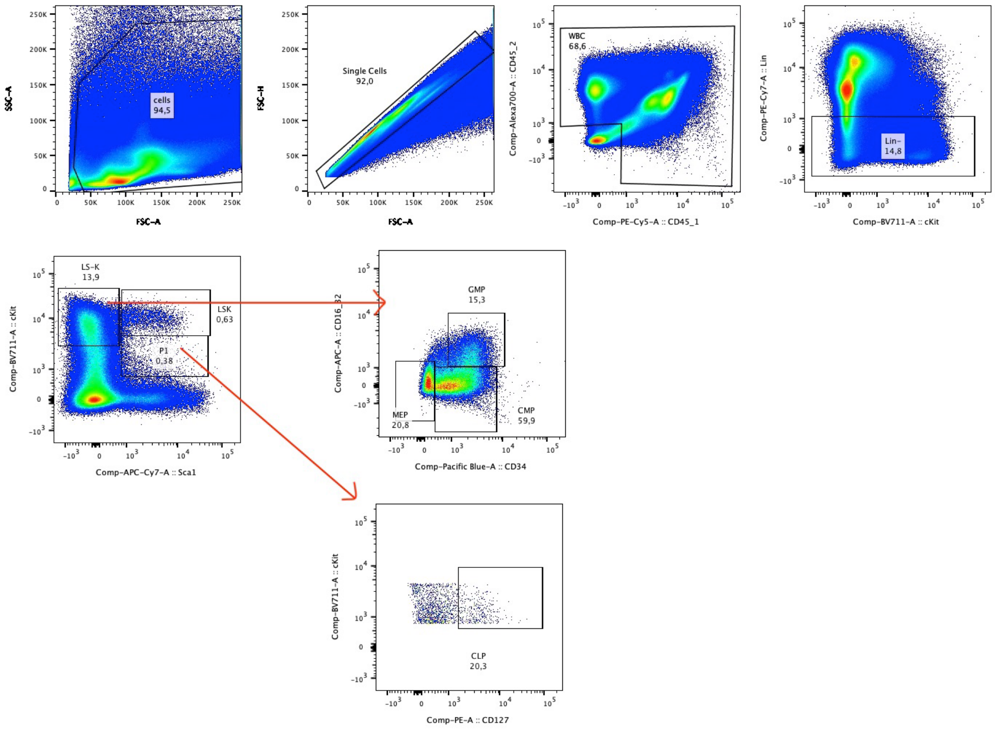

Supplementary Fig.10: Bone marrow gating strategy 2.

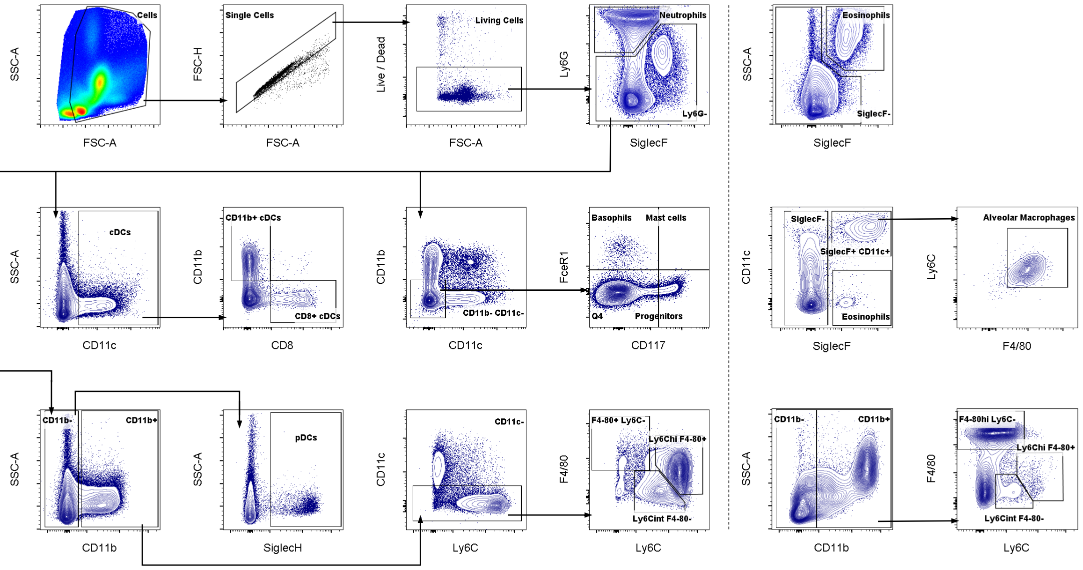

Supplementary Fig.11: Bone marrow gating strategy 3.

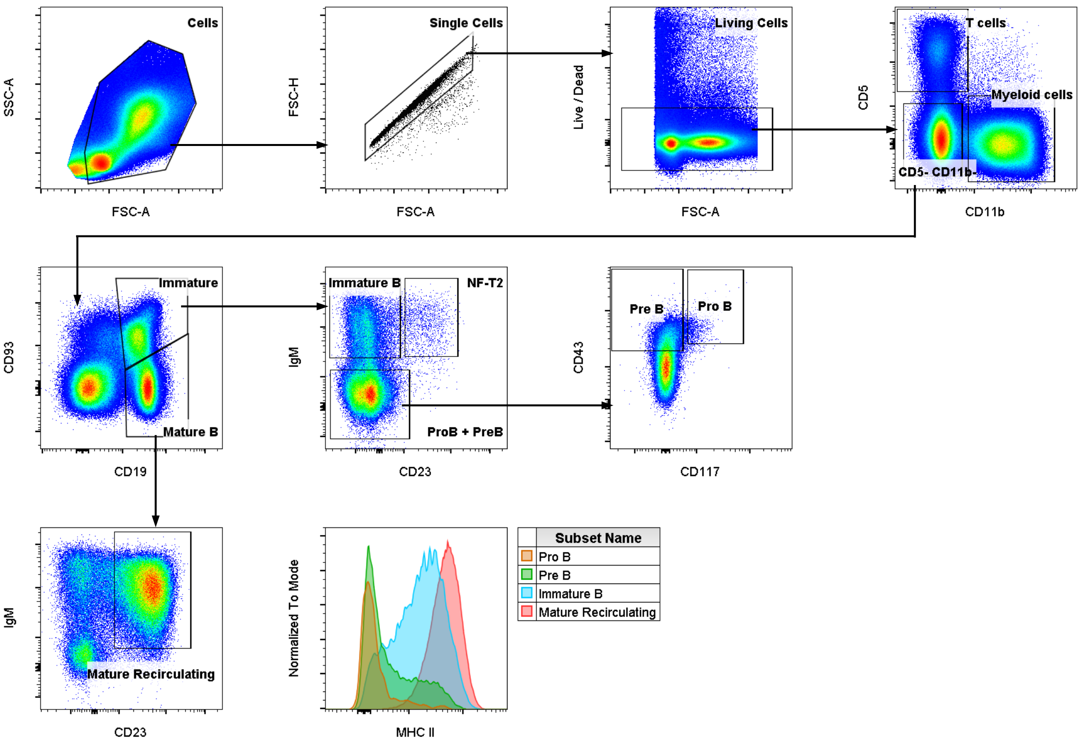

Supplementary Fig.12: Bone marrow gating strategy 4.

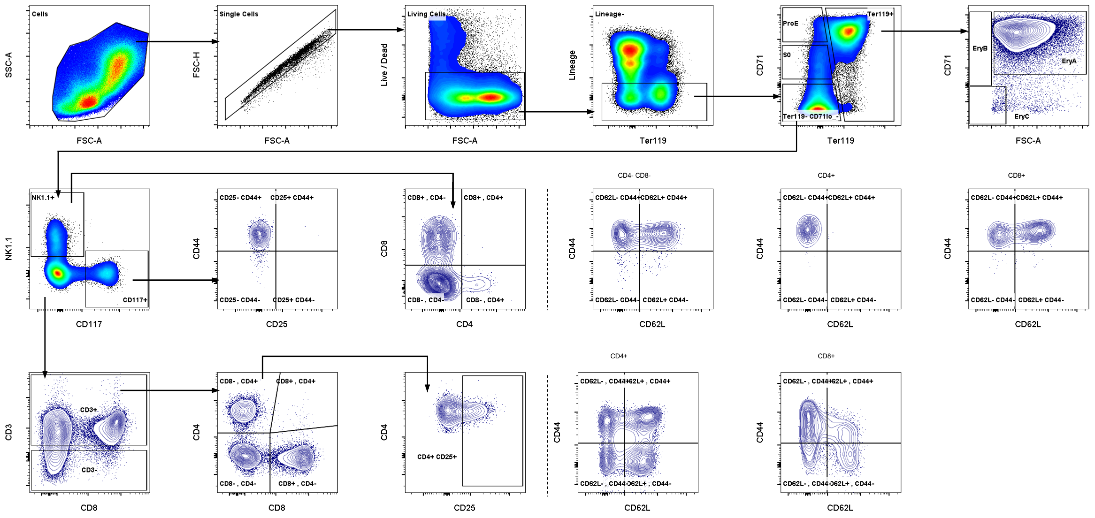

Supplementary Fig.13: Bone marrow gating strategy 5.
