# Supplementary material for: A kinetics-based model of haematopoiesis reveals extrinsic regulation of skewed lineage output from stem cells
Source: Nat Cell Biol. 2026 May 25;28(6):1127–40. doi: 10.1038/s41556-026-01958-0 (PMC13278965; doi:10.1038/s41556-026-01958-0)
Supplement: Supplementary file 2 — Reporting Summary [file 41556_2026_1958_MOESM2_ESM.pdf]

nature portfolio

Corresponding author(s): Michael Milsom, Simon Haas, Daniel Hübschmann & Thomas Höfer

Last updated by author(s): Apr 9, 2026

Reporting Summary

Nature Portfolio wishes to improve the reproducibility of the work that we publish. This form provides structure for consistency and transparency in reporting. For further information on Nature Portfolio policies, see our Editorial Policies and the Editorial Policy Checklist.

Statistics

For all statistical analyses, confirm that the following items are present in the figure legend, table legend, main text, or Methods section.

n/a	Confirmed	
☐	☒	The exact sample size (*n*) for each experimental group/condition, given as a discrete number and unit of measurement
☐	☒	A statement on whether measurements were taken from distinct samples or whether the same sample was measured repeatedly
☐	☒	The statistical test(s) used AND whether they are one- or two-sided<br>*Only common tests should be described solely by name; describe more complex techniques in the Methods section.*
☐	☒	A description of all covariates tested
☐	☒	A description of any assumptions or corrections, such as tests of normality and adjustment for multiple comparisons
☐	☒	A full description of the statistical parameters including central tendency (e.g. means) or other basic estimates (e.g. regression coefficient) AND variation (e.g. standard deviation) or associated estimates of uncertainty (e.g. confidence intervals)
☐	☒	For null hypothesis testing, the test statistic (e.g. *F*, *t*, *r*) with confidence intervals, effect sizes, degrees of freedom and *P* value noted<br>*Give P values as exact values whenever suitable.*
☐	☒	For Bayesian analysis, information on the choice of priors and Markov chain Monte Carlo settings
☐	☒	For hierarchical and complex designs, identification of the appropriate level for tests and full reporting of outcomes
☐	☒	Estimates of effect sizes (e.g. Cohen's *d*, Pearson's *r*), indicating how they were calculated

Our web collection on statistics for biologists contains articles on many of the points above.

Software and code

Policy information about availability of computer code

Data collection	Flow cytometry data were collected using a FACSDiva software (BD). Flow cytometry analysis was performed using a LSRFortessa or a LSRII cytometer (BD Biosciences). Sorting experiments were performed using BD FacsAria I or II cytometers (BD Biosciences). Blood cell counts were measured using a Hemavet 950 FS (Drew Scientific) or ScilVet abs-Plus+ veterinary blood cell counting machine (Scil GmbH).
Data analysis	Flow cytometry data were analyzed in FlowJo (v10.6.1, BD). Data processing was performed in Microsoft Excel for mac version 16.16.27. All graphs were generated and statistically analyzed using R (v4.1) and ggplot2 (v3.4.2). ComplexHeatmap (v2.10.0), corrplot (v0.92), dendextend (v1.15.2), DESeq2 (v1.30.0), FactoMineR (v2.6), flux (v0.3), ggplot2 (v3.4.2), ggtern (v3.4.2), Hmisc (v4.7-1), pracma (v2.3.8), Seurat (v4.1.0), SeuratWrappers (v0.3.0), slingshot (v2.2.1), CoRC (v0.11.0), COPASI (v4.34), Julia (v1.11.7), DifferentialEquations (v7.16.1), PairPlots (v3.0.3), Turing (v0.40.4) packages were used for data analysis. For scRNA-seq analysis of mouse bone marrow hematopoietic cells, libraries were prepared using the Chromium Single Cell 3´kit (v3.1) according to manufacturer´s instructions. Libraries were sequenced on an Illumina HiSeq4000. FastQ files were processed and aligned using the Cell Ranger pipeline (v3.1) and the murine reference genome GRCm38 (mm10). Hematopoietic reconstitution kinetics were modeled as described in detail in the methods section.

For manuscripts utilizing custom algorithms or software that are central to the research but not yet described in published literature, software must be made available to editors and reviewers. We strongly encourage code deposition in a community repository (e.g. GitHub). See the Nature Portfolio guidelines for submitting code & software for further information.

Data

Policy information about availability of data

All manuscripts must include a data availability statement. This statement should provide the following information, where applicable:

- Accession codes, unique identifiers, or web links for publicly available datasets
- A description of any restrictions on data availability
- For clinical datasets or third party data, please ensure that the statement adheres to our policy

All scripts used in the manuscript are available in Zenodo with the DOI https://doi.org/10.5281/zenodo.19486868. The processed single-cell RNA sequencing data from Fig.3 and Extended Data Fig.7 have been deposited in Zenodo with the DOI https://doi.org/10.5281/zenodo.19486868. Single-cell RNA-seq data from LARRY-barcoded transplantations shown in Fig. 5f-g were re-analyzed from publicly available datasets (GSE299000 and GSE134242).

Research involving human participants, their data, or biological material

Policy information about studies with human participants or human data. See also policy information about sex, gender (identity/presentation), and sexual orientation and race, ethnicity and racism.

Reporting on sex and gender	*Use the terms sex (biological attribute) and gender (shaped by social and cultural circumstances) carefully in order to avoid confusing both terms. Indicate if findings apply to only one sex or gender; describe whether sex and gender were considered in study design; whether sex and/or gender was determined based on self-reporting or assigned and methods used. Provide in the source data disaggregated sex and gender data, where this information has been collected, and if consent has been obtained for sharing of individual-level data; provide overall numbers in this Reporting Summary. Please state if this information has not been collected. Report sex- and gender-based analyses where performed, justify reasons for lack of sex- and gender-based analysis.*
Reporting on race, ethnicity, or other socially relevant groupings	*Please specify the socially constructed or socially relevant categorization variable(s) used in your manuscript and explain why they were used. Please note that such variables should not be used as proxies for other socially constructed/relevant variables (for example, race or ethnicity should not be used as a proxy for socioeconomic status). Provide clear definitions of the relevant terms used, how they were provided (by the participants/respondents, the researchers, or third parties), and the method(s) used to classify people into the different categories (e.g. self-report, census or administrative data, social media data, etc.) Please provide details about how you controlled for confounding variables in your analyses.*
Population characteristics	*Describe the covariate-relevant population characteristics of the human research participants (e.g. age, genotypic information, past and current diagnosis and treatment categories). If you filled out the behavioural & social sciences study design questions and have nothing to add here, write "See above."*
Recruitment	*Describe how participants were recruited. Outline any potential self-selection bias or other biases that may be present and how these are likely to impact results.*
Ethics oversight	*Identify the organization(s) that approved the study protocol.*

Note that full information on the approval of the study protocol must also be provided in the manuscript.

Field-specific reporting

Please select the one below that is the best fit for your research. If you are not sure, read the appropriate sections before making your selection.

☒ Life sciences ☐ Behavioural & social sciences ☐ Ecological, evolutionary & environmental sciences

For a reference copy of the document with all sections, see nature.com/documents/nr-reporting-summary-flat.pdf

Life sciences study design

All studies must disclose on these points even when the disclosure is negative.

Sample size	The sample size for single cell transplantation experiments was decided based on the rate of positive engraftment of single HSCs seen in prior publications (Yamamoto et al., 2013). The sample size for secondary single cell transplantation experiments, M2, was determined from following estimations: M2≥ M/(psurpα), where M, the number of recipient mice with high stem cell potency we aimed to obtain after transplantation was set to 5 for statistical significance. A recipient mouse was considered highly potent if proportion of GFP+ cells exceeded 1% across all cell types. psur, survival probability after transplantation, and p, the probability of obtaining a recipient with high stem cell potency upon single cell primary transplantation were obtained from previous data and equaled 66.7% and 10.7%, respectively. Parameter α represents the decline in repopulation potential of donor stem cells during aging in the primary recipient (0 ≤ α ≤ 1) and was estimated by using previous data on platelet reconstitution in peripheral blood as proxy for LT-HSC potency. Under the assumption of a linear potency loss over time, the observed reduction in platelet reconstitution to 90% from week 16 to week 20 in primary transplantation was extrapolated to α=90%^6≈53% for the total observation period of 24 weeks in the primary recipient. The above yields M2 ≥ 141. Analogous sample size estimation was performed for secondary bulk transplantation. M2B ≥ M/(psurpαn), where M2B is the number of secondary recipients and n is the number of transplanted stem cells. n=10 yields M2B ≥ 14.

Data exclusions	In the discovery cohort, mice were excluded for peripheral blood analysis if they experienced graft failure post-transplantation, or if they did not reach an overall donor chimerism of >0.1% at any timepoint. For final timepoint analysis, mice were excluded if they did not reach sustained chimerism of at least 0.1% in any peripheral blood cell population at week 20 post-transplant. To account for technical noise in flow cytometric analysis, the lower bound detection limit was adjusted by setting cell populations with less than 20 events recorded to NA, and chimerism of less than 0.1% to 0. In the validation cohort, these exclusion criteria were not applied. In the Rag2-/- cohort, the exclusion criteria of populations with less than 20 events recorded was also applied.
Replication	All single cell transplantation experiments (including primary, secondary and Rag2-/- transplantations) were repeated at least 3 times.
Randomization	Allocation of mice to groups was not formally randomized.
Blinding	No experiments were blinded.

Reporting for specific materials, systems and methods

We require information from authors about some types of materials, experimental systems and methods used in many studies. Here, indicate whether each material, system or method listed is relevant to your study. If you are not sure if a list item applies to your research, read the appropriate section before selecting a response.

Materials & experimental systems

n/a	Involved in the study
☐	☒ Antibodies
☒	☐ Eukaryotic cell lines
☒	☐ Palaeontology and archaeology
☐	☒ Animals and other organisms
☒	☐ Clinical data
☒	☐ Dual use research of concern
☒	☐ Plants

Methods

n/a	Involved in the study
☒	☐ ChIP-seq
☐	☒ Flow cytometry
☒	☐ MRI-based neuroimaging

Antibodies

Antibodies used

FACS sort of mouse EPCRhi LT-HSCs:
anti-CD4 AF700, clone GK1.5, 1:1000 (eBioscience, 56-0041-82, RRID:AB_493999) or PE-Cy7, GK1.5, 1:1000 (eBioscience, 25-0041-82, RRID:AB_469576)
anti-CD8 AF700, clone 53-6.7, 1:2000 (eBioscience, 56-0081-82, RRID:AB_494005) or PE-Cy7, clone 53-6.7, 1:1000 (eBioscience, 25-0081-82, RRID:AB_469584)
anti-B220 AF700, clone RA3-6B2, 1:200 (eBioscience, 56-0452-82, RRID:AB_891458) or PE-Cy7, clone RA3-6B2, 1:500 (eBioscience, 25-0452-82, RRID:AB_469627)
anti-CD11b AF700, clone M1/70, 1:500 (eBioscience, 56-0112-82, RRID:AB_657585) or PE-Cy7, clone M1/70, 1:1000 (eBioscience, 25-0112-82, RRID:AB_469588)
anti-Gr1 AF700, clone RB6-BC5, 1:2000 (eBioscience, 56-5931-82, RRID:AB_494007) or PE-Cy7, clone RB6-BC5, 1:2000 (eBioscience, 25-5931-82, RRID:AB_469663)
anti-Ter119 AF700, clone TER-119, 1:100 (Biolegend, 116220, RRID:AB_528963) or PE-Cy7, clone TER-119, 1:1000 (eBioscience, 25-5921-82, RRID:AB_469661)
anti-CD117 (cKit) BV711, clone 2B8, 1:2000 (Biolegend, 105835, RRID:AB_2565956) or APC, clone 2B8, 1:3000 (eBioscience, 17-1171-82, RRID:AB_469430)
anti-Sca1 (Ly-6A/E) APC-Cy7, clone D7, 1:1000 (BD Biosciences, 560654, RRID:AB_1727552)
anti-CD150 PE-Cy5, clone TC15-12F12-2, 1:1000 (Biolegend, 115912, RRID:AB_493598) or APC, clone TC15-12F12-2, 1:1000 (Biolegend, 115910, RRID:AB_493460)
anti-CD48 PE-Cy7, clone HM48-1, 1:1000 (Biolegend, 103424, RRID:AB_2075049) or BUV395, clone HM48-1, 1:300 (BD, 740236, RRID:AB_2739984)
anti-CD34 eFluor450, clone RAM34, 1:30 (eBioscience, 48-0341-82, RRID:AB_2043837)
anti-EPCR PE, clone eBio1560, 1:300 (eBioscience, 12-2012-82, RRID:AB_914317) or PerCP-eF710, clone eBio1560, 1:300 (eBioscience, 46-2012-80, RRID:AB_10718383)

Mature white blood cell analysis (discovery cohort):
Anti-CD117 (cKit) BV711, clone 2B8, 1:2000 (Biolegend, 105835, RRID:AB_2565956)
Anti-CD45.1 AF700, clone A20, 1:200 (Biolegend, 110724, RRID:AB_493733)
Anti-CD45.2 PerCP-Cy5.5, clone 104, 1:200 (Biolegend, 109828, RRID:AB_893350)
Anti-CD11b APCeF780, clone M1/70, 1:500 (eBioscience, 47-0112-82, RRID:AB_1603193)
Anti-CD19 BUV395, clone 1D3, 1:400 (BD, 563557, RRID:AB_2722495)
Anti-CD23 BV510, clone B2B4, 1:400 (Biolegend, 101623, RRID:AB_2563705)
Anti-CD43 PE-Cy7, clone S11, 1:800 (Biolegend, 143210, RRID:AB_2564349)
Anti-B220 eF450, clone RA3-6B2, 1:300 (eBioscience, 48-0452-82, RRID:AB_1548761)
Anti-CD5 PE-Cy5, clone 53-7.2, 1:500 (Biolegend, 100622, RRID:AB_2562773)
Anti-CD93 APC, clone AA4.1, 1:800 (eBioscience, 17-5892-82, RRID:AB_469466)
Anti-IgM PE, clone RMM-1, 1:250 (Biolegend, 406508, RRID:AB_315058)
Anti-MHCII(I-A/I-E) BV785, clone M5/114.15.2, 1:1200 (Biolegend, 107645, RRID:AB_2565977)

Myeloid cell analysis (discovery cohort):
Anti-CD117 (cKit) BV711, clone 2B8, 1:2000 (Biolegend, 105835, RRID:AB_2565956)
Anti-CD45.1 AF700, clone A20, 1:200 (Biolegend, 110724, RRID:AB_493733)
Anti-CD45.2 PerCP-Cy5.5, clone 104, 1:200 (Biolegend, 109828, RRID:AB_893350)
Anti-CD11b APC, clone M1/70, 1:2000 (eBioscience, 17-0112-82, RRID:AB_469343)
Anti-CD11c PE-Cy7, clone N418, 1:300 (Biolegend, 117318, RRID:AB_493568)
Anti-F4/80 PacificBlue, clone BM8, 1:100 (Invitrogen, MF48028, RRID:AB_10373419)
Anti-FceR1 APC-Cy7, clone MAR-1, 1:200 (Biolegend, 134326, RRID:AB_2572064)
Anti-Ly6C BV510, clone HK1.4, 1:300 (Biolegend, 128033, RRID:AB_2562351)
Anti-Ly6G biotin, clone 1A8, 1:300 (Biolegend, 127603, RRID:AB_1186105)
Anti-SinglecF BV786, clone E50-2240, 1:400 (BD Biosciences, 740956, RRID:AB_2740581)
Anti-SinglecH PE, clone eBio440c, 1:500 (eBioscience, 12-0333-82, RRID:AB_10597139)
Anti-Streptavidin PE-Cy5, 1:30 (eBioscience, 15-4317-82, RRID:AB_10116415)
Anti-CD8 BUV395, clone 53-6.7, 1:500 (BD Biosciences, 563786, RRID:AB_2732919)

Lymphoid cell analysis (discovery cohort):
Anti-CD117 (cKit) BV711, clone 2B8, 1:2000 (Biolegend, 105835, RRID:AB_2565956)
Anti-CD45.1 AF700, clone A20, 1:200 (Biolegend, 110724, RRID:AB_493733)
Anti-CD45.2 PerCP-Cy5.5, clone 104, 1:200 (Biolegend, 109828, RRID:AB_893350)
Anti-CD11b PE-Cy5, clone M1/70, 1:3000 (eBioscience, 15-0112-82, RRID:AB_468714)
Anti-B220 PE-Cy5, clone RA3-6B2, 1:1000 (eBioscience, 15-0452-82, RRID:AB_468755)
Anti-Gr1 PE-Cy5, clone RB6-8C5, 1:3000 (eBioscience, 15-5931-82, RRID:AB_468813)
Anti-CD8 BUV395, clone 53-6.7, 1:500 (BD Biosciences, 563786, RRID:AB_2732919)
Anti-CD25 APC, clone 3C7, 1:800 (Biolegend, 101910, RRID:AB_2280288)
Anti-CD3 BV510, clone 17A2, 1:100 (BD Biosciences, 740147, RRID:AB_2739902)
Anti-CD4 BUV805, clone GK1.5, 1:500 (BD Biosciences, 564922, RRID:AB_2739008)
Anti-CD44 eF450, clone IM7, 1:400 (eBioscience, 48-0441-82, RRID:AB_1272246)
Anti-CD62L PE-Cy7, clone MEL-14, 1:600 (Biolegend, 104418, RRID:AB_313103)
Anti-CD71 PE, clone C2, 1:200 (BD Biosciences, 553267, RRID:AB_394744)
Anti-NK1.1 BV785, clone PK136, 1:800 (Biolegend, 108749, RRID:AB_2564304)
Anti-Ter119 APCeF780, clone TER-119, 1:400 (eBioscience, 47-5921-82, RRID:AB_1548786)

Mature white blood cell analysis (validation cohort):
anti-CD4 PE-Cy5, clone GK1.5, 1:2000 (eBioscience, 15-0041-82, RRID:AB_468695) or BUV805, clone GK1.5, 1:500 (BD Biosciences, 564922, RRID:AB_2739008)
anti-CD8 PE-Cy7, clone 53-6.7, 1:1000 (eBioscience, 25-0081-82, RRID:AB_469584) or BUV395, clone 53-6.7, 1:500 (BD Biosciences, 563786, RRID:AB_2732919)
anti-B220 APC-Cy7, clone RA3-6B2, 1:300 (eBioscience, 47-0452-82, RRID:AB_1518810)
anti-CD11b APC, clone M1/70, 1:2000 (eBioscience, 17-0112-82, RRID:AB_469343)
anti-Gr1 PE, clone RB6-BC5, 1:3000 (eBioscience, 12-5931-82, RRID:AB_466045) or PE-Cy7, clone RB6-BC5, 1:1000 (eBioscience, 25-5931-82, RRID:AB_469663)
anti-CD45.1 PacificBlue, clone A20, 1:500 (Biolegend, 110722, RRID:AB_492866)
anti-CD45.2 AL700, clone 104, 1:100 (eBioscience, 56-0454-82, RRID:AB_657752)

Platelet and erythrocyte analysis
Anti-Ter119 APC, clone TER-119, 1:300 (eBioscience, 17-5921-82, RRID:AB_469473)
Anti-CD41 PE-Cy7, clone MWReg30, 1:200 (eBioscience, 25-0411-82, RRID:AB_1234970) or PE, clone MWReg30, 1:200 (eBioscience, 12-0411-82, RRID:AB_763485)

Bone marrow stem and progenitor cell analysis:
Anti-CD4 APC, clone GK1.5, 1:2000 (eBioscience, 17-0041-82, RRID:AB_469320) or PE-Cy7, GK1.5, 1:1000 (eBioscience, 25-0041-82, RRID:AB_469576)
Anti-CD8 APC, clone 53-6.7, 1:3000 (eBioscience, 17-0081-82, RRID:AB_469335) or PE-Cy7, clone 53-6.7, 1:1000 (eBioscience, 25-0081-82, RRID:AB_469584)
anti-B220 APC, clone RA3-6B2, 1:500 (eBioscience, 17-0452-82, RRID:AB_469395) or PE-Cy7, clone RA3-6B2, 1:500 (eBioscience, 25-0452-82, RRID:AB_469627)
anti-CD11b APC, clone M1/70, 1:2000 (eBioscience, 17-0112-82, RRID:AB_469343) or PE-Cy7, clone M1/70, 1:1000 (eBioscience, 25-0112-82, RRID:AB_469588)
anti-Gr1 APC, clone RB6-BC5, 1:2000 (eBioscience, 17-5931-82, RRID:AB_469476) or PE-Cy7, clone RB6-BC5, 1:2000 (eBioscience, 25-5931-82, RRID:AB_469663)
anti-Ter119 APC, clone TER-119, 1:300 (eBioscience, 17-5921-82, RRID:AB_469473) or PE-Cy7, clone TER-119, 1:1000 (eBioscience, 25-5921-82, RRID:AB_469661)
anti-CD117 (cKit) BV711, clone 2B8, 1:2000 (Biolegend, 105835, RRID:AB_2565956)
anti-Sca1 (Ly-6A/E) APC-Cy7, clone D7, 1:1000 (BD Biosciences, 560654, RRID:AB_1727552)
anti-CD150 PE-Cy5, clone TC15-12F12-2, 1:1000 (Biolegend, 115912, RRID:AB_493598) or APC, clone TC15-12F12-2, 1:1000 (Biolegend, 115910, RRID:AB_493460)
anti-CD48 PE-Cy7, clone HM48-1, 1:1000 (Biolegend, 103424, RRID:AB_2075049) or BUV395, clone HM48-1, 1:300 (BD, 740236, RRID:AB_2739984)
anti-CD34 eFluor450, clone RAM34, 1:30 (eBioscience, 48-0341-82, RRID:AB_2043837)
anti-CD45.1 PE, clone A20, 1:300 (eBioscience, 12-0453-82, RRID:AB_465675) or AF700, clone A20, 1:200 (Biolegend, 110724, RRID:AB_493733)
anti-CD45.2 AF700, clone 104, 1:100 (eBioscience, 56-0454-82, RRID:AB_657752)

Bone marrow committed progenitors analysis (validation cohort):
Anti-CD4 APC, clone GK1.5, 1:2000 (eBioscience, 17-0041-82) or PE-Cy7, GK1.5, 1:1000 (eBioscience, 25-0041-82)

Anti-CD8 APC, clone 53-6.7, 1:3000 (eBioscience, 17-0081-82) or PE-Cy7, clone 53-6.7, 1:1000 (eBioscience, 25-0081-82)
anti-B220 APC, clone RA3-6B2, 1:500 (eBioscience, 17-0452-82) or PE-Cy7, clone RA3-6B2, 1:500 (eBioscience, 25-0452-82)
anti-CD11b APC, clone M1/70, 1:2000 (eBioscience, 17-0112-82) or PE-Cy7, clone M1/70, 1:1000 (eBioscience, 25-0112-82)
anti-Gr1 APC, clone RB6-BC5, 1:2000 (eBioscience, 17-5931-82) or PE-Cy7, clone RB6-BC5, 1:2000 (eBioscience, 25-5931-82)
anti-Ter119 APC, clone TER-119, 1:300 (eBioscience, 17-5921-82) or PE-Cy7, clone TER-119, 1:1000 (eBioscience, 25-5921-82)
anti-CD117 (cKit) BV711, clone 2B8, 1:2000 (Biolegend, 105835, RRID:AB_2565956)
anti-Sca1 (Ly-6A/E) APC-Cy7, clone D7, 1:1000 (BD Biosciences, 560654, RRID:AB_1727552)
anti-CD16/32 APC, clone 93, 1:1000 (eBioscience, 17-0161-82, RRID:AB_469356) or BUV737, clone 2.4G2, 1:500 (BD Biosciences, 565272, RRID:AB_2739145)
anti-CD127 PE, clone A7R34, 1:100 (eBioscience, 12-1273-82, RRID:AB_953562) or PE-Cy7, clone A7R34, 1:200 (BD Biosciences, 560733, RRID:AB_1727424)
anti-CD34 eFluor450, clone RAM34, 1:30 (eBioscience, 48-0341-82, RRID:AB_2043837)
anti-CD45.1 AF700, clone A20, 1:200 (Biolegend, 110724, RRID:AB_493733)

Bone marrow stem cells and committed progenitors analysis (discovery cohort):
Anti-CD105 PacificBlue, clone MJ7/18, 1:300 (eBioscience, 14-1051-82, RRID:AB_467414)
Anti-CD127 PE-Cy7, clone A7R34, 1:200 (eBioscience, 25-1271-82, RRID:AB_469649)
Anti-CD135 PE, clone A2F10, 1:300 (eBioscience, 12-1351-82, RRID:AB_465859)
Anti-CD150 BV786, clone TC15-12F12.2, 1:1000 (BD Biosciences, 567518, RRID:AB_2916634)
Anti-CD16/32 BUV737, clone 2.4G2, 1:500 (BD Biosciences, 565272, RRID:AB_2739145)
anti-CD4 PE-Cy5, clone GK1.5, 1:2000 (eBioscience, 15-0041-82, RRID:AB_468695)
Anti-CD41 APC, clone MWReg30, 1:200 (Biolegend, 133914, RRID:AB_11125581)
Anti-CD48 BUV395 clone HM48-1, 1:300 (BD Biosciences, 740236, RRID:AB_2739984)
Anti-CD8 PE-Cy5, clone 53-6.7, 1:1000 (eBioscience, 15-0081-82, RRID:AB_468706)
Anti-Ter119 PE-Cy5, clone TER-119, 1:500 (eBioscience, 15-5921-82, RRID:AB_468810)
Anti-CD117 (cKit) BV711, clone 2B8, 1:2000 (Biolegend, 105835, RRID:AB_2565956)
Anti-CD45.1 AF700, clone A20, 1:200 (Biolegend, 110724, RRID:AB_493733)
Anti-CD45.2 PerCP-Cy5.5, clone 104, 1:200 (Biolegend, 109828, RRID:AB_893350)
Anti-CD11b PE-Cy5, clone M1/70, 1:3000 (eBioscience, 15-0112-82, RRID:AB_468714)
Anti-B220 PE-Cy5, clone RA3-6B2, 1:1000 (eBioscience, 15-0452-82, RRID:AB_468755)
Anti-Gr1 PE-Cy5, clone RB6-8C5, 1:3000 (eBioscience, 15-5931-82, RRID:AB_468813)
Anti-Sca1 APC-Cy7, clone D7, 1:1000 (BD Biosciences, 560654, RRID:AB_1727552)

Validation	All antibodies used in this study are commercially available, broadly established and validated by the respective manufacturers.

Animals and other research organisms

Policy information about studies involving animals; ARRIVE guidelines recommended for reporting animal research, and Sex and Gender in Research

Laboratory animals	Mice were housed in individually ventilated cages under specific pathogen-free (SPF) conditions at the Animal Facility of the German Cancer Research Center (DKFZ, Heidelberg), with ad libitum access to water and food (22±2°C, 45–65% humidity, 12h light–dark cycle). C57BL/6J mice, purchased from Janvier Laboratories, were used as recipients in transplantation experiments and were always 8 to 10 weeks old. All other mouse lines used in the study were bred in-house. UBC-GFP and KuOrange (KuO) mice, with a C57BL/6-Ly5.1 background, were used as donors for transplantation experiments and were 8 to 18 weeks old. Supportive bone marrow for transplantation assays was obtained from either C57BL/6J x Ly5.1 or Rag2-/- mice. C57BL/6J recipients were always females, while Rag2-/- recipients were both females and males.
Wild animals	No wild animals were used in this study.
Reporting on sex	Single-cell transplantations using C57BL/6J recipient mice were always performed with female recipients, with sex-matched donors. In transplantations involving the Rag2-/- mouse line, both female and male recipients were used, and sex-matching was not consistently applied.
Field-collected samples	No field-collected samples were used in this study.
Ethics oversight	All animal experiments were approved by the Animal Care and Use Committees of the German Regierungspräsidium Karlsruhe für Tierschutz und Arzneimittelüberwachung (Karlsruhe, Germany) under the animal permits (TVAs) G-41/19 and G-50/17.

Note that full information on the approval of the study protocol must also be provided in the manuscript.

Plants

Seed stocks

Report on the source of all seed stocks or other plant material used. If applicable, state the seed stock centre and catalogue number. If plant specimens were collected from the field, describe the collection location, date and sampling procedures.

Novel plant genotypes

Describe the methods by which all novel plant genotypes were produced. This includes those generated by transgenic approaches, gene editing, chemical/radiation-based mutagenesis and hybridization. For transgenic lines, describe the transformation method, the number of independent lines analyzed and the generation upon which experiments were performed. For gene-edited lines, describe the editor used, the endogenous sequence targeted for editing, the targeting guide RNA sequence (if applicable) and how the editor was applied.

Authentication

Describe any authentication procedures for each seed stock used or novel genotype generated. Describe any experiments used to assess the effect of a mutation and, where applicable, how potential secondary effects (e.g. second site T-DNA insertions, mosiacism, off-target gene editing) were examined.

Flow Cytometry

Plots

Confirm that:

☒ The axis labels state the marker and fluorochrome used (e.g. CD4-FITC).

☒ The axis scales are clearly visible. Include numbers along axes only for bottom left plot of group (a 'group' is an analysis of identical markers).

☒ All plots are contour plots with outliers or pseudocolor plots.

☒ A numerical value for number of cells or percentage (with statistics) is provided.

Methodology

Sample preparation

For the comprehensive immunophenotypic characterization, hematopoietic cells were collected from the peritoneal cavity (PerCav) in 2 mL PBS, and hematopoietic organs and tissues were dissected, including bones, spleen, lymph nodes (LNs), thymus, lung and liver. Bone marrow (BM) was harvested by isolating, cleaning and crushing the vertebral column, tibia, femur, limbs and sternum of sacrificed mice in RPMI + 2% FCS. Cell suspensions were filtered through a 40 µm cell strainer, centrifuged and resuspended in ACK buffer for red blood cell lysis for 3 minutes at room temperature (RT). Lungs and liver were minced into small pieces. Lungs were further filtered initially through a 100 µm and subsequently through a 70 µm cell strainer. Liver, LNs, spleen and thymus were filtered through a 40 µm cell strainer. Cell suspensions were spun down, resuspended in RPMI + 2% FCS and split for multiple flow cytometric analysis. Colons were turned inside out, cleaned and incubated in 25 mL extraction medium (RPMI 1640 + 2% FCS + 1 mM DTT + 0,5 mM EDTA) for 20 min at 37 °C to digest the intraepithelial layer. 1 mL FCS was then added to block the digestion, samples were filtered through a 40 µm cell strainer, centrifuged and resuspended in RPMI + 2% FCS for staining. If not stated otherwise, each step was performed on ice, RPMI or PBS supplemented with 2% FCS was used for washing and resuspending and centrifugation was done at 600 g, 4°C for 5 min. For the large-scale follow up cohorts, the same experimental protocol was followed for the isolation of bone marrow hematopoietic cells. Peripheral blood and bone marrow, spleen, lymph nodes, liver, lung, thymus, colon, peritoneal cavity cell suspensions were stained using monoclonal antibodies recognizing cell-specific surface proteins. Cells were incubated with an antibody mix prepared in PBS + 2% FBS. For organ-derived hematopoietic staining, cell suspensions had a concentration of 1x10^6 cells/10 µL antibody mix. For white blood cell staining, 50 µL peripheral blood was incubated with 100 µL antibody mix. Blood platelet and erythrocyte staining involved 3 µL peripheral blood and 27 µL antibody mix. Cells were incubated for 30 min at 4°C in the dark. All samples stained with antibodies against white blood cell epitopes were subjected to an erythrocyte lysis step using an ACK lysis buffer. Blood cells were incubated with ACK lysis buffer for 10 min, and remaining organ-derived hematopoietic cells were incubated with ACK lysis buffer for 2 min at room temperature. In case of the platelet and erythrocyte staining, this lysis step was not performed. After the lysis, cells were washed once with PBS + 2% FCS and resuspended in a final volume of PBS + 2% FCS. All samples were filtered prior to flow cytometry analysis.

Instrument

Cells were analyzed by flow cytometry using a LSRFortessa or a LSRII cytometer (BD Biosciences), both equipped with 350 nm, 405 nm, 488 nm, 561 nm and 641 nm excitation lasers. Similarly, sorting experiments were performed using a BD FACS Aria I or II (BD Biosciences). Each antibody panel was manually compensated using OneComp eBeads (eBioscience) stained with single antibodies.

Software

BD FACSDiva and FlowJo v10.7.1 were used to analyze flow cytometry data.

Cell population abundance

The final sorted population for single cell transplantation studies (phenotypically defined as Lineage-, Kit+, Sca1+, EPCRhi, CD34-, CD150+, CD48-) represented approximately 0.005% of the total single cells.

Gating strategy

FSC-A vs SSC-A was used to gate for the bulk population of cells. FSC-A vs FSC-H was used to exclude doublets. Sorting gating strategies are included as Supplementary Figures 2-13.

☒ Tick this box to confirm that a figure exemplifying the gating strategy is provided in the Supplementary Information.
