# Supplementary material for: A kinetics-based model of haematopoiesis reveals extrinsic regulation of skewed lineage output from stem cells
Source: Nat Cell Biol. 2026 May 25;28(6):1127–40. doi: 10.1038/s41556-026-01958-0 (PMC13278965; doi:10.1038/s41556-026-01958-0)
Supplement: Supplementary file 3 — Peer Review File [file 41556_2026_1958_MOESM3_ESM.pdf]

nature portfolio

Peer Review File

A kinetics-based model of hematopoiesis reveals extrinsic regulation of skewed lineage output from stem cells

Corresponding Author: Dr Michael Milsom

Version 0:

Decision Letter:

*Please delete the link to your author homepage if you wish to forward this email to co-authors.

Dear Mick,

Your manuscript, "A kinetics-based model of hematopoiesis reveals extrinsic regulation of skewed lineage output from stem cells", has now been seen by 3 referees, who are experts in HSC transplantation, hematopoiesis, scRNA-seq (referee 1); computational and mathematical modeling, clonal analysis (referee 2); and HSCs, transplantation (referee 3). As you will see from their comments (attached below) they find this work of potential interest, but have raised substantial concerns, which in our view would need to be addressed with considerable revisions before we can consider publication in Nature Cell Biology.

Nature Cell Biology editors discuss the referee reports in detail within the editorial team, including the chief editor, to identify key referee points that should be addressed with priority. To guide the scope of the revisions, I have listed these points below. I should stress that the referees' concerns (especially those by referee #1) point to a premature dataset and these points would need to be addressed with experiments and data, and reconsideration of the study for this journal and re-engagement of referees would largely depend on the strength of these revisions.

In particular, it would be essential to:

(A) Clarify the advance and differences the classification provided here presents over previous relevant work, as well as provide additional data to sufficiently support such claims, as indicated by referee #1:

"Fig.1g, the heterogeneity of HSC has been demonstrated by many studies based on large number of sc-transplantation analysis. However, the authors classified three types of clones based on the reconstitution kinetics of only 18 single cell clones. This n number is too small to get objective classification for the HSC clones, because some clones with low percentage would be lost due to the low number of sampling."

"Fig. 1d, the three clusters author classified fit well to the previous established classification (alpha, beta, gamma/delta). What are the advantages of this new classification?"

"Since slope is not very correlated with the level of final chimerism, it is not surprising and perhaps one could even say it is trivial that the longer the HSC continues to generate outputs the higher the final chimerism. In other words, that the key feature of Cluster 3 compared with Cluster 2 is prolonged tyMax and tGrowth is a tautological statement using math to state the obvious, that is, more enduring clones would be more dominant eventually."

"What is truly surprising is the positive correlation between t0 and final chimerism which is the only observed correlation that justifies the statement “competition between slow- and fast-engrafting clones” in the abstract. However, the authors did not provide evidence that variance in t0 is the main driving force behind the observed clonal difference in reconstitution behavior. The positive correlation between t0 and tyMax or tGrowth could be purely coincidental, and it is not a proof that t0 is the ultimate cause. To sum up, the role of t0 needs to be isolated and better studied because positive correlation between the tyMax and tGrowth and the level of final chimerism is almost synonymical."

"Could the author summarize the advantages of the new established classification of HSC clones based on the reconstitution kinetics rather than the arbitrary threshold and time points? What’s the new implication of this new classification could provide for the evaluation of HSC in the future?"

(B) Address concerns regarding conclusions about cell- intrinsic or extrinsic regulation, as indicated by:

Reviewer #1:
"The author drawn the main conclusion that “lineage biases actually arise from cell-extrinsic feedback regulation and clonal competition between slow- and fast-engrafting clones to occupy the limited compartment sizes of mature lineages”. How could the author exclude the intrinsic cellular differences among different clones?

Reviewer #3:
"A good model should be able to explain observed discrepancies.
1. How to coordinate the model of extrinsic feedback from the lineage demanding and dormant HSC model.
2. Compared to slow and fast HSC clones, which one is sensitive to chemotherapy, and which one can survive better under stress?
3. How to explain the behavior change from fast HSC clones to slow HSC clones upon transplantation.
4. Why is myeloid bias an aged HSC feature?"

(C) Provide all details and clarifications requested by reviewer #2 in terms of the mathematical modeling processes.

(D) All other referee concerns pertaining to strengthening existing data, textual changes, and experiments to answer specific questions and provide clarifications, should also be addressed.

(E) Finally, please pay close attention to our guidelines on statistical and methodological reporting (listed below) as failure to do so may delay the reconsideration of the revised manuscript. In particular please provide:

- unprocessed images of all gels/blots in the form of a multi-page pdf file. Please ensure that blots/gels are labeled and the sections presented in the figures are clearly indicated.

- all numerical source data in Excel format, with data for different figures provided as different sheets within a single Excel file. The file should include source data giving rise to graphical representations and statistical descriptions in the paper and for all instances where the figures present representative experiments of multiple independent repeats, the source data of all repeats should be provided.

We would be happy to consider a revised manuscript that would satisfactorily address these points, unless a similar paper is published elsewhere, or is accepted for publication in Nature Cell Biology in the meantime.

When revising the manuscript please:

- ensure that it conforms to our format instructions and publication policies (see below and https://www.nature.com/nature/for-authors).

- provide a point-by-point rebuttal to the full referee reports verbatim, as provided at the end of this letter.

- for any revision that includes light microscopy data, we ask our authors to please include a completed light microscopy reporting table https://www.nature.com/documents/Light_microscopy_reporting_table.xlsx to ensure the methods are described thoroughly. The table will be available to reviewers and ultimately published should the manuscript be accepted at the journal.

- provide the completed Reporting Summary (found here https://www.nature.com/documents/nr-reporting-summary.pdf). This is essential for reconsideration of the manuscript will be available to editors and referees in the event of peer review. For more information see http://www.nature.com/authors/policies/availability.html or contact me.

When submitting the revised version of your manuscript, please pay close attention to our href="https://www.nature.com/nature-portfolio/editorial-policies/image-integrity">Digital Image Integrity Guidelines. and to the following points below:

-- that unprocessed scans are clearly labelled and match the gels and western blots presented in figures.

-- that control panels for gels and western blots are appropriately described as loading on sample processing controls
-- all images in the paper are checked for duplication of panels and for splicing of gel lanes.

Finally, please ensure that you retain unprocessed data and metadata files after publication, ideally archiving data in perpetuity, as these may be requested during the peer review and production process or after publication if any issues arise.

Nature Cell Biology is committed to improving transparency in authorship. As part of our efforts in this direction, we are now requesting that all authors identified as ‘corresponding author’ on published papers create and link their Open Researcher and Contributor Identifier (ORCID) with their account on the Manuscript Tracking System (MTS), prior to acceptance. ORCID helps the scientific community achieve unambiguous attribution of all scholarly contributions. You can create and link your ORCID from the home page of the MTS by clicking on ‘Modify my Springer Nature account’. For more information please visit please visit www.springernature.com/orcid.

This journal strongly supports public availability of data. Please place the data used in your paper into a public data repository, or alternatively, present the data as Supplementary Information. If data can only be shared on request, please explain why in your Data Availability Statement, and also in the correspondence with your editor. Please note that for some data types, deposition in a public repository is mandatory - more information on our data deposition policies and available repositories appears below.

Please submit the revised manuscript files and the point-by-point rebuttal to the referee comments using this link:

Link Redacted

*This url links to your confidential home page and associated information about manuscripts you may have submitted or be reviewing for us. If you wish to forward this email to co-authors, please delete the link to your homepage.

We would like to receive a revised submission within six months.

We hope that you will find our referees' comments, and editorial guidance helpful. Please do not hesitate to contact me if there is anything you would like to discuss.

Best wishes,

Stelios

Stylianos Lefkopoulos, PhD
He/him/his
Senior Editor, Nature Cell Biology
Springer Nature
Heidelberger Platz 3, 14197 Berlin, Germany

E-mail: stylianos.lefkopoulos@springernature.com
Bluesky: @slefkopoulos.bsky.social
LinkedIn: www.linkedin.com/in/stylianos-lefkopoulos-81b007a0

Reviewers' Comments:

Reviewer #1 (Remarks to the Author):

The manuscript by Rodríguez-Correa et al. demonstrated that the reconstitution kinetics served as an overall unifying metric of HSC functional potency, with the most potent HSCs displaying the greatest delay in hematopoietic reconstitution. Based on serial single cell transplantation, they revealed a unidirectional acceleration in reconstitution kinetics accompanied by a gradual decline in functional potency of daughter HSCs. Furthermore, they combined mathematically model and experimental results and draw the conclusion that lineage biases actually arise from cell-extrinsic feedback regulation and clonal competition between slow- and fast-engrafting clones to occupy the limited compartment sizes of mature lineages. Although with large amount of work, this manuscript is difficult to understand due to some unclear labeling and unreasonable rationales to explain the data. Several important concerns must be addressed before publication.

Major
1. -The first section (Figure 1) seems somewhat disconnected from the rest of the content. There is nothing lose without the Figure 1. What is the role of Figure 1 in the total story?

2. Fig. 1a, it is interesting to detect the chimerism in other organs (liver, lung, and colon, et al) apart from hematopoietic tissues (BM, SP and PB) after single cell transplantation. What are the differences of the chimerism in these organs compared with the classical hematopoietic tissues? What is the additional information provided from these detections?
3. -Fig.1g, the heterogeneity of HSC has been demonstrated by many studies based on large number of sc-transplantation analysis. However, the authors classified three types of clones based on the reconstitution kinetics of only 18 single cell clones. This n number is too small to get objective classification for the HSC clones, because some clones with low percentage would be lost due to the low number of sampling.
4. -Fig. 1d, the three clusters author classified fit well to the previous established classification (alpha, beta, gamma/delta). What are the advantages of this new classification?
5. -Fig. 2: Please clearly label the unit for each horizontal axis. Presumably 'days' for t0 and 'weeks' for other parameters such as tyMax?
6. -Fig. 2: Since slope is not very correlated with the level of final chimerism, it is not surprising and perhaps one could even say it is trivial that the longer the HSC continues to generate outputs the higher the final chimerism. In other words, that the key feature of Cluster 3 compared with Cluster 2 is prolonged tyMax and tGrowth is a tautological statement using math to state the obvious, that is, more enduring clones would be more dominant eventually.
What is truly surprising is the positive correlation between t0 and final chimerism which is the only observed correlation that justifies the statement "competition between slow- and fast-engrafting clones" in the abstract. However, the authors did not provide evidence that variance in t0 is the main driving force behind the observed clonal difference in reconstitution behavior. The positive correlation between t0 and tyMax or tGrowth could be purely coincidental, and it is not a proof that t0 is the ultimate cause. To sum up, the role of t0 needs to be isolated and better studied because positive correlation between the tyMax and tGrowth and the level of final chimerism is almost synonymical.
7. -Fig. 2a-b, the authors showed the parameters related to reconstitution kinetics of single clones after transplantation. Do the clones from distinct clusters (cluster 1/2/3) have the similar reconstitution kinetics? or have different parameters? Furthermore, what about the correlation analysis?
8. -Fig. 2d, what was the outcome of co-transplanting GFP+ and KuOrange+ paired cells? Did they exhibit similar reconstitution patterns over time? Given that these cells shared the same initial niche, is there any observed correlation in their clonal behavior?
9. -Fig. 3a, what is the exact standard to classify clones into five different subtypes (clone I-V)? Please clarify the standard to define these five clonal systems. More importantly, what is the relationship of these clone I-V and the cluster 1-3 in Fig.1?
10. -Fig. 3, what are the distinct features of clones I–V or slow-fast clones based on the sequencing data? Are they regulated by specific genes, pathways?
11. What are the mature blood cell levels for the Rag2 knockout (Rag2-/-) recipients and the different fast or slow recipients? (Can they keep a low Coefficient of variation at 24 weeks?)
12. What is fast and slow composition in the B6 or Rag2-/- systems? Based on Figure 5e, most clones are fast and were not affected by the competition.
13. What about reconstitution levels in these different clones (including: I-V and fast or slow)?
14. Could the author summarize the advantages of the new established classification of HSC clones based on the reconstitution kinetics rather than the arbitrary threshold and time points? What's the new implication of this new classification could provide for the evaluation of HSC in the future?
15. The author drawn the main conclusion that "lineage biases actually arise from cell-extrinsic feedback regulation and clonal competition between slow- and fast-engrafting clones to occupy the limited compartment sizes of mature lineages". How could the author exclude the intrinsic cellular differences among different clones?
Minor:
1. In Figure 1, the author mentioned 2nd BMT, but the corresponding reconstitution data are not shown. What happened to the 3 clusters after the 2nd BMT?
2. It is unclear whether Figure 1 presents data from 22 clones (comprising 18 single-clone and 4 polyclonal controls), or from 22 mice with sustained donor chimerism (>0.1%). Please clarify the total number of animals analyzed and how these numbers relate to the clone definitions.
3. The authors mention the mean chimerism across 55 cell types. What is the biological relevance of this mean value?
4. Please explain what Dim 4 and Dim 5 represent in the analysis.
5. Supplementary Figure 2b and 2e: Please consider showing the overall chimerism for each clone in these supplementary figures.
6. In Figure 3e, the log2 fold change (log2FC) in MkP cell abundance appears too small to be biologically meaningful. Could the authors comment on this observation or provide statistical support?
7. Please consider labeling fast and slow clones directly in Figure 5d to facilitate interpretation and comparison.
8. Please clarify how fast and slow clones were defined (by t0 < 6? The author only checked the blood at weeks 4, 8, 12, 16, 20, and 24.)

Reviewer #2 (Remarks to the Author):

Rodríguez-Correa, Grünschläger, Nizharadze et al., present a well-crafted manuscript describing their work in characterizing hematopoietic stem cell behavior following transplantation by tracking the reconstitution dynamics of single HSC transplants. The authors present a very interesting and conceptually novel view of "lineage bias", showing that that the composition of descendant lineages of individual clones varies over time and that it may be inaccurate or even misleading to diagnose lineage bias based on a single time point. They also find that reconstitution kinetics correlate with overall potency, with the most potent clones displaying the most delayed reconstitution. I really enjoyed this paper and felt that the authors' rigorous quantitative approach, along with extensive experimentation, was very satisfying. I strongly support publication with some minor revisions. My main comments pertain to the mathematical model as editorially requested, but I also offer some

additional questions and suggestions.

In Figure 4 and the corresponding text sections, the authors introduce a mathematical model intended to describe HSC contribution to blood formation over time, incorporating functional heterogeneity, lineage bias, and self-renewal potential within the HSC compartment. However, the current schematic in Figure 4 and the accompanying ODEs (line 1024 onward) do not provide sufficient detail for the reader to interpret the model's variables or assess the biological relevance of its assumptions. Assuming that HSCU represents the most primitive, slow-engrafting, and self-renewing HSC state; HSCM an intermediate, transitioning state; and HSCD a more active, rapidly engrafting, and differentiation-prone state, the authors should clarify several key points. Specifically:
a. Explain the logic for assuming that HSCU frequency remains constant over time.
b. How sensitive is the model's downstream behavior to the fixed value of HSCU's frequency?
c. Explain what do the n = 10 downstream populations represent, how was this number chosen and discuss how sensitive are the model's results to this assumption.
d. Finally, how are "fast" and "slow" clones defined for the comparison in Figure 4b—via model classification or based on empirical t_0 (time to first detection)?
Providing answers to these questions and more explicit variable definitions would improve accessibility and allow readers to more critically evaluate the model.

The mathematical model in figure 5 is almost not required as it makes a commonsense point, but it's still nice to have a formal treatment of the argument. I feel that it would be interesting to see the actual underlying data (as opposed to "merely" the coefficient of variation), as I don't have a very good intuitive sense of where the variation in the HSC compartment size really comes from, perhaps the authors could explain that a bit more as well. Is it just a sampling issue, i.e. there are so few HSCs in 150,000 supporting bone marrow cells that the final engraftment varies substantially by chance? Can the authors comment on this and explain how many HSCs they think there are in the support cells? I think that understanding this better would help the reader evaluate this section.
The authors make a compelling (and very intuitive) point, namely that a clone's lineage bias is not just hard-wired but responsive to peripheral demand. I think it would be helpful if the authors could provide a more transparent discussion of which clone properties they think are more intrinsically determined vs. extrinsically influenced, and to what degree. I gather from the text that they consider the reconstitution kinetics more hard-wired and the lineage output more malleable and responsive to outside demand, but since this is a central theme of the manuscript, I would appreciate a more direct discussion. Ideally, figure 5 would feature some sort of schematic/cartoon to help the reader synthesize the various strands of evidence from the paper.

Minor Comments
Line 104: There are prior mechanistic insights into the origin of lineage biases that may be worth citing.
Line 340: It would be beneficial for the reader if the discussion of the scRNAseq data covers in more detail what the authors' interpretation of these clones' behavior is, in connection to the transcriptomic signatures. Particularly, the pseudotime analysis is not discussed in the main text. More broadly, it would be helpful if the authors could explain what part of the scRNAseq data suggests that the distinct HSC clonal behaviors in the five assayed systems are due to plastic HSC output, shaped by reconstitution kinetics and not exhibited pre-existing HSC clonal heterogeneity.
In figure 1, it would have been helpful for me to have a little panel summarizing the properties of the clusters. I kept referring back to the text to try and remember what the characteristics of cluster were in order not to get lost and it was pretty laborious. I found the ternary plot of little help in that regard…

Signed, Kamila Naxerova

Reviewer #3 (Remarks to the Author):

Hematopoietic stem cells (HSCs) are defined by self-renewal ability and multi-lineage potential. However, accumulated observations from single-cell transplantation and scRNA-sequencing have revealed significant heterogeneity among HSCs, even sorted with all known possible HSC markers. To address the discrepancy between the traditional view of HSCs and this observed heterogeneity, this study conducted large-scale single-cell transplantation combined with clonal progeny analysis, thus developing a new model to explain HSC behavior in terms of their lineage output. The kinetic-based lineage reconstruction identified slow and fast lineage-generating HSC clones. Fast clones rapidly expand to meet the demand for certain lineage compartments, thus declining in functionality. In contrast, slow clones (similar to so-called dormant HSCs) are based on feedback to fill the reduced lineage compartment, experience less expansion, and thus preserve better functionality. Lineage bias is not previously thought an intrinsic feature but rather a response to external feedback from certain lineage compartments. This is a novel view for HSCs and is supported by strong data. There are some issues regarding this concept.
Issues
A good model should be able to explain observed discrepancies.
1. How to coordinate the model of extrinsic feedback from the lineage demanding and dormant HSC model.
2. Compared to slow and fast HSC clones, which one is sensitive to chemotherapy, and which one can survive better under stress?
3. How to explain the behavior change from fast HSC clones to slow HSC clones upon transplantation.
4. Why is myeloid bias an aged HSC feature?

GUIDELINES FOR MANUSCRIPT SUBMISSION TO NATURE CELL BIOLOGY

READABILITY OF MANUSCRIPTS – Nature Cell Biology is read by cell biologists from diverse backgrounds, many of whom are not native English speakers. Authors should aim to communicate their findings clearly, explaining technical jargon that might be unfamiliar to non-specialists, and avoiding non-standard abbreviations. Titles and abstracts should concisely communicate the main findings of the study, and the background, rationale, results and conclusions should be clearly explained in the manuscript in a manner accessible to a broad cell biology audience. Nature Cell Biology uses British spelling.

MANUSCRIPT FORMAT – please follow the guidelines listed in our Guide to Authors regarding manuscript formats at Nature Cell Biology.

TITLE – should be no more than 100 characters including spaces, without punctuation and avoiding technical terms, abbreviations, and active verbs..

AUTHOR NAMES – should be given in full.

AUTHOR AFFILIATIONS – should be denoted with numerical superscripts (not symbols) preceding the names. Full addresses should be included, with US states in full and providing zip/post codes. The corresponding author is denoted by: "Correspondence should be addressed to [initials]."

ABSTRACT AND MAIN TEXT – please follow the guidelines that are specific to the format of your manuscript, as listed in our Guide to Authors (http://www.nature.com/ncb/pdf/ncb_gta.pdf) Briefly, Nature Cell Biology Articles, Resources and Technical Reports have 3500 words, including a 150 word abstract, and the main text is subdivided in Introduction, Results, and Discussion sections. Nature Cell Biology Letters have up to 2500 words, including a 180 word introductory paragraph (abstract), and the text is not subdivided in sections.

ACKNOWLEDGEMENTS – should be kept brief. Professional titles and affiliations are unnecessary. Grant numbers can be listed.

AUTHOR CONTRIBUTIONS – must be included after the Acknowledgements, detailing the contributions of each author to the paper (e.g. experimental work, project planning, data analysis etc.). Each author should be listed by his/her initials.

FINANCIAL AND NON-FINANCIAL COMPETING INTERESTS – the authors must include one of three declarations: (1) that they have no financial and non-financial competing interests; (2) that they have financial and non-financial competing interests; or (3) that they decline to respond, after the Author Contributions section. This statement will be published with the article, and in cases where financial and non-financial competing interests are declared, these will be itemized in a web supplement to the article. For further details please see https://www.nature.com/licenceforms/nrg/competing-interests.pdf.

REFERENCES – are limited to a total of 70 for Articles, Resources, Technical Reports; and 40 for Letters. This includes references in the main text and Methods combined. References must be numbered sequentially as they appear in the main text, tables and figure legends and Methods and must follow the precise style of Nature Cell Biology references. References only cited in the Methods should be numbered consecutively following the last reference cited in the main text. References only associated with Supplementary Information (e.g. in supplementary legends) do not count toward the total reference limit and do not need to be cited in numerical continuity with references in the main text. Only published papers can be cited, and each publication cited should be included in the numbered reference list, which should include the manuscript titles. Footnotes are not permitted.

METHODS – Nature Cell Biology publishes methods online. The methods section should be provided as a separate Word document, which will be copyedited and appended to the manuscript PDF, and incorporated within the HTML format of the paper.

Methods should be written concisely, but should contain all elements necessary to allow interpretation and replication of the results. As a guideline, Methods sections typically do not exceed 3,000 words. The Methods should be divided into subsections listing reagents and techniques. When citing previous methods, accurate references should be provided and any alterations should be noted. Information must be provided about: antibody dilutions, company names, catalogue numbers and clone numbers for monoclonal antibodies; sequences of RNAi and cDNA probes/primers or company names and catalogue numbers if reagents are commercial; cell line names, sources and information on cell line identity and authentication. Animal studies and experiments involving human subjects must be reported in detail, identifying the committees approving the protocols. For studies involving human subjects/samples, a statement must be included confirming that informed consent was obtained. Statistical analyses and information on the reproducibility of experimental results should be provided in a section titled “Statistics and Reproducibility”.

All Nature Cell Biology manuscripts submitted on or after March 21 2016 must include a Data availability statement as a separate section after Methods but before references, under the heading "Data Availability”. . For Springer Nature policies on data availability see http://www.nature.com/authors/policies/availability.html; for more information on this particular policy see http://www.nature.com/authors/policies/data/data-availability-statements-data-citations.pdf. The Data availability statement should include:

• Accession codes for primary datasets (generated during the study under consideration and designated as "primary accessions") and secondary datasets (published datasets reanalysed during the study under consideration, designated as "referenced accessions"). For primary accessions data should be made public to coincide with publication of the manuscript. A list of data types for which submission to community-endorsed public repositories is mandated (including sequence, structure, microarray, deep sequencing data) can be found here http://www.nature.com/authors/policies/availability.html#data.

• Unique identifiers (accession codes, DOIs or other unique persistent identifier) and hyperlinks for datasets deposited in an approved repository, but for which data deposition is not mandated (see here for details http://www.nature.com/sdata/data-policies/repositories).

• At a minimum, please include a statement confirming that all relevant data are available from the authors, and/or are included with the manuscript (e.g. as source data or supplementary information), listing which data are included (e.g. by figure panels and data types) and mentioning any restrictions on availability.

• If a dataset has a Digital Object Identifier (DOI) as its unique identifier, we strongly encourage including this in the Reference list and citing the dataset in the Methods.

We recommend that you upload the step-by-step protocols used in this manuscript to protocols.io. More details can found at https://www.protocols.io/help/publish-articles.

DISPLAY ITEMS – main display items are limited to 6-8 main figures and/or main tables for Articles, Resources, Technical Reports; and 5 main figures and/or main tables for Letters. For Supplementary Information see below.

FIGURES – Colour figure publication costs $600 for the first, and $300 for each subsequent colour figure. All panels of a multi-panel figure must be logically connected and arranged as they would appear in the final version. Unnecessary figures and figure panels should be avoided (e.g. data presented in small tables could be stated briefly in the text instead).

All imaging data should be accompanied by scale bars, which should be defined in the legend.
Cropped images of gels/blots are acceptable, but need to be accompanied by size markers, and to retain visible background signal within the linear range (i.e. should not be saturated). The boundaries of panels with low background have to be demarked with black lines. Splicing of panels should only be considered if unavoidable, and must be clearly marked on the figure, and noted in the legend with a statement on whether the samples were obtained and processed simultaneously. Quantitative comparisons between samples on different gels/blots are discouraged; if this is unavoidable, it should only be performed for samples derived from the same experiment with gels/blots were processed in parallel, which needs to be stated in the legend.

Figures should be provided at approximately the size that they are to be printed at (single column is 86 mm, double column is 170 mm) and should not exceed an A4 page (8.5 x 11"). Reduction to the scale that will be used on the page is not necessary, but multi-panel figures should be sized so that the whole figure can be reduced by the same amount at the smallest size at which essential details in each panel are visible. In the interest of our colour-blind readers we ask that you avoid using red and green for contrast in figures. Replacing red with magenta and green with turquoise are two possible colour-safe alternatives. Lines with widths of less than 1 point should be avoided. Sans serif typefaces, such as Helvetica (preferred) or Arial should be used. All text that forms part of a figure should be rewritable and removable.

We accept files from the following graphics packages in either PC or Macintosh format:

- For line art, graphs, charts and schematics we prefer Adobe Illustrator (.AI), Encapsulated PostScript (.EPS) or Portable Document Format (.PDF). Files should be saved or exported as such directly from the application in which they were made, to allow us to restyle them according to our journal house style.

- We accept PowerPoint (.PPT) files if they are fully editable. However, please refrain from adding PowerPoint graphical effects to objects, as this results in them outputting poor quality raster art. Text used for PowerPoint figures should be Helvetica (preferred) or Arial.

- We do not recommend using Adobe Photoshop for designing figures, but we can accept Photoshop generated (.PSD or .TIFF) files only if each element included in the figure (text, labels, pictures, graphs, arrows and scale bars) are on separate layers. All text should be editable in ‘type layers’ and line-art such as graphs and other simple schematics should be preserved and embedded within 'vector smart objects’ - not flattened raster/bitmap graphics.

- Some programs can generate Postscript by 'printing to file' (found in the Print dialogue). If using an application not listed

above, save the file in PostScript format or email our Art Editor, Allen Beattie for advice (a.beattie@nature.com).

Regardless of format, all figures must be vector graphic compatible files, not supplied in a flattened raster/bitmap graphics format, but should be fully editable, allowing us to highlight/copy/paste all text and move individual parts of the figures (i.e. arrows, lines, x and y axes, graphs, tick marks, scale bars etc.). The only parts of the figure that should be in pixel raster/bitmap format are photographic images or 3D rendered graphics/complex technical illustrations.

All placed images (i.e. a photo incorporated into a figure) should be on a separate layer and independent from any superimposed scale bars or text. Individual photographic images must be a minimum of 300+ DPI (at actual size) or kept constant from the original picture acquisition and not decreased in resolution post image acquisition. All colour artwork should be RGB format.

EXTENDED DATA FIGURES - When re-submitting your manuscript, please ensure that any supplementary figures and tables that are crucial to the manuscript's conclusions are converted into Extended Data figures and tables to increase visibility of these data. Extended Data figures and tables are online-only (present in the online PDF and full-text HTML versions of the paper), peer-reviewed display items that provide essential background to the article but are not included in the main article due to space constraints. A maximum of ten Extended Data display items (figures and tables) is permitted.

FIGURE LEGENDS – must not exceed 350 words for each figure to allow fit on a single printed NCB page together with the figure. They must include a brief title for the whole figure, and short descriptions of each panel with definitions of the symbols used, but without detailing methodology.

TABLES – main tables should be provided as individual Word files, together with a brief title and legend. For supplementary tables see below.

SUPPLEMENTARY INFORMATION – Supplementary information is material directly relevant to the conclusion of a paper, but which cannot be included in the printed version in order to keep the manuscript concise and accessible to the general reader. Supplementary information is an integral part of a Nature Cell Biology publication and should be prepared and presented with as much care as the main display item, but it must not include non-essential data or text, which may be removed at the editor's discretion. All supplementary material is fully peer-reviewed and published online as part of the HTML version of the manuscript. Supplementary Figures and Supplementary Notes are appended at the end of the main PDF of the published manuscript.

Supplementary items should relate to a main text figure, wherever possible, and should be mentioned sequentially in the main manuscript, designated as Supplementary Figure, Table, Video, or Note, and numbered continuously (e.g. Supplementary Figure 1, Supplementary Figure 2, Supplementary Table 1, Supplementary Table 2 etc.).

Unprocessed scans of all key data generated through electrophoretic separation techniques need to be presented in a supplementary figure that should be labelled and numbered as the final supplementary figure, and should be mentioned in every relevant figure legend. This figure does not count towards the total number of figures and is the only figure that can be displayed over multiple pages, but should be provided as a single file, in PDF or TIFF format. Data in this figure can be displayed in a relatively informal style, but size markers and the figures panels corresponding to the presented data must be indicated.

The total number of Supplementary Figures (not including the "unprocessed scans" Supplementary Figure) should not exceed the number of main display items (figures and/or tables (see our Guide to Authors and March 2012 editorial http://www.nature.com/ncb/authors/submit/index.html#suppinfo; http://www.nature.com/ncb/journal/v14/n3/index.html#ed). No restrictions apply to Supplementary Tables or Videos, but we advise authors to be selective in including supplemental data.

Each Supplementary Figure should be provided as a single page and as an individual file in one of our accepted figure formats and should be presented according to our figure guidelines (see above). Supplementary Tables should be provided as individual Excel files. Supplementary Videos should be provided as .avi or .mov files up to 50 MB in size. Supplementary Figures, Tables and Videos much be accompanied by a separate Word document including titles and legends.

GUIDELINES FOR EXPERIMENTAL AND STATISTICAL REPORTING

REPORTING REQUIREMENTS – We are trying to improve the quality of methods and statistics reporting in our papers. To that end, we are now asking authors to complete a reporting summary that collects information on experimental design and reagents. The Reporting Summary can be found here https://www.nature.com/documents/nr-reporting-summary.pdf)If you would like to reference the guidance text as you complete the template, please access these flattened versions at http://www.nature.com/authors/policies/availability.html.

STATISTICS – Wherever statistics have been derived the legend needs to provide the n number (i.e. the sample size used to derive statistics) as a precise value (not a range), and define what this value represents. Error bars need to be defined in the legends (e.g. SD, SEM) together with a measure of centre (e.g. mean, median). Box plots need to be defined in terms of minima, maxima, centre, and percentiles. Ranges are more appropriate than standard errors for small data sets. Wherever

statistical significance has been derived, precise p values need to be provided and the statistical test used needs to be stated in the legend. Statistics such as error bars must not be derived from n<3. For sample sizes of n<5 please plot the individual data points rather than providing bar graphs. Deriving statistics from technical replicate samples, rather than biological replicates is strongly discouraged. Wherever statistical significance has been derived, precise p values need to be provided and the statistical test stated in the legend.

Information on how many times each experiment was repeated independently with similar results needs to be provided in the legends and/or Methods for all experiments, and in particular wherever representative experiments are shown.

We strongly recommend the presentation of source data for graphical and statistical analyses as a separate Supplementary Table, and request that source data for all independent repeats are provided when representative experiments of multiple independent repeats, or averages of two independent experiments are presented. This supplementary table should be in Excel format, with data for different figures provided as different sheets within a single Excel file. It should be labelled and numbered as one of the supplementary tables, titled “Statistics Source Data”, and mentioned in all relevant figure legends.

--------- Please don't hesitate to contact NCB@nature.com should you have queries about any of the above requirements --------

Version 1:

Decision Letter:

23rd February 2026

Dear Mick,

Thank you for submitting your revised manuscript "A kinetics-based model of hematopoiesis reveals extrinsic regulation of skewed lineage output from stem cells" (NCB-A58743A). It has now been seen by the original referees and their comments are below. The reviewers find that the paper has improved in revision, and therefore we'll be happy in principle to publish it in Nature Cell Biology, pending minor revisions to satisfy the referees' final requests and to comply with our editorial and formatting guidelines.

If the current version of your manuscript is in a PDF format, please email us a copy of the file in an editable format (Microsoft Word or LaTex)-- we cannot proceed with PDFs at this stage.

We are now performing detailed checks on your paper and will send you a checklist detailing our editorial and formatting requirements in about a week. Please do not upload the final materials and make any revisions until you receive this additional information from us.

Thank you again for your interest in Nature Cell Biology. Please do not hesitate to contact me if you have any questions.

Best wishes,
Stelios

Dr. Stylianos Lefkopoulos
He/him/his
Senior Editor, Nature Cell Biology
Springer Nature
Heidelberger Platz 3, 14197 Berlin, Germany

E-mail: stylianos.lefkopoulos@springernature.com
Bluesky: @slefkopoulos.bsky.social
LinkedIn: www.linkedin.com/in/stylianos-lefkopoulos-81b007a0

Reviewer #1 (Remarks to the Author):

The authors has responded all of my comments.
I have no other question.
The last suggestion is please organize the story to make it clearer and easy-understood for the hematologists.

Reviewer #2 (Remarks to the Author):

I thank the authors for their excellent replies and have no further questions. I look forward to seeing the article published.

Reviewer #3 (Remarks to the Author):

The authors have adequately addressed my comments. Given that most of the observations were derived from injury/emergency conditions, the authors should clarify that the proposed model may not reflect HSC homeostatic kinetics.

Version 2:

Decision Letter:

Dear Mick,

I am pleased to inform you that your manuscript, "A kinetics-based model of hematopoiesis reveals extrinsic regulation of skewed lineage output from stem cells", has now been accepted for publication in Nature Cell Biology. Congratulations!

Thank you for sending us the final manuscript files to be processed for print and online production, and for returning the manuscript checklists and other forms. Your manuscript will now be passed to our production team who will be in contact with you if there are any questions with the production quality of supplied figures and text.

Over the next few weeks, your paper will be copyedited to ensure that it conforms to Nature Cell Biology style. Once your paper is typeset, you will receive an email with a link to choose the appropriate publishing options for your paper and our Author Services team will be in touch regarding any additional information that may be required.

After the grant of rights is completed, you will receive a link to your electronic proof via email with a request to make any corrections within 48 hours. If, when you receive your proof, you cannot meet this deadline, please inform us at rjsproduction@springernature.com immediately.

You will not receive your proofs until the publishing agreement has been received through our system.

Due to the importance of these deadlines, we ask that you please let us know now whether you will be difficult to contact over the next month. If this is the case, we ask you provide us with the contact information (email, phone and fax) of someone who will be able to check the proofs on your behalf, and who will be available to address any last-minute problems.

If you have any questions about our publishing options, costs, Open Access requirements, or our legal forms, please contact ASJournals@springernature.com

Once your paper has been scheduled for online publication, the Nature press office will be in touch to confirm the details. An online order form for reprints of your paper is available at https://www.nature.com/reprints/author-reprints.html. All co-authors, authors' institutions and authors' funding agencies can order reprints using the form appropriate to their geographical region.

Publication is conditional on the manuscript not being published elsewhere and on there being no announcement of this work to any media outlet until the online publication date in Nature Cell Biology.

You may wish to make your media relations office aware of your accepted publication, in case they consider it appropriate to organize some internal or external publicity. Once your paper has been scheduled you will receive an email confirming the publication details. This is normally 3-4 working days in advance of publication. If you need additional notice of the date and time of publication, please let the production team know when you receive the proof of your article to ensure there is sufficient time to coordinate. Further information on our embargo policies can be found here: https://www.nature.com/authors/policies/embargo.html

Please note that *Nature Cell Biology* is a Transformative Journal (TJ). Authors may publish their research with us through the traditional subscription access route or make their paper immediately open access through payment of an article-processing charge (APC). Authors will not be required to make a final decision about access to their article until it has been accepted. Find out more about Transformative Journals

Authors may need to take specific actions to achieve compliance with funder and institutional open access mandates. If your research is supported by a funder that requires immediate open access (e.g. according to Plan S principles or the NIH public access policy) then you should select the gold OA route, and we will direct you to the compliant route where possible. Because authors warrant under our subscription licensing terms that they haven’t committed to licensing any version of their article under a licence inconsistent with the terms of our agreement – including the applicable embargo period – publication under the subscription model isn’t suitable for authors whose funders require no embargo.

To assist our authors in disseminating their research to the broader community, our SharedIt initiative provides you with a

unique shareable link that will allow anyone (with or without a subscription) to read the published article. Recipients of the link with a subscription will also be able to download and print the PDF.

If your paper includes color figures, please be aware that in order to help cover some of the additional cost of four-color reproduction, Nature Portfolio charges our authors a fee for the printing of their color figures. Please contact our offices for exact pricing and details.

As soon as your article is published, you will receive an automated email with your shareable link.

If you have not already done so, we strongly recommend that you upload the step-by-step protocols used in this manuscript to protocols.io (https://protocols.io), an open online resource that allows researchers to share their detailed experimental know-how. All uploaded protocols are made freely available and are assigned DOIs for ease of citation. Protocols and Nature Portfolio journal papers in which they are used can be linked to one another, and this link is clearly and prominently visible in the online versions of both. Authors who performed the specific experiments can act as primary authors for the Protocol as they will be best placed to share the methodology details, but the Corresponding Author of the present research paper should be included as one of the authors. By uploading your Protocols onto protocols.io, you are enabling researchers to more readily reproduce or adapt the methodology you use, as well as increasing the visibility of your protocols and papers. You can also establish a dedicated workspace to collect your lab Protocols. Further information can be found at https://www.protocols.io/help/publish-articles.

You can use a single sign-on for all your accounts, view the status of all your manuscript submissions and reviews, access usage statistics for your published articles and download a record of your refereeing activity for the Nature Portfolio.

Nature Cell Biology encourages authors presenting evidence for cell, biological, molecular, and genetic interactions to consider communicating these findings using Biofactoid (https://biofactoid.org/). This tool helps users share a searchable representation of interactions (e.g. binding, gene expression, post-translational modification) between genes, gene products, or chemicals. Information added to Biofactoid, with author attribution, is shared on social media and public databases, such as Pathway Commons, where it can be discovered and analyzed in the context of a large and growing corpus of knowledge.

Please feel free to contact us if you have any questions.

With kind regards,
Stelios

Dr. Stylianos Lefkopoulos
He/him/his
Senior Editor, Nature Cell Biology
Springer Nature
Heidelberger Platz 3, 14197 Berlin, Germany

E-mail: stylianos.lefkopoulos@springernature.com
Bluesky: @slefkopoulos.bsky.social
LinkedIn: www.linkedin.com/in/stylianos-lefkopoulos-81b007a0

Click here if you would like to recommend Nature Cell Biology to your librarian
http://www.nature.com/subscriptions/recommend.html#forms

** Visit the Springer Nature Editorial and Publishing website at www.springernature.com/editorial-and-publishing-jobs for more information about our career opportunities. If you have any questions please click here.**

Open Access This Peer Review File is licensed under a Creative Commons Attribution 4.0 International License, which permits use, sharing, adaptation, distribution and reproduction in any medium or format, as long as you give appropriate credit to the original author(s) and the source, provide a link to the Creative Commons license, and indicate if changes were made.
In cases where reviewers are anonymous, credit should be given to 'Anonymous Referee' and the source.
The images or other third party material in this Peer Review File are included in the article's Creative Commons license, unless indicated otherwise in a credit line to the material. If material is not included in the article's Creative Commons license and your intended use is not permitted by statutory regulation or exceeds the permitted use, you will need to obtain permission directly from the copyright holder.
To view a copy of this license, visit https://creativecommons.org/licenses/by/4.0/
